# First-line immune checkpoint inhibitors in low programmed death-ligand 1-expressing population

**DOI:** 10.3389/fphar.2024.1377690

**Published:** 2024-07-26

**Authors:** Feiyang Zhang, Guoming Chen, Yixin Yin, Xiaojiang Chen, Runcong Nie, Yingbo Chen

**Affiliations:** ^1^ Department of Gastric Surgery, Sun Yat-sen University Cancer Center, State Key Laboratory of Oncology in South China, Collaborative Innovation Center for Cancer Medicine, Guangzhou, China; ^2^ Department of Pathology, Sun Yat-sen University Cancer Center, State Key Laboratory of Oncology in South China, Collaborative Innovation Center for Cancer Medicine, Guangzhou, China

**Keywords:** PD-1, PD-L1, immunotherapy, survival, first-line

## Abstract

**Introduction:** Inhibitors of programmed cell death 1 (PD1) and its ligand (PDL1) have exhibited favorable long-term survival in many types of advanced-stage cancer and current approvals have to date been granted in certain tumour types irrespective of PD-L1 status.

**Methods:** We extracted the following information: study sample size, trial period, cancer types, intervention of treatment, type of PD-L1 antibody, immunohistochemistry (IHC) scoring method, number and percentage of PD-L1 < 1% population, and median follow- up time. PD-L1 expression was defined as percentage of number of PD-L1-stained tumor cells (TPS), area of tumor infiltrated by PD-L1-stained immune cells (IPS), number of PD-L1-stained cells (tumor cells, lymphocytes and macrophages; CPS). Different trials used distinct method to define low PD-L1 expression. The risk of bias of the included trials was assessed by using the Cochrane risk of bias tool for RCTs.

**Results:** Here, a total of 34 trials were included to extract individual patient data (IPD) to evaluate the survival benefit of first line PD1/PDL1 inhibitors vs. standard-of-care (SOC) in patients with PDL1 < 1%. In term of anti-PD-1/PD-L1 monotherapy, OS (HR = 0.90, 0.81−1.01) and PFS (HR = 1.11, 0.97−1.27) between PD-1/PD-L1 inhibitor group and SOC group were comparable. In term of anti-PD-1/PD-L1 combination therapy, PD-1/PD-L1 inhibitor group exhibited longer OS (median 19.5 months vs. 16.3 months; HR = 0.83, 0.79−0.88, *p* < 0.001) and PFS than those of SOC group (median 8.11 months vs. 6.96 months; HR = 0.82, 0.77−0.87, *p* < 0.001).Subgroup analysis showed that survival benefit was mainly observed in non-small cell lung cancer (NSCLC) (HR_OS_ = 0.74; HR_PFS_ = 0.69; *p* < 0.001), small-cell lung cancer (SCLC) (HR_OS_ = 0.58, *p* < 0.001; HR_PFS_ = 0.55, *p* = 0.030), esophageal squamous cell carcinoma (ESCC) (HR_OS_ = 0.62, *p* = 0.005; HR_PFS_ = 0.79, *p* < 0.001), melanoma (HR_OS_ = 0.53, *p* < 0.001) and nasopharyngeal carcinoma (NPC) (HR_PFS_ = 0.35, *p* = 0.013).

**Conclusion:** Anti-PD-1/PD-L1 combinational therapy rather than monotherapy exhibit survival benefit in the low PD-L1 population in the first-line setting, and the survival benefit was mainly observed in specific tumor types.

## Introduction

Therapeutic blockade targeting programmed cell death 1 (PD-1) and its ligand (PD-L1) is one of the most important advances in the history of cancer treatment (Ribas and Wolchok, 2018). PD-1/PD-L1 inhibitors in the first-line setting, alone or in combination with other antitumor therapies, are increasingly being demonstrated to exhibit favorable long-term survival in many types of advanced-stage cancer, including melanoma, lung cancer, esophageal squamous cell carcinoma (ESCC), gastric carcinoma (GC) and many others (Doroshow et al., 2021).

Notably, recent randomized controlled trials (RCTs) of PD-1/PD-L1 inhibitors preferred to set the primary endpoints of survival in PD-L1-positive and intention-to-treat (ITT) populations (Rini et al., 2019; Emens et al., 2021; Miles et al., 2021). Most of these RCTs always published the data of ITT and PD-L1-positive populations, with a lack of presentation of the low PD-L1-expression subgroup.

CheckMate 648 showed that overall survival (OS) and progression-free survival (PFS) were significantly longer with nivolumab plus chemotherapy or ipilimumab than chemotherapy alone in all randomly assigned patients with ESCC, without reporting the Kaplan‒Meier (KM) curves for patients with absent or low PD-L1 expression (Doki et al., 2022). Similar observations were found in CheckMate 649 of GC (Janjigian et al., 2021) and CheckMate 743 of malignant pleural mesothelioma (MPM) (Peters et al., 2022). However, while two recent meta-analyses showed that PD1-/PD-L1 inhibitors failed to exhibit a survival benefit in the GC or ESCC patients with absent or low PD-L1 expression (Zhao et al., 2022a; Yap et al., 2023), the *post hoc* analysis of JUPITER-06 and meta-analysis showed superiority of PD-1 inhibitor with chemotherapy in advanced ESCC patients with absent or low PD-L1 expression. (Wu et al., 2022). Therefore, there are several critical and debatable issues: whether survival benefit in the randomized assigned population is largely derived from those in the PD-L1-positive population and whether PD-1/PD-L1 inhibitors can exhibit a survival benefit in patients with absent or low PD-L1 expression remain uncertain.

Here, we reconstructed individual patient data (IPD) of absent or low PD-L1 expression (PD-L1 < 1%) populations from the reported KM curves of high-quality RCTs, using a novel workflow, KMSubtraction (Zhao et al., 2022a; Zhao et al., 2022b; Yu et al., 2022). Given that the hazards in the trials of anti-PD-1/PD-L1 therapy are always not proportional during the entire study period, we used the approaches of log-rank test, Bayesian hierarchical model, and restricted mean survival time (RMST) to comprehensively evaluate the survival benefit of first line PD-1/PD-L1 inhibitor vs. standard-of-care (SOC) in patients with PD-L1 < 1%.

## Methods

This study was conducted following the Preferred Reporting Items for a Systematic Review and Meta-analysis of Individual Participant Data (PRISMA-IPD) protocol (Stewart et al., 2015).

### Data sources and selection

Two investigators (RCN and YBC) conducted independent literature searches of PubMed, Web of Science and Embase for eligible publications between 1 January 2015, and 8 February 2023, using the key words PD-1, PD-L1, checkpoint inhibitor, and phase 3 clinical trial (**eBox 1**).

Phase 3 RCTs were included if first line PD-1/PD-L1 inhibitors, alone or combined with other antitumor therapies (e.g., chemotherapy, targeted therapy or immunotherapy), were compared with SOC in patients with advanced tumors. The other criterion is that trials must report the hazard ratio (HR) of OS and/or PFS between PD-1/PD-L1 inhibitors and SOC in patients with low PD-L1 expression. We excluded reviews, conference abstracts and non-English-language articles. In the case of repeated studies reporting the same population, the most recent and most informative study was eligible.

### Data extraction

We extracted the following information: study sample size, trial period, cancer types, intervention of treatment, type of PD-L1 antibody, immunohistochemistry (IHC) scoring method, number and percentage of PD-L1 < 1% population, and median follow-up time. PD-L1 expression was defined as percentage of number of PD-L1-stained tumor cells (TPS), area of tumor infiltrated by PD-L1-stained immune cells (IPS), number of PD-L1-stained cells (tumor cells, lymphocytes and macrophages; CPS). Different trials used distinct method to define low PD-L1 expression. In this study, low PD-L1 expression was defined as TPS <1%, IPS <1%, TPS&IPS <1% or CPS <1. CPS = 10 can be equal to TPS = 1% (Wu et al., 2022; Yap et al., 2023). The risk of bias of the included trials was assessed by RCN and YBC using the Cochrane risk of bias tool for RCTs (Higgins, 2008).

## Reconstruction of time-to-event outcomes

For trials reporting KM curves of the PD-L1 < 1% population, IPD was extracted and decoded from the reported KM curves using the “IPDfromKM” package (Liu et al., 2021). The quality of reconstruction was evaluated by checking the at-risk tables, HRs, and shape of the KM curves.

For trials reporting KM curves of overall and PD-L1 ≥ 1% population, IPD was extracted using the “IPDfromKM” and “KMSubtraction” packages (Zhao et al., 2022b), which can derive unreported subgroup survival data from known subgroups. For KMSubtraction process, minimal-cost bipartite matching was adopted as the primary algorithm. Monte Carlo simulations with 1,000 iterations were used to evaluate the limits of error (Zhao et al., 2022b).

The quality of the reconstructed IPD was evaluated before the pooled analysis. Reconstruction KM curves of overall, subgroups with PD-L1 ≥ 1% and PD-L1 < 1% were compared with the original published KM curves, regarding the HRs, at-risk tables, and shape of the KM curves. In addition, we estimated the correlation between the reconstructed and reported outcomes using the Pearson correlation test.

An important aspect of validity is the representativeness of reconstructed IPD. To evaluate the representativeness of trials with available IPD, we performed standard meta-analysis models to combine aggregate data (from trials of non-IPD) with the available IPD. A random-effects model was used for this meta-analysis. Egger’s test and funnel plot analyses were assessed the presence of publication bias (Egger et al., 1997), with a two-tailed *p* < 0.05 considered statistically significant. Then, the HRs of trials with IPD and trials with total data (IPD and aggregate data) were compared.

### Simplified clinical benefit scale

The clinical benefit was graded by a simplified ESMO-Magnitude of Clinical Benefit Scale version 1.1 (Cherny et al., 2017; Korn et al., 2022) (Supplementary Table S1). In this study, grade 3/4 clinical benefit was considered meaningful. If the median OS for standard treatment was ≤12 months, the experimental arm median OS better by ≥ 2 months was considered clinically meaningful; if the median OS for standard treatment was >12 months and ≤24 months, the experimental arm median OS better by ≥ 3 months was considered meaningful. If median OS was not reached, a 10% increase in 2-year OS was considered meaningful. The upper limit of the 95% confidence interval (CI) for the HR should be less than 1.

### Primary endpoints

The primary outcomes were OS and PFS. OS was defined from the date of randomization to death from any cause. PFS was defined from the date of randomization to progressive disease as per RECIST guidelines (version 1.1) or death from any cause, whichever occurred first.

### One-stage pooled analysis

In this study, 1-stage approach was used to evaluate the survival benefit in subgroup of PD-L1 < 1%, through three different approaches. The primary analysis applied the log-rank test and marginal Cox model. To account for the between-study heterogeneity, the shared-frailty model was adopted to incorporate a random-effects terms, and the HRs were adjusted by the effect of cancer types, anti-PD-1/PD-L1 drugs, treatment of control arm. The subgroup analysis of different cancer types, anti-PD-1/PD-L1 drugs, PD-1/PD-L1 inhibitors (PD-1 or PD-L1), treatment regimens (single PD-1/PD-L1 regimen or combination PD-1/PD-L1 regimen), treatment of control arm, PD-L1 clone, and PD-L1 IHC scoring method was performed. In this study, we modified the predictive value of PD-L1 expression describe by Yoon et al. (Yoon et al., 2022), defined as log transformation of the ratio of HR of PDL1 < 1% *versus* ≥1% population.

We also applied a Bayesian hierarchical model with a time-varying hazard ratio (HR) (Berry, 2006). We modeled the time-varying HR effect by assuming that the hazards were constant within each 3-month follow-up and truncated the results at 60 months. Each 3-month segment had its own hazard rate and HR. The average HR adjusting the effect of cancer types, PD-1/PD-L1 agents, treatment of control arm was calculated. Markov chain Monte Carlo (MCMC) methods (1,000 iterations) (Gelman et al., 2013) were used to calculate the posterior mean of OS and PFS distributions and their corresponding 95% CI. The priors were set as default using the stan_surv function by rstanarm package. Rhat statistic was used to assess the convergence of the MCMC chains, with Rhat statistic less than 1.1 indicating the good evidence in favor of convergence (Carpenter et al., 2017).

The RMST was the nonparametric alternative strategy of the HR that does not rely on proportional hazards (Royston and Parmar, 2013). The RMST difference, the area bounded by 2 KM plots, represents the absolute gain or loss in survival. In this study, the truncation times were 2 years and 1 year for OS and PFS, respectively. If the minimum of the largest observed time in each of the two groups was shorter than 2 years for OS or 1 year for PFS, the truncation time was equal to this minimum of the largest observed time.

All statistical analyses were performed using R software, version 4.2.0 (http://www.r-project.org). *p* < 0.05 was considered statistically significant.

## Results

### Study selection and characteristics

Of 7,592 reports identified by the search strategy, 287 full-text articles met the eligibility criteria for detailed review. Of these, 49 phase 3 RCTs (Motzer et al., 2018; Paz-Ares et al., 2018; Socinski et al., 2018; Rini et al., 2019; West et al., 2019; Choueiri et al., 2020; Galsky et al., 2020; Jotte et al., 2020; Powles et al., 2020; Rudin et al., 2020; Motzer et al., 2021; Powles et al., 2021; Sun et al., 2021; Cheng et al., 2022; Wang et al., 2022; Cheng et al., 2022; Dummer et al., 2022; Gogishvili et al., 2022; Kang et al., 2022; Lu et al., 2022; Motzer et al., 2022; Shitara et al., 2022; Wu et al., 2022; Ascierto et al., 2023; de Castro et al., 2023; Johnson et al., 2023) involving 14,677 patients with PD-L1 < 1% met the inclusion criteria and were included (Supplementary Figure S1). The percentage of the PD-L1 < 1% population in each trial varied from 14.3% to 85.7% (Table 1).

**TABLE 1:** Characteristic of eligible studies.

Studies	Study number	Trial period	Population	Experimental arm	Control arm	PD-L1 antibody	IHC scoring method	Patients number		IPD or aggregate data	Median follow-up, m
								Low PD-L1 (%)	Total		
Paz-Ares et al. (2018)	KEYNOTE-407	2016–2017	Squamous NSCLC	Pembro + chemo	Placebo + chemo	22C3	TPS	194 (34.7)	559	IPD	7.8
Socinski et al. (2018), Socinski et al. (2021)	IMpower150	2015–2016	Nonsquamous NSCLC	Atezoli + bevacizumab + chemo	Bevacizumab + chemo	SP142	TPS&IPS	338 (42.2)	800	IPD	39.8
Motzer et al. (2018)	CheckMate 214	2014–2016	RCC	Nivo + ipi	Sunitinib	28–8	TPS	562 (67.0)	839	IPD	25.2
Rini et al. (2019)	IMmotion151	2015–2016	RCC	Atezoli + bevacizumab	Sunitinib	SP142	IPS	553 (60.4)	915	IPD	40.0
West et al. (2019)	IMpower130	2015–2017	Nonsquamous NSCLC	Atezoli + chemo	Chemo	SP142	TPS&IPS	356 (49.2)	723	aggregate data	18.5
Choueiri et al. (2020)	JAVELIN Renal 101	2015–2017	RCC	Avel + axitinib	Sunitinib	SP263	IPS	326 (36.8)	886	IPD	13.0
Galsky et al. (2020)	IMvigor130	2016–2018	UC	Atezoli + chemo Atezoli	Placebo + chemo	SP142	IPS	392 (32.3)	1,213	aggregate data	11.8
Gutzmer et al. (2020), Ascierto et al. (2023)	IMspire150	2017–2018	Melanoma	Atezoli + vemurafenib + cobimetinib	Placebo + vemurafenib + cobimetinib	SP142	IPS	171 (33.3)	514	aggregate data	18.9
Jotte et al. (2020)	IMpower131	2015–2017	Squamous NSCLC	Atezoli + chemo Atezoli	Chemo	SP142	TPS&IPS	331 (48.5)	683	aggregate data	26.8
Powles et al. (2020)	KEYNOTE-426	2016–2018	RCC	Pembro + axitinib	Sunitinib	22C3	CPS	321 (37.3)	861	aggregate data	30.6
Rudin et al. (2020)	KEYNOTE-604	2017–2018	SCLC	Pembro + chemo	Placebo + chemo	22C3	CPS	174 (38.4)	453	aggregate data	NR
Schmid et al. (2020), Emens et al. (2021)	IMpassion130	2015–2017	TNBC	Atezoli + chemo	Placebo + chemo	SP142	IPS	533 (61.3)	902	IPD	18.8
Janjigian et al. (2021), Shitara et al. (2022)	CheckMate 649	2017–2019	GC	Nivo + chemo Nivo + ipi	Chemo	28–8	CPS	342 (14.3)	2,394	IPD	13.1
Liu et al. (2021b)	IMpower133	2016–2017	ES-SCLC	Atezoli + chemo	Placebo + chemo	SP263	TPS&IPS	65 (16.2)	403	IPD	22.9
Luo et al. (2021)	ESCORT-1st	2018–2020	ESCC	Camre + chemo	Placebo + chemo	28–8	TPS	256 (43.0)	598	IPD	10.8
Mai et al. (2021)	NCT03581786	2018–2019	NPC	Toripal + chemo	Placebo + chemo	JS311	TPS&IPS	45 (15.6)	289	IPD	17.9
Miles et al. (2021) (Miles et al., 2021)	IMpassion131	2017–2019	TNBC	Atezoli + chemo	Placebo + chemo	SP142	IPS	359 (55.1)	651	IPD	15.2
Moehler et al. (2021)	JAVELIN Gastric 100	2015–2017	GC	Avel	Chemo	22C3	TPS	362 (72.5)	499	IPD	24.1
Monk et al. (2021)	JAVELIN Ovarian 100	2016–2018	OC	Chemo → Avel; Chemo + Avel→ Avel	Chemo → Obs	SP263	TPS&IPS	326 (32.7)	998	IPD	10.8
Moore et al. (2021)	IMagyn050/GOG 3015/ENGOT -OV39	2017–2019	OC	Atezoli + bevacizumab + chemo	Placebo + bevacizumab + chemo	SP142	IPS	517 (39.7)	1,301	IPD	19.9
Nishio et al. (2021)	IMpower132	2016–2017	Nonsquamous NSCLC	Atezoli + chemo	Chemo	SP142	TPS&IPS	163 (28.2)	578	IPD	14.8
Owonikoko et al. (2021)	CheckMate 451	2015–2018	ES-SCLC	Nivo + ipi; Nivo	Placebo	28–8	CPS	191 (22.9)	834	IPD	8.9
Reck et al. (2021)	CheckMate 9LA	2017–2019	NSCLC	Nivo + ipi + chemo	Chemo	28–8	TPS	264 (36.7)	719	IPD	30.7
Rodriguez-Abreu et al. (2021)	KEYNOTE-189	2016–2017	Nonsquamous NSCLC	Pembro + chemo	Placebo + chemo	22C3	TPS	190 (30.8)	616	IPD	31.0
Sugawara et al. (2021)	TASUKI-52	2017–2019	Nonsquamous NSCLC	Nivo + chemo	Placebo + chemo	28–8	TPS	240 (32.0)	750	IPD	13.7
Zhou et al. (2021)	CameL	2017–2018	Nonsquamous NSCLC	Camrel + chemo	Chemo	22C3	TPS	67 (16.7)	402	IPD	11.9
Motzer et al. (2021)	CLEAR	2016–2019	RCC	Pembro + Lenvatinib	Sunitinib	22C3	CPS	215 (35.1)	612	aggregate data	17.4
Powles et al. (2021)	KEYNOTE-361	2016–2018	UC	Pembro	Chemo	22C3	CPS*	341 (51.7)	659	aggregate data	31.7
Sun et al. (2021)	KEYNOTE-590	2017–2019	ESCC	Pembro + chemo	Placebo + chemo	22C3	CPS*	347 (46.3)	749	IPD	22.6
Burtness et al. (2022)	KEYNOTE-048	2015–2019	HNSCC	Pembro + chemo Pembro	Cetuximab + chemo	22C3	CPS	128 (14.5)	882	IPD	11.5
Cortes et al. (2022)	KEYNOTE-355	2017–2018	TNBC	Pembro + chemo	Placebo + chemo	22C3	CPS	211 (24.9)	847	IPD	44.1
Doki et al. (2022)	CheckMate 648	2017–2019	ESCC	Nivo + chemo Nivo + ipi	Chemo	28–8	TPS	497 (51.2)	970	IPD	13.0
Paz-Ares et al. (2022)	CheckMate 227	2015–2016	NSCLC	Nivo + ipi Nivo + chemo	Chemo	28–8	TPS	550 (31.6)	1739	IPD	54.8
Baas et al. (2021)	CheckMate 743	2016–2018	MPM	Nivo + ipi	Chemo	28–8	TPS	135 (22.3)	605	IPD	43.1
Spigel et al. (2022)	PACIFIC	2017–2021	NSCLC	CRT followed by durva	CRT followed by placebo	SP263	TPS	148 (20.8)	713	IPD	34.2
Wolchok et al. (2022)	CheckMate 067	2013–2014	Melanoma	Nivo + ipi Nivo	Ipi	28–8	TPS	353 (35.4)	945	IPD	77.0
Yau et al. (2022)	CheckMate 459	2016–2017	HCC	Nivo	Sorafenib	28–8	TPS	595 (80.1)	743	IPD	15.2
Zhou et al. (2022)	GEMSTONE-302	2018–2020	NSCLC	Suge + chemo	Placebo + chemo	SP263	TPS	188 (39.2)	479	IPD	8.6
Cheng et al. (2022a)	IMbrave150	2018–2019	HCC	Atezoli + bevacizumab	Sorafenib	SP142	TPS&IPS	77 (15.3)	503	aggregate data	15.6
Gogishvili et al. (2022)	EMPOWER-Lung 3	2019–2020	NSCLC	Cemip + chemo	Placebo + chemo	SP263	TPS	139 (29.8)	466	aggregate data	16.3
Kang et al. (2022)	ATTRACTION-4	2017–2018	GC	Nivo + chemo	Placebo + chemo	28–8	TPS	610 (84.2)	724	aggregate data	26.6
Lu et al. (2022)	ORIENT-15	2018–2021	ESCC	Sinti + chemo	Placebo + chemo	22C3	TPS	297 (45.1)	659	IPD	16.0
Motzer et al. (2022)	CheckMate 9ER	2017–2019	RCC	Nivo + cabozantinib	Sunitinib	28–8	TPS	472 (72.5)	651	aggregate data	32.9
Wang et al. (2022a)	CAPSTONE-1	2018–2020	ES-SCLC	Adebre + chemo	Placebo + chemo	22C3	TPS	396 (85.7)	462	aggregate data	13.5
Cheng et al. (2022b)	ASTRUM-005	2019–2021	ES-SCLC	Serplu + chemo	Placebo + chemo	22C3	TPS	469 (81.2)	585	aggregate data	12.5
Johnson et al. (2023)	POSEIDON	2017–2018	NSCLC	Durva + treme + chemo Durva + chemo	Chemo	SP263	TPS	368 (36.3)	1,013	aggregate data	34.9
Dummer et al. (2022)	COMBI-i	2017–2018	Melanoma	Sparta + dabrafenib + trametinib	Placebo + dabrafenib + trametinib	28–8	TPS	213 (40.0)	532	IPD and aggregate data	27.2
Wang et al. (2022b), Wu et al. (2022)	JUPITER-06	2019–2020	ESCC	Toripal + chemo	Placebo + chemo	JS311	TPS	193 (65.4)	295	IPD	7.1
de Castro et al. (2023)	NEPTUNE	NR	NSCLC	Durva + treme	Chemo	SP263	IPS	195 (23.7)	823	IPD	32.9

These studies covered 28 trials with anti-PD-1 (including 12 with nivolumab, 9 with pembrolizumab, 2 with camrelizumab, 2 with toripalimab, 1 with sintilimab, 1 with serplulimab, and 1 with spartalizumab) and 21 trials with anti-PD-L1 (including 12 with atezolizumab, 3 with avelumab, 3 with durvalumab, 1 with adebrelimab, 1 with cemiplimab, and 1 with sugemalimab) agents. Fifteen trials were conducted in patients with non-small cell lung cancer (NSCLC), six trials in patients with renal cell carcinoma (RCC), five trials in patients with small-cell lung cancer (SCLC), five trials in patients with ESCC, three trials in patients with triple-negative breast cancer (TNBC), three trials in patients with GC, three trial in patients with melanoma, two trials in patients with ovarian cancer (OC), two trial in patients with hepatocellular carcinoma (HCC), two trials in patients with urothelial cancer (UC),1 trial in patients with head and neck squamous cell carcinoma (HNSCC), one trial in patients with MPM, and one trial in patients with nasopharyngeal carcinoma (NPC). Of the 49 trials, fourteen trials assessed PD-L1 expression with the use of IHC antibody 28–8, 14 trials with 22C3, 11 trials with SP142, eight trials with SP263, and two trial with JS311. In terms of the PD-L1 IHC scoring method, 24 trials defined PD-L1 expression with the use of the TPS, eight trials with the IPS, eight trials with TPS&IPS, and nine trials with the CPS.

Among the 49 trials, 34 trials were available for the IPD and 15 trials were available for the aggregate data, with a total of 52 comparisons of OS and 49 comparisons of PFS. A total of 13 comparisons of single anti-PD-1/PD-L1 agents (OS: 8; PFS: 5), 88 comparisons of combination anti-PD-1/PD-L1 agents (OS: 44; PFS: 44) were included (Table 1). Table 1 provides further information on the study characteristics.

### Meta-analysis of IPD and aggregate data

The quality of most included trials was generally high (Supplementary Table S2), and no publication bias was observed (Supplementary Figure S2) in the IPD and aggregate data. The random-effect model was used to evaluate the pooled effect of OS and PFS. We obtained a pooled HR of 0.82 (0.77–0.87) for OS. Of note, we found that patients with PD-L1 < 1% can benefit from combination PD-1/PD-L1 regimens (HR 0.80, 0.75–0.86) rather than single PD-1/PD-L1 regimens (HR 0.93, 0.81–1.07). The subgroup difference was significant (*p* = 0.049) (Supplementary Figure S3). Similar results were found regarding PFS (Supplementary Figure S4).

### Representativeness and quality of reconstructed IPD

Before the reconstruction of IPD, the HRs of trials with IPD and trials with total data (IPD and aggregate data) were compared. Of note, the HRs of trials with IPD and trials with total data were comparable (Supplementary Figure S5), indicating that treatment effect estimated by trials with IPD can effectively represent those by total data.

We summarized the extraction process of IPD of PD-L1 < 1% in Supplementary Tables S3-S4. The reconstructed KM curves of overall, subgroups with PD-L1 ≥ 1% and PD-L1 < 1% were similar to those of original curves (Supplementary Table S4), and the limits of error of KMSubtraction of extraction of unreported subgroup were small and negligible (Supplementary Figure S6). Then, we calculated the correlation between the reconstructed outcomes and reported outcomes from the original articles. As expected, we observed extremely strong associations in terms of HR, median survival time, OS rate and PFS rate (all Pearson correlation coefficients >0.99 and all *p* < 0.001, Supplementary Figure S7), indicating that the reconstructed IPD could effectively represent the original data.

### Survival analysis: log-rank test

**FIGURE 1:**
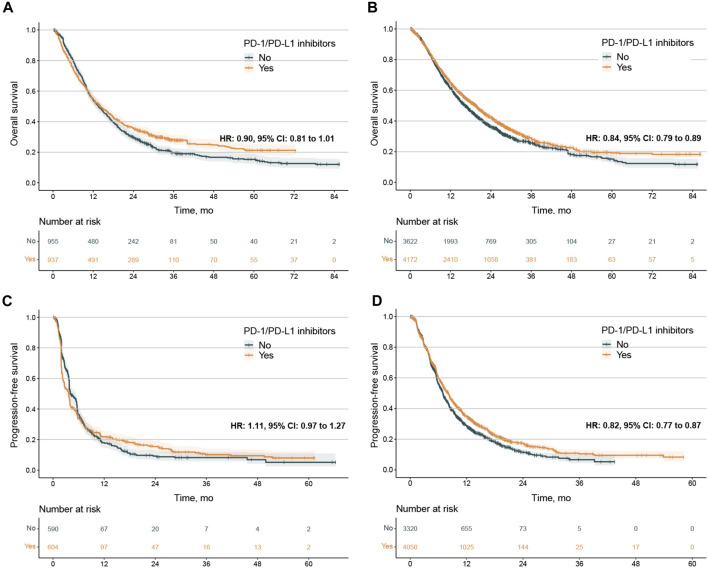
Kaplan‒Meier estimates of overall survival and progression-free survival. **(A, B)**, overall survival (OS) in the PD-L1 < 1% population treated with PD-1/PD-L1 single agents **(A)** and combination agents **(B)**. C-D, progression-free survival (PFS) In the PD-L1 < 1% population treated with PD-1/PD-L1 single agents **(C)** and combination agents **(D)**. The 2-year OS was 35.6% (32.6%–38.9%) vs. 29.6% (26.7%–32.8%) for patients treated PD-1/PD-L1 single agents or not, 43.2% (41.5%–44.9%) vs. 37.2% (35.4%–39.0%) or patients treated PD-1/PD-L1 combination agents or not. The 1-year PFS was 21.9% (18.6%–25.8%) vs. 18.0% (14.7%–21.9%) for patients treated PD-1/PD-L1 single agents or not, 35.5% (33.8%-37.2) vs. 29.4% (27.7%–31.3%) or patients treated PD-1/PD-L1 combination agents or not. PD-1, programmed death-1; PD-L1, programmed death-ligand 1; HR, hazard ratio; CI, confidence interval.

**FIGURE 2:**
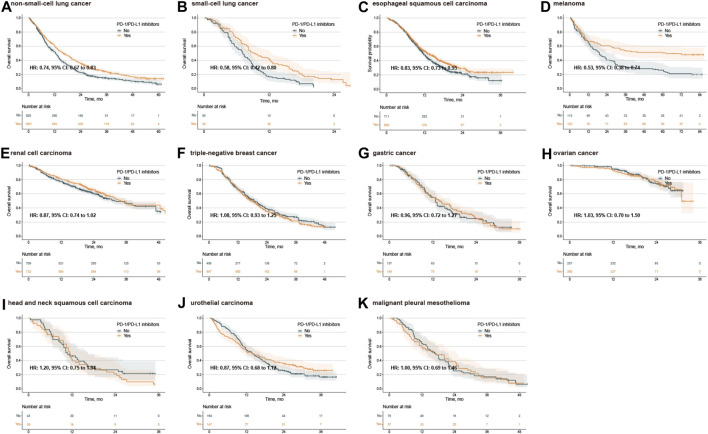
Kaplan‒Meier estimates of overall survival treated with combination agents, stratified by cancer type. Overall survival in the PD-L1 < 1% population with non-small-cell lung cancer **(A)**, small-cell lung cancer **(B)**, esophageal squamous cell carcinoma **(C)**, melanoma **(D)**, renal cell carcinoma **(E)**, triple-negative breast cancer **(F)**, gastric cancer **(G)**, ovarian cancer **(H)**, head and neck squamous cell carcinoma **(I)**, urothelial carcinoma **(J)**, and malignant pleural mesothelioma **(K)**. PD-1, programmed death-1; PD-L1, programmed death-ligand 1; HR, hazard ratio; CI, confidence interval.

No publication bias was observed in the IPD analysis (Supplementary Figure S8). Next, we conducted survival analysis using the log-rank test, stratified by single/combination PD-1/PD-L1 inhibitors. The IPD of OS from 33 trials were available for 9,686 patients. In the analysis for OS of single PD-1/PD-L1 inhibitors, the median OS was 14.1 months (12.5–16.2) in the PD-1/PD-L1 inhibitor group and 13.6 months (12.5–15.0) in the SOC group (adjusted HR 0.90, 0.81–1.01, *p* = 0.063) (Figure 1A), with no clinical benefit. Subgroup analysis stratified by cancer types further showed no statistically significant difference for single PD-1/PD-L1 inhibitors compared with SOC (Supplementary Figure S9). In the analysis for OS of combination PD-1/PD-L1 inhibitors, the median OS was 19.5 months (18.5–20.1) in the PD-1/PD-L1 inhibitor group and 16.3 months (15.5–17.2) in the SOC group (adjusted HR 0.83, 0.79–0.88, *p* < 0.001) (Figure 1B), with grade 3 clinical benefit. Then, we explored the subgroup of OS in patients treated with combination PD-1/PD-L1 inhibitors, and found that the PD-1/PD-L1 inhibitor group only showed OS benefit in patients with NSCLC, SCLC, ESCC and melanoma (Figure 2; Supplementary Figure S10). Interestingly, we found that PD-1 inhibitors rather than PD-L1 inhibitors showed OS benefit (*p* = 0.001 for subgroup difference; Supplementary Figure S10; Supplementary Figure S11A, B). Of note, if TPS or TPS&IPS was used to assess PD-L1 expression, PD-1/PD-L1 inhibitors exhibited OS benefit and clinical benefit in patients with PD-L1 < 1% (Supplementary Figure S10).

**FIGURE 3:**
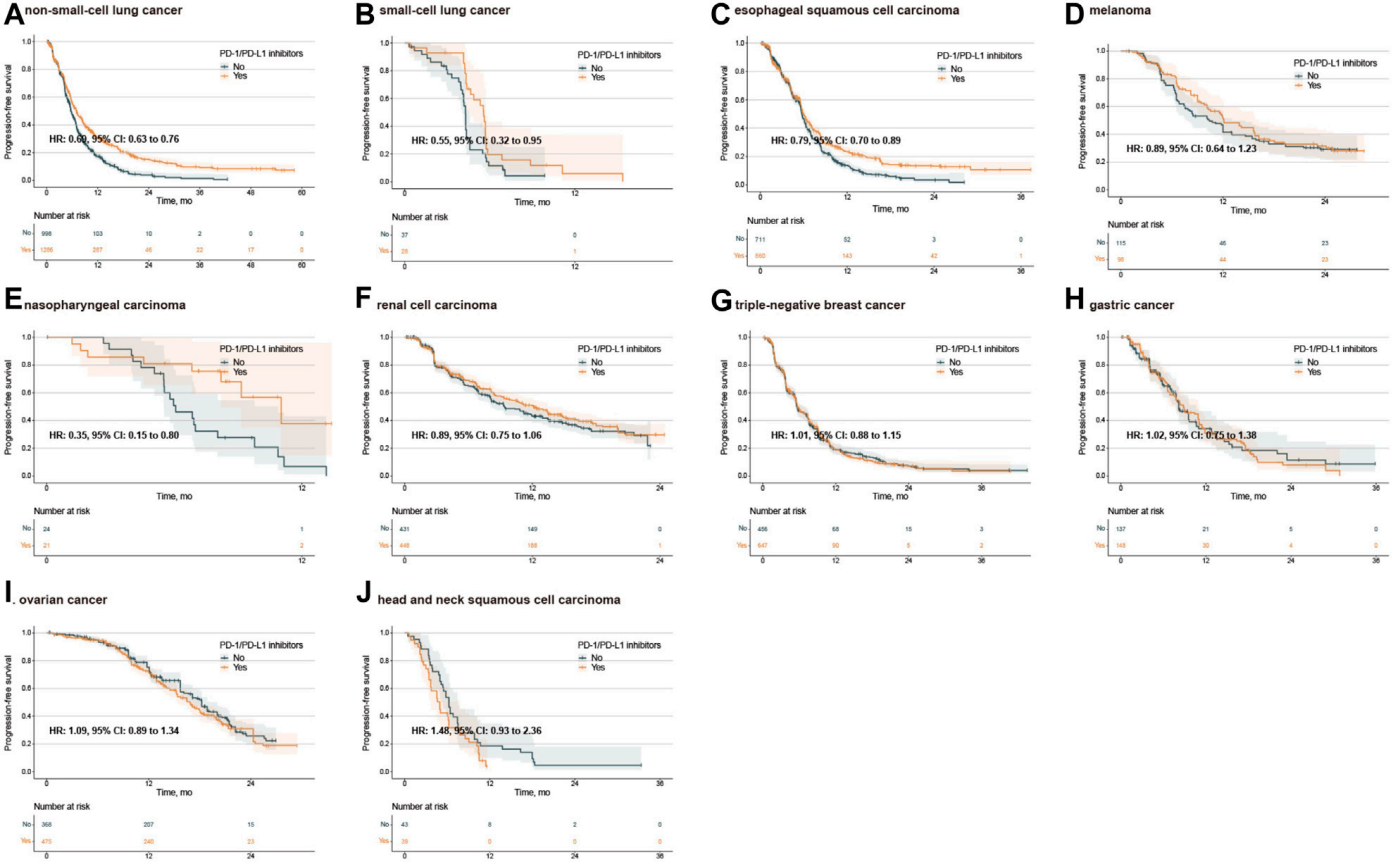
Kaplan‒Meier estimates of progression-free survival treated with combination agents, stratified by cancer type. Progression-free survival in the PD-L1 < 1% population with non-small-cell lung cancer **(A)**, small-cell lung cancer **(B)**, esophageal squamous cell carcinoma **(C)**, melanoma **(D)**, nasopharyngeal carcinoma **(E)**, renal cell carcinoma **(F)**, triple-negative breast cancer **(G)**, gastric cancer **(H)**, ovarian cancer **(I)**, and head and neck squamous cell carcinoma **(J)**. PD-1, programmed death-1; PD-L1, programmed death-ligand 1; HR, hazard ratio; CI, confidence interval.

IPD of PFS from 33 trials were available for 8,217 patients. In the PFS analysis of single PD-1/PD-L1 inhibitors, the PFS between PD-1/PD-L1 inhibitor group and SOC group were comparable (median 3.68 months, 2.95 to 3.91 vs. 4.05 months, 3.81 to 5.40; adjusted HR 1.11, 0.97 to 1.27, *p* = 0.122) (Figure 1C). Subgroup analysis stratified by cancer types further showed no PFS difference for single PD-1/PD-L1 inhibitors compared with SOC (Supplementary Figure S12). In the analysis for PFS of combination PD-1/PD-L1 inhibitors, PD-1/PD-L1 inhibitor group exhibited longer PFS than those of SOC group (median 8.11 months, 7.58 to 8.33 vs.6.96 months, 6.78 to 7.11; adjusted HR 0.82, 0.77 to 0.87, *p* < 0.001) (Figure 1D). Then, we explored the subgroup of PFS in patients treated with combination PD-1/PD-L1 inhibitors. The PD-1/PD-L1 inhibitor group showed a PFS benefit in patients with NSCLC, SCLC, ESCC and NPC (Figure 3; Supplementary Figure S13). Similarly, treatment effect of PD-1 inhibitors was higher than those of PD-L1 inhibitors (*p* = 0.111 for subgroup difference; Supplementary Figure S11C, D; Supplementary Figure S13), and if TPS or TPS&IPS was used to assess PD-L1 expression, PD-1/PD-L1 inhibitors exhibited a PFS benefit in patients with PD-L1 < 1% (Supplementary Figure S13B).

### Survival analysis: bayesian hierarchical approach

We next conducted survival analysis using a Bayesian hierarchical model. The survival curves for the PD-1/PD-L1 inhibitor and SOC groups are shown in Supplementary Figure S14. In term of single PD-1/PD-L1 inhibitors, both OS and PFS were similar between patients treated with PD-1/PD-L1 inhibitors and those treated with SOC, regardless of cancer types (Supplementary Figure S14A, C; Supplementary Figure S15, 16). In term of combination PD-1/PD-L1 inhibitors, PD-1/PD-L1 inhibitor group exhibited OS and PFS benefit. At 2 years, the estimated OS was 44.4% (43.0%–45.9%) for the combination PD-1/PD-L1 inhibitor group and 39.2% (37.8%–40.8%) for the SOC group. The average adjusted HR for OS was 0.83 (0.78–0.88) (Supplementary Figure S14B). At 1 year, the estimated PFS was 40.0% (38.7%–41.3%) for the combination PD-1/PD-L1 inhibitor group and 33.4% (31.8%–34.9%) for the SOC group. The average adjusted HR for PFS was 0.79 (0.74–0.82) (Supplementary Figure S14D). Subgroup analysis stratified by cancer types also demonstrated similar results to those of the log-rank test (Supplementary Figure SS17, 18).

### Survival analysis: RMST test

The difference in RMST between the PD-1/PD-L1 inhibitor and SOC groups was further estimated. In term of single PD-1/PD-L1 inhibitors, the RMST difference between the two groups failed to exhibit statistical significance (Supplementary Figure S19, 20). In term of combination PD-1/PD-L1 inhibitors, the RMST difference between the two groups started to exhibit statistical significance at truncation time points of13 months for OS and 8 months for PFS (Supplementary Figure S19). The 2-year RMST difference between the two groups was 0.79 months (0.41–1.16) for OS, and the 1-year RMST difference between the two groups was 0.40 months (0.21–0.59) for PFS. Notably, we observed that only seven trials showed a significant 2-year RMST difference for OS, and nine trials showed a significant 1-year RMST difference for PFS (Supplementary Figure S21).

### Predictive value of PD-L1 expression

Finally, we estimated the predictive value of PD-L1 expression. PD-L1 expression ranged from −0.41 to 0.67 (Supplementary Table S5; Supplementary Figure S22), and −0.52 to 0.94 for each subgroup (Supplementary Figure S23).

## Discussion

To our knowledge, this is the largest IPD meta-analysis that investigates the survival benefit of first-line anti-PD-1/PD-L1 therapy in patients with PD-L1 < 1%. The results suggest that anti-PD-1/PD-L1 monotherapy failed to exhibit survival benefit in the low PD-L1 population. The magnitude of the survival benefit associated with anti-PD-1/PD-L1 combinational therapy in the low PD-L1 population was moderate (grade 3 clinical benefit). In addition, a survival benefit of anti-PD-1/PD-L1 combinational therapy was mainly observed in specific tumor types, including NSCLC, SCLC, ESCC, melanoma and NPC.

Recently, there have been an increasing number of RCTs demonstrating the survival benefit of PD-1/PD-L1 inhibitors for the treatment of patients with late-stage tumors in the first-line setting, accelerating regulatory approval by the FDA. These approvals have promoted the exploration of the efficacy of PD-1/PD-L1 inhibitors in earlier-stage settings (Ascierto et al., 2020; Forde et al., 2022; Schmid et al., 2022). A previous meta-analysis reported that PD-1/PD-L1 inhibitors prolonged the survival in patients with PD-L1 negative in the second and later line setting (Shen and Zhao, 2018). However, this issue in the first line setting is datable. In terms of mechanism, PD-L1 expressed on tumor cells promotes immune evasion (Topalian et al., 2015; Sanmamed and Chen, 2018), and therapeutic blockade of the PD-1 pathway theoretically requires the expression of PD-L1 on antigen-presenting cells and tumor cells (Yamaguchi et al., 2022).

In the present study, we noted that the proportion of the PD-L1 < 1% population was high (39.6%, 14,677/37,036; range: 14.3%–85.7%), which warrants a deeper analysis to identify whether the absent or low PD-L1 population can truly benefit from PD-1/PD-L1 inhibitors. We utilized a novel approach (KMSubtraction) to extract the unreported subgroups of IPD of the PD-L1 < 1% population from 34 high-quality phase 3 RCTs. The reconstructed IPD were representative. Overall, our findings suggested that the use of PD-1/PD-L1 inhibitors alone in the first line setting failed to provide OS or PFS benefit in patients with absent or low PD-L1 expression compared with SOC, which suggested the importance of PD-L1 expression in PD-1/PD-L1 blockade therapy. Anti-PD-1/PD-L1 combinational therapy exhibited OS and PFS benefit in the low PD-L1 population, which can be explained that chemotherapy and targeted therapy can induce PD-L1 expression (Akbay et al., 2013; Parra et al., 2018). Nonetheless, we also found that the timepoint at which PD-1/PD-L1 inhibitors initially exhibited a survival benefit was lagging (13 months for OS and 8 months for PFS). In addition, most of the eligible trials and subgroups appeared to have a positive predictive value for PD-L1 expression, consistent with a previous study (Yoon et al., 2022). Together, these results suggested that most patients with absent or low PD-L1 expression should not be indicated for PD-1/PD-L1 inhibitors.

The large IPD of this study allowed for relevant subgroup analyses. The efficacy of PD-1/PD-L1 inhibitors may differ across cancer types (Morad et al., 2021). Therefore, we first assessed the efficacy of PD-1/PD-L1 inhibitors in different cancer types. A total of 11 cancer types were included. The efficacy of PD-1/PD-L1 inhibitors in advanced ESCC with low PD-L1 expression was debatable (Wu et al., 2022; Yap et al., 2023). In this study, we extracted IPD from five trials (CheckMate 648 ^6^, ESCORT-first (Luo et al., 2021), JUPITER-06 ^11^, KEYNOTE-590 ^49^, ORIENT-15 ^29^), and found that advanced ESCC patients with low PD-L1 expression can still benefit from anti-PD-1/PD-L1 combinational therapy. The efficacy of PD-1/PD-L1 inhibitors in other cancer types were investigated. Overall, PD-1/PD-L1 inhibitors did not show a survival benefit in most cancer types but were associated with a modestly improved survival benefit in patients with NSCLC, SCLC, melanoma and NPC. The treatment effect of anti-PD-1 and anti-PD-L1 therapy may be different. Our findings indicated that the treatment effect of anti-PD-1 therapy in the first-line setting may be stronger than those of anti-PD-L1 therapy, consistent with previous study (Duan et al., 2020).

In clinical practice, IHC is the most common technology to quantify PD-L1 expression on tumor cells and tumor-infiltrating immune cells (Doroshow et al., 2021). RCTs in which patients receive different PD-1/PD-L1 inhibitors often used different PD-L1 IHC assays. Of note, when the 28–8 assay was used to identify the status of PD-L1 < 1%, PD-1/PD-L1 inhibitors still showed OS and PFS benefits. Interestingly, the predictive values of PD-L1 expression diagnosed by the 28–8 assay were 0.12 for OS and 0.10 for PFS, which indicated that PD-L1 expression diagnosed by the 28–8 assay is a biomarker to predict the intensity of efficacy of PD-1/PD-L1 inhibitors but not a biomarker to select patients who should receive anti-PD-1/PD-L1 therapy.

The IHC scoring algorithm involved the evaluation of TPS, IPS, or CPS (Doroshow et al., 2021). Notably, we found that if CPS was used to assess PD-L1 expression, PD-1/PD-L1 inhibitors exhibited OS (HR 0.75, 0.68–0.83) and PFS (HR 0.77, 0.60–0.99) benefits in patients with PD-L1 ≥ 1% but no OS (HR 0.91, 0.76–1.09) and PFS (HR 1.35, 0.76–2.39) benefits in patients with PD-L1 < 1%. Furthermore, the predictive value of CPS-based PD-L1 expression was the highest (0.19 for OS and 0.56 for PFS) among the IHC scoring algorithms, suggesting that CPS at a cutoff point of 1 may be powerful for selecting patients for anti-PD-1/PD-L1 immunotherapy. Nevertheless, it should be noted that the intraclass correlation coefficients for a CPS of ≥1 were relatively low (0.39 and 0.26 using the 22C3 and SP263 assays, respectively) (Park et al., 2020). Therefore, further research is warranted.

### Strengths and limitations

Most importantly, this study is the largest IPD meta-analysis of this topic. In addition, rather than extracting and pooling the study-level HR estimates, we applied an advanced method to reconstruct the IPD from published KM curves, which enables more elaborate survival analysis. The reconstructed IPD were validated through elaborate analysis, and can reflect the original data and represent the non-IPD trials. In addition to the log-rank test, we applied a Bayesian hierarchical model and RMST analysis to integrate survival data, which can overcome the potential limitations of proportional hazards modeling.

This study also has several notable limitations. These in any such meta-analysis include the potential for publication bias that not all the RCTs in the first-line setting report the KM plots of PD-L1 < 1% population or total and PD-L1 ≥ 1% population. Second, the PD-L1 expression of different tissue (primary *versus* metastatic samples) and intratumoral position (different spatiotemporal part of the same samples) may be different. Third, although we adopted random-effects model and calculated HRs adjusting other covariates, some between-study heterogeneities were still inevitable, such as different criterion to define PD-L1 < 1% (TPS, IPS or CPS; different PD-L1 clones). Fourth, PD-L1 expression might change after receiving another therapy, one-timepoint assessment rather than dynamic records of PD-L1 is inadequate. Fifth, although we performed methodological precautions to ensure the reconstructed KM curves and HRs for low PD-L1 expression subgroups are close to the reported data, we acknowledged some minute differences. Finally, the safety data were unavailable in this study.

## Conclusion

Compared with SOC, anti-PD-1/PD-L1 monotherapy failed to exhibit survival benefit in the low PD-L1 population in the first-line setting. The magnitude of the survival benefit associated with anti-PD-1/PD-L1 combinational therapy in the low PD-L1 population was moderate, and the survival benefit was mainly observed in specific tumor types, including NSCLC, SCLC, ESCC, melanoma and NPC.

## Figures and Tables

**Table:** 

**PD-1**	programmed cell death 1
**PD-L1**	programmed cell death ligand 1
**ESCC**	esophageal squamous cell carcinoma
**GC**	gastric cancer
**RCT**	randomized controlled trial
**ITT**	intention-to-treat
**FDA**	Food and Drug Administration
**OS**	overall survival
**PFS**	progression-free survival
**KM**	Kaplan‒Meier
**MPM**	malignant pleural mesothelioma
**IPD**	individual patient data
**RMST**	restricted mean survival time
**SOC**	standard-of-care
**HR**	hazard ratio
**IHC**	immunohistochemistry
**TPS**	tumor proportion score
**IPS**	immune proportion score
**CPS**	combined proportion score
**CI**	confidence interval
**MCMC**	Markov chain Monte Carlo
**NSCLC**	non-small cell lung cancer
**RCC**	renal cell carcinoma
**SCLC**	small-cell lung cancer
**TNBC**	triple-negative breast cancer
**OC**	ovarian cancer
**HCC**	hepatocellular carcinoma
**UC**	urothelial cancer
**HNSCC**	head and neck squamous cell carcinoma
**NPC**	nasopharyngeal carcinoma
**NSCLC**	non-small-cell lung cancer
**RCC**	renal cell carcinoma
**UC**	urothelial cancer
**TNBC**	triple-negative breast cancer
**ES-SCLC**	extensive-stage small-cell lung cancer
**ESCC**	esophageal squamous cell carcinoma
**GC**	gastric cancer
**HNSCC**	head and neck squamous cell carcinoma
**OC**	ovarian cancer
**NPC**	nasopharyngeal carcinoma
**MPM**	malignant pleural mesothelioma
**HCC**	hepatocellular carcinoma
**UC**	urothelial carcinoma
**IHC**	immunohistochemical
**TPS**	tumor cell proportion score
**CPS**	combined positive score
**IPS**	immune cell proportion score
**Nivo**	Nivolumab
**Pembro**	Pembrolizumab
**Atezoli**	Atezolizumab
**Avel**	Avelumab
**Durva**	Durvalumab
**Ipi**	Ipilimumab
**Camre**	Camrelizumab
**Toripal**	Toripalimab
**Suge**	Sugemalimab
**Sinti**	Sintilimab
**Cemip**	cemiplimab
**Adebre**	adebrelimab
**Serplu**	serplulimab
**Treme**	tremelimumab
**Sparta**	spartalizumab
**Obs**	observation
**Chemo**	chemotherapy
**PD-L1**	programmed death-ligand 1
**NR**	not reported

## Data Availability

The original contributions presented in the study are included in the article/Supplementary Material, further inquiries can be directed to the corresponding authors.
