# Supplementary material for: First-line immune checkpoint inhibitors in low programmed death-ligand 1-expressing population
Source: Front Pharmacol. 2024 Jul 26;15:1377690. doi: 10.3389/fphar.2024.1377690 (PMC11310016; doi:10.3389/fphar.2024.1377690)
Supplement: Supplementary file 1 [file DataSheet1.pdf]

eBox 1 Literature search strategy

A. PubMed: (n=2882)

#1 Nivolumab[tiab]
#2 Opdivo[tiab]
#3 BMS-936558 [tiab]
#4 MDX1106 [tiab]
#5 ONO-4538 [tiab]
#6 BMS-936559 [tiab]
#7 MDX1105 [tiab]
#8 Pembrolizumab[tiab]
#9 Lambrolizumab [tiab]
#10 Keytruda [tiab]
#11 MK-3475 [tiab]
#12 Atezolizumab [tiab]
#13 MPDL3280 [tiab]
#14 MPDL3280A [tiab]
#15 RG7446 [tiab]
#16 Tecentriq [tiab]
#17 Avelumab [tiab]
#18 MSB0010718C [tiab]
#19 Durvalumab [tiab]
#20 MEDI4736 [tiab]
#21 Cemiplimab [tiab]
#22 REGN2810 [tiab]
#23 PD-1 inhibitor [tiab]
#24 Programmed death 1 inhibitor [tiab]
#25 Anti-PD-1 [tiab]
#26 Anti-Programmed Cell Death 1 [tiab]
#27 PD-L1 inhibitor [tiab]
#28 Programmed death ligand 1 inhibitor [tiab]
#29 Anti-PD-L1 [tiab]
#30 Anti-Programmed Cell Death Ligand-1[tiab]
#31 Checkpoint Inhibitor [tiab]
#32 Checkpoint blockade [tiab]
#33 Programmed Cell Death 1 Receptor [tiab]
#34 Programmed Cell Death 1 Receptor [mesh]
#35 #1 OR #2 OR #3 OR #4 OR #5 OR #6 OR #7 OR #8 OR #9 OR #10 OR #11 OR #12 OR #13 OR #14 OR #15 OR #16 OR #17 OR #18 OR #19 OR #20 OR #21 OR #22 OR #23 OR #24 OR #25 OR #26 OR #27 OR #28 OR #29 OR #30 OR #31 OR #32 OR #33 OR #34
#36 randomized controlled trial [pt]
#37 controlled clinical trial [pt]
#38 randomized [tiab]
#39 phase 3 [tiab]
#40 phase III [tiab]
#41 #36 OR #37 OR #38 OR #39 OR #40
#42 #35 and #41

B. EMBASE (n=2032)

#1 Nivolumab: ab,ti
#2 Opdivo: ab,ti
#3 BMS-936558: ab,ti
#4 MDX1106: ab,ti
#5 ONO-4538: ab,ti
#6 BMS-936559: ab,ti
#7 MDX1105: ab,ti
#8 Pembrolizumab: ab,ti
#9 Lambrolizumab: ab,ti
#10 Keytruda: ab,ti
#11 MK-3475: ab,ti
#12 Atezolizumab: ab,ti
#13 MPDL3280: ab,ti
#14 MPDL3280A: ab,ti
#15 RG7446: ab,ti
#16 Tecentriq: ab,ti
#17 Avelumab: ab,ti
#18 MSB0010718C: ab,ti
#19 Durvalumab: ab,ti
#20 MEDI4736: ab,ti
#21 Cemiplimab: ab,ti
#22 REGN2810: ab,ti
#23 PD-1 inhibitor: ab,ti
#24 Programmed death 1 inhibitor: ab,ti
#25 Anti-PD-1: ab,ti
#26 Anti-Programmed Cell Death 1: ab,ti
#27 PD-L1 inhibitor: ab,ti
#28 Programmed death ligand 1 inhibitor: ab,ti
#29 Anti-PD-L1: ab,ti
#30 Anti-Programmed Cell Death Ligand-1: ab,ti
#31 Checkpoint Inhibitor: ab,ti
#32 Checkpoint blockade: ab,ti
#33 Programmed Cell Death 1 Receptor: ab,ti
#34 #1 OR #2 OR #3 OR #4 OR #5 OR #6 OR #7 OR #8 OR #9 OR #10 OR #11 OR #12 OR #13 OR #14 OR #15 OR #16 OR #17 OR #18 OR #19 OR #20 OR #21 OR #22 OR #23 OR #24 OR #25 OR #26 OR #27 OR #28 OR #29 OR #30 OR #31 OR #32 OR #33
#35 randomized controlled trial: ab,ti
#36 controlled clinical trial: ab,ti
#37 randomized: ab,ti
#38 phase 3: ab,ti
#39 phase III: ab,ti
#40 #35 OR #36 OR #37 OR #38 OR #39
#41 #34 AND #40 AND 'human'/de AND ('article'/it OR 'article in press'/it)

C. Web of Science (n=2678)

#1 Nivolumab: Topic
#2 Opdivo: Topic
#3 BMS-936558: Topic

#4 MDX1106: Topic
#5 ONO-4538: Topic
#6 BMS-936559: Topic
#7 MDX1105: Topic
#8 Pembrolizumab: Topic
#9 Lambrolizumab: Topic
#10 Keytruda: Topic
#11 MK-3475: Topic
#12 Atezolizumab: Topic
#13 MPDL3280: Topic
#14 MPDL3280A: Topic
#15 RG7446: Topic
#16 Tecentriq: Topic
#17 Avelumab: Topic
#18 MSB0010718C: Topic
#19 Durvalumab: Topic
#20 MEDI4736: Topic
#21 Cemiplimab: Topic
#22 REGN2810: Topic
#23 PD-1 inhibitor: Topic
#24 Programmed death 1 inhibitor: Topic
#25 Anti-PD-1: Topic
#26 Anti-Programmed Cell Death 1: Topic
#27 PD-L1 inhibitor: Topic
#28 Programmed death ligand 1 inhibitor: Topic
#29 Anti-PD-L1: Topic
#30 Anti-Programmed Cell Death Ligand-1: Topic
#31 Checkpoint Inhibitor: Topic
#32 Checkpoint blockade: Topic
#33 Programmed Cell Death 1 Receptor: Topic
#34 #1 OR #2 OR #3 OR #4 OR #5 OR #6 OR #7 OR #8 OR #9 OR #10 OR #11 OR #12 OR #13 OR #14 OR #15 OR #16 OR #17 OR #18 OR #19 OR #20 OR #21 OR #22 OR #23 OR #24 OR #25 OR #26 OR #27 OR #28 OR #29 OR #30 OR #31 OR #32 OR #33
#35 randomized controlled trial: Topic
#36 controlled clinical trial: Topic
#37 #35 OR #36
#38 #34 OR #37 AND ('Article'/it OR 'Early Access'/it)

Table S1. Simplified clinical benefit scale for advanced disease[1,2]

Clinical benefit grade	If median OS in the control (standard) arm ≤ 12 months, the experimental arm median OS better by	If median OS in the control (standard) arm > 12 months and ≤ 24 months, the experimental arm median OS better by	If median OS in the control (standard) arm > 24 months, the experimental arm median OS better by
Grade 4	≥ 3 months	≥ 5 months	≥ 9 months
Grade 3	≥ 2 months and < 3 months	≥ 3 months and < 5 months	≥ 6 months and < 9 months
Grade 2	≥ 1.5 months and < 2 months	≥ 1.5 months and < 3 months	≥ 4 months and < 6 months
Grade 1	< 1.5 months	< 1.5 months	< 4 months

The modification is to restrict grading requirements to differences in the median OS.

The complete Form 2a has an additional grade 4 option based on an 10% increase in 2-year OS and requirements for all grades on the lower 95% confidence interval for the hazard ratio.

Additionally, the grade is moved up 1 level (possibly to a grade 5) if the quality of life is improved or there is a reduction in grade 3 and 4 toxicities impacting daily well-being.

Table S2. Assessment of study quality.

Studies	Study number	Adequate sequence generation	Allocation concealment	Blinding	Incomplete outcome data addressed	Free of selective reporting	Free of other bias
Paz-Ares et al. (2018) [3]	KEYNOTE-407	Yes	Yes	Yes	Yes	Yes	Yes
Socinski et al. (2018, 2021) [4,5]	IMpower150	Unclear	Unclear	No	Yes	Yes	Yes
Motzer et al. (2018) [6]	CheckMate 214	Unclear	Unclear	No	Yes	Yes	Yes
Rini et al. (2019) [7]	IMmotion151	Yes	Yes	No	Yes	Yes	Yes
West et al. (2019) [8]	IMpower130	Yes	Yes	No	Yes	Yes	Yes
Choueiri et al. (2020) [9]	JAVELIN Renal 101	Unclear	Yes	No	Yes	Yes	Yes
Galsky et al. (2020) [10]	IMvigor130	Yes	Yes	Partial	Yes	Yes	Yes
Gutzmer et al. (2020, 2023) [11,12]	IMspire150	Yes	Yes	Yes	Yes	Yes	Yes
Jotte et al. (2020) [13]	IMpower131	Unclear	Unclear	No	Yes	Yes	Yes
Powles et al. (2020) [14]	KEYNOTE-426	Yes	Yes	No	Yes	Yes	Yes
Rudin et al. (2020) [15]	KEYNOTE-604	Yes	Yes	Yes	Yes	Yes	Yes
Emens et al. (2021) [16,17]	IMpassion130	Yes	Yes	Yes	Yes	Yes	Yes
Janjigian et al. (2021, 2022) [18,19]	CheckMate 649	Yes	Yes	No	Yes	No	Yes
Liu et al. (2021) [20]	IMpower133	Yes	Yes	Yes	Yes	Yes	Yes
Luo et al. (2021) [21]	ESCORT-1st	Yes	Yes	Yes	Yes	Yes	Yes
Mai et al. (2021) [22]	NCT03581786	Yes	Yes	Yes	Yes	Yes	Yes
Miles et al. (2021) [23]	IMpassion131	Yes	Yes	Yes	Yes	Yes	Yes
Moehler et al. (2021)[24]	JAVELIN Gastric 100	Unclear	Unclear	No	Yes	No	Yes
Monk et al. (2021) [25]	JAVELIN Ovarian 100	Yes	Yes	No	Yes	Yes	Yes
Moore et al. (2021)[26]	IMagyn050/GOG 3015/ENGOT -OV39	Unclear	Yes	No	Yes	Yes	Yes
Nishio et al. (2021) [27]	IMpower132	Unclear	Unclear	No	Yes	Yes	Yes
Owonikoko et al. (2021) [28]	CheckMate 451	Yes	Yes	Yes	Yes	Yes	Yes
Reck et al. (2021)[29]	CheckMate 9LA	Yes	Unclear	No	Yes	Yes	Yes

Rodriguez-Abreu et al. (2021)[30]	KEYNOTE-189	Yes	Yes	Yes	Yes	Yes	Yes
Sugawara et al. (2021)[31]	TASUKI-52	Yes	Unclear	Yes	Yes	Yes	Yes
Zhou et al. (2021)[32]	CameL	Yes	Yes	No	Yes	Yes	Yes
Motzer et al. (2021) [33]	CLEAR	Yes	Yes	No	Yes	Yes	Yes
Powles et al. (2021) [34]	KEYNOTE-361	Yes	Yes	No	Yes	Yes	Yes
Sun et al. (2021) [35]	KEYNOTE-590	Yes	Yes	Yes	Yes	Yes	Yes
Burtness et al. (2022)[36]	KEYNOTE-048	Yes	Yes	No	Yes	Yes	Yes
Cortes et al. (2022) [37]	KEYNOTE-355	Yes	Yes	Yes	Yes	Yes	Yes
Doki et al. (2022)[38]	CheckMate 648	Yes	Yes	Yes	Yes	Yes	Yes
Paz-Ares et al. (2022) [39]	CheckMate 227	Yes	Yes	No	Yes	Yes	Yes
Peters et al. (2022) [40]	CheckMate 743	Yes	Yes	No	Yes	Yes	Yes
Spigel et al. (2022) [41]	PACIFIC	Yes	Yes	Yes	Yes	Yes	Yes
Wolchok et al. (2022)[42]	CheckMate 067	Yes	Yes	Yes	Yes	Yes	Yes
Yau et al. (2022)[43]	CheckMate 459	Yes	Yes	No	Yes	Yes	Yes
Zhou et al. (2022)[44]	GEMSTONE-302	Yes	Yes	Yes	Yes	Yes	Yes
Cheng et al. (2022) [45]	IMbrave150	Yes	Yes	No	Yes	Yes	Yes
Gogishvili et al. (2022) [46]	EMPOWER-Lung 3	Yes	Yes	Yes	Yes	Yes	Yes
Kang et al. (2022) [47]	ATTRACTION-4	Yes	Yes	Yes	Yes	Yes	Yes
Lu et al. (2022) [48]	ORIENT-15	Yes	Yes	Yes	Yes	Yes	Yes
Motzer et al. (2022) [49]	CheckMate 9ER	Yes	Yes	No	Yes	Yes	Yes
Wang et al. (2022) [50]	CAPSTONE-1	Yes	Yes	Yes	Yes	Yes	Yes
Cheng et al. (2022) [51]	ASTRUM-005	Yes	Yes	Yes	Yes	Yes	Yes
Johnson et al. (2022) [52]	POSEIDON	Unclear	Unclear	No	Yes	Yes	Yes
Dummer et al. (2022) [53]	COMBI-i	Yes	Yes	Yes	Yes	Yes	Yes
Wu et al. (2022) [54,55]	JUPITER-06	Yes	Yes	Yes	Yes	Yes	Yes
de Castro et a. (2023) [56]	NEPTUNE	Unclear	Unclear	No	Yes	No	Yes

Table S3. Summary of where and how the IPD of low PD-L1 population were extracted

**Studies**	**Study number**	**Outcome**	**The position of "Overall" curve①**	**The position of "High PD-L1" curve ②**	**The position of "Low PD-L1" curve③**	**How the IPD of low PDL1 extracted**
Paz-Ares et al. (2018) [3]	KEYNOTE-407	OS	Fig 1A	Fig S3B-C	Fig S3A	IPDfromKM: ③
		PFS	Fig 2A	Fig 53B-C	Fig S5A	IPDfromKM: ③
Socinski et al. (2018) [4,5]	IMpower150	OS[5]	Fig 1B	Fig 2B	Fig 2C	IPDfromKM: ③
		PFS[4]	Fig 1A	Fig S5B, Fig S6B	Fig S6A	IPDfromKM: ③
Motzer et al. (2018) [6]	CheckMate 214	OS	Fig 1A	Fig S4	Fig S4	IPDfromKM: ③
Rini et al. (2019) [7]	IMmotion151	OS	Fig 2D	Fig 2C	NR	IPDfromKM & KMSubtraction: ①-②
		PFS	Fig 2B	Fig 2A	NR	IPDfromKM & KMSubtraction: ①-②
Choueiri, et al. (2020) [9]	JAVELIN Renal 101	OS	Fig 1D	Fig 1C	NR	IPDfromKM & KMSubtraction: ①-②
		PFS	Fig 1B	Fig 1A	NR	IPDfromKM & KMSubtraction: ①-②

Emens et al. (2021) [16,17]	IMpassion130	OS [16]	Fig 1A	Fig S2	Fig S2	IPDfromKM: ③
		PFS [17]	Fig 4A	Fig 4B	Fig 4B	IPDfromKM: ③
Janjigian et al. (2021) [19]	CheckMate 649	OS	Fig 2C	Fig 2B	NR	IPDfromKM & KMSubtraction: ①-②
		PFS	Fig 3C	Fig 3B	NR	IPDfromKM & KMSubtraction: ①-②
Liu et al. (2021) [20]	IMpower133	OS	Fig 2A	Fig 3A	Fig 3A	IPDfromKM: ③
		PFS	NR	Fig 3B	Fig 3B	IPDfromKM: ③
Luo et al. (2021) [21]	ESCORT-1st	OS	Fig 1A	Fig S5B	Fig S5B	IPDfromKM: ③
		PFS	Fig 1B	Fig S7B	Fig S7A	IPDfromKM: ③
Mai et al. (2021) [22]	NCT03581786	PFS	Fig 1A	Fig S2	Fig S2	IPDfromKM: ③
Miles et al. (2021) [23]	IMpassion131	OS	Fig 3B	Fig 3A	NR	IPDfromKM & KMSubtraction: ①-②
		PFS	Fig 2B	Fig 2A	NR	IPDfromKM & KMSubtraction: ①-②
Moehler et al. (2021)[24]	JAVELIN Gastric 100	OS	Fig 2A	Fig 2C	NR	IPDfromKM & KMSubtraction: ①-②
		PFS	Fig S2A	Fig S2C	NR	IPDfromKM & KMSubtraction: ①-②
Monk et al. (2021) [25]	JAVELIN Ovarian 100	PFS	Fig 2	Fig S2A	Fig S2B	IPDfromKM: ③
Moore et al. (2021)[26]	IMagyn050/GOG 3015/ENGOT -OV39	OS	Fig 2C	Fig 2D	NR	IPDfromKM & KMSubtraction: ①-②
		PFS	Fig 2A	Fig 2B	NR	IPDfromKM & KMSubtraction: ①-②
Nishio et al. (2021) [27]	IMpower132	OS	Fig 3A	Fig 3B	Fig 3B	IPDfromKM: ③
		PFS	Fig 2A	Fig 2B	Fig 2B	IPDfromKM: ③
Owonikoko et al. (2021) [28]	CheckMate 451	OS	Fig 2A-B	Fig 3C	Fig 3D	IPDfromKM: ③
Reck et al. (2021)[29]	CheckMate 9LA	OS	Fig 1A	Fig 1C	Fig 1B	IPDfromKM: ③
		PFS	Fig S4A	Fig S5D	Fig S5C	IPDfromKM: ③
Rodriguez-Abreu et al. (2021)[30]	KEYNOTE-189	OS	Fig 1A	Fig 1B-C	Fig 1D	IPDfromKM: ③
		PFS	Fig 2A	Fig 2B-C	Fig 2D	IPDfromKM: ③
Sugawara et al. (2021)[31]	TASUKI-52	PFS	Fig 2A	Fig 3B-C	Fig 3A	IPDfromKM: ③
Zhou et al. (2021)[32]	CameL	PFS	Fig 2A	Fig S1	NR	IPDfromKM & KMSubtraction: ①-②
Powles et al. (2021) [34]	KEYNOTE-361	OS	Fig 3B	Fig 3A	NR	IPDfromKM & KMSubtraction: ①-②
Sun et al. (2021) [35]	KEYNOTE-590	OS	Fig 2D	Fig 2C	Fig 2E	IPDfromKM: ③
		PFS	Fig 4C	Fig 4B	Fig 4D	IPDfromKM: ③
Burtness et al. (2022)[36]	KEYNOTE-048	OS	NR	NR	Fig 1A, C	IPDfromKM: ③
		PFS	NR	NR	Fig 3A, D	IPDfromKM: ③
Cortes et al. (2022) [37]	KEYNOTE-355	OS	Fig 1C	Fig 1B	NR	IPDfromKM & KMSubtraction: ①-②
		PFS	Fig S2C	Fig S2B	NR	IPDfromKM & KMSubtraction: ①-②
Doki et al. (2022)[38]	CheckMate 648	OS	Fig 1B, Fig 2B	Fig 1A, Fig 2A	NR	IPDfromKM & KMSubtraction: ①-②
		PFS	Fig 1D, Fig 2D	Fig 1C, Fig 2C	NR	IPDfromKM &

						KMSubtraction: ①-②
Paz-Ares et al. (2022) [39]	CheckMate 227	OS	NRNR	Fig 1A	Fig 1C	IPDfromKM: ③
		PFS		Fig 3A	Fig 4A	IPDfromKM: ③
Peters et al. (2022) [40]	CheckMate 743	OS	Fig 1A	Fig S3A	Fig S3B	IPDfromKM: ③
Spigel et al. (2022) [41]	PACIFIC	OS	Fig 2A	Fig S2C	Fig S2D	IPDfromKM: ③
		PFS	Fig 2B	Fig S3C	Fig S3D	IPDfromKM: ③
Wolchok et al. (2022)[42]	CheckMate 067	OS	Fig 2B	Fig S1B	Fig S1A	IPDfromKM: ③
Yau et al. (2022)[43]	CheckMate 459	OS	Fig 2A	Fig S1A	Fig S1B	IPDfromKM: ③
		PFS	Fig 3	Fig S3A	Fig S3B	IPDfromKM: ③
Zhou et al. (2022)[44]	GEMSTONE-302	PFS	Fig 2A	Fig S4A-B	Fig S4C	IPDfromKM: ③
Dummer et al. (2022) [53]	COMBI-i	PFS	Fig 2A	Fig 3A	Fig 3A	IPDfromKM: ③
Wu et al. (2022) [54,55]	JUPITER-06	OS	NR	Fig 1C	Fig 1D	IPDfromKM: ③
		PFS	NR	Fig 1A	Fig 1B	IPDfromKM: ③
Lu et al. (2022) [48]	ORIENT-15	OS	Fig 2A	Fig 2B	NR	IPDfromKM & KMSubtraction: ①-②
		PFS	Fig 4A	Fig 4B	NR	IPDfromKM & KMSubtraction: ①-②
de Castro et a. (2023) [56]	NEPTUNE	OS	Fig S4A	NR	Fig S3C	IPDfromKM: ③

OS, overall survival; PFS, progression-free survival; PDL1, programmed death-ligand 1

Table S4. Comparisons to original curves for overall, PDL1 ≥ 1%, and PDL1 < 1% populations.

Study, outcome, cohort	Original	Reconstructed
KEYNOTE-407, overall survival, TPS < 1%	The original figure may be found in the primary trial manuscript DOI: 10.1056/NEJMoa1810865 Fig S3A	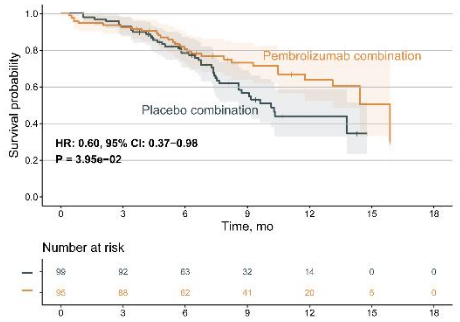
KEYNOTE-407, progression-free survival, TPS < 1%	The original figure may be found in the primary trial manuscript DOI: 10.1056/NEJMoa1810865 Fig S5A	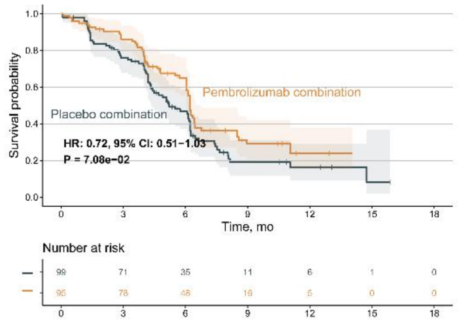
IMpower150, overall survival, TPS&IPS < 1%	The original figure may be found in the primary trial manuscript DOI: 10.1016/j.jtho.2021.07.009 Fig 2C	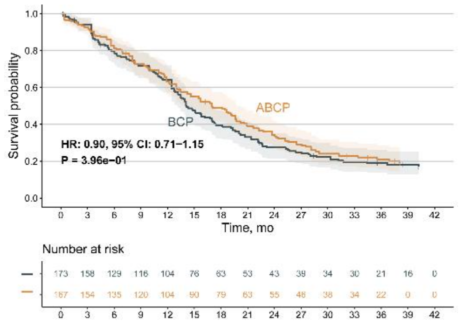

IMpower150, progression-free survival, TPS&IPS < 1%	The original figure may be found in the primary trial manuscript<br>DOI: 10.1056/NEJMoa1716948<br>Fig S6B	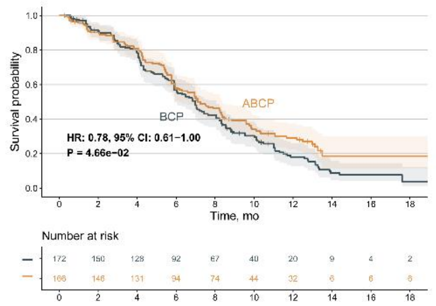
CheckMate 214, overall survival, TPS < 1%	The original figure may be found in the primary trial manuscript<br>DOI: 10.1056/NEJMoa1712126<br>Fig S4	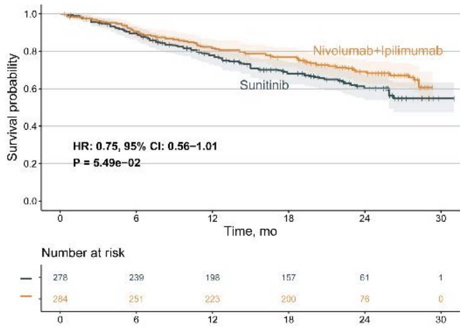
IMmotion151, overall survival, Overall	The original figure may be found in the primary trial manuscript<br>DOI:<br>10.1016/S0140-6736(19)30723-8<br>Fig 2D	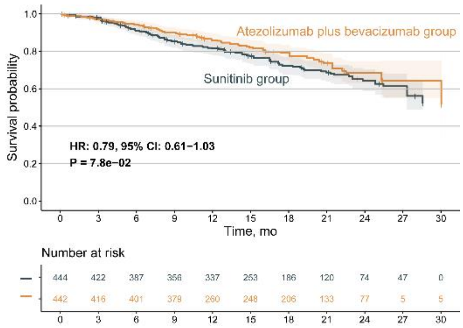
IMmotion151, overall survival, IPS ≥ 1%	The original figure may be found in the primary trial manuscript<br>DOI:<br>10.1016/S0140-6736(19)30723-8<br>Fig 2C	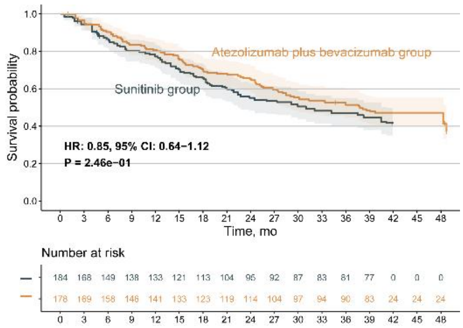
IMmotion151, progression-free survival, Overall	The original figure may be found in the primary trial manuscript<br>DOI:<br>10.1016/S0140-6736(19)30723-8<br>Fig 2B	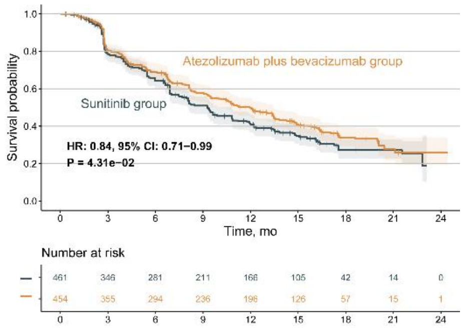
IMmotion151, progression-free survival, IPS ≥ 1%	The original figure may be found in the primary trial manuscript<br>DOI:<br>10.1016/S0140-6736(19)30723-8<br>Fig 2A	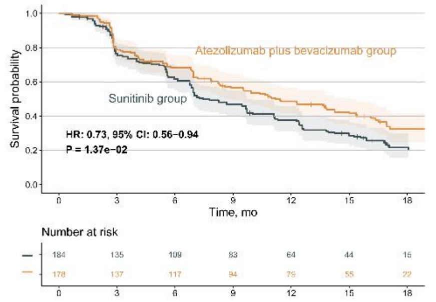

JAVELIN Renal 101, overall survival, Overall	The original figure may be found in the primary trial manuscript<br>DOI: 10.1016/j.annonc.2020.04.010<br>Fig 1D	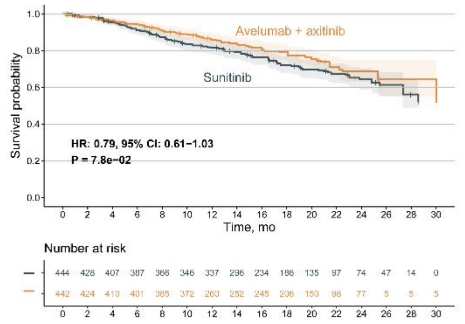
JAVELIN Renal 101, overall survival, IPS ≥ 1%	The original figure may be found in the primary trial manuscript<br>DOI: 10.1016/j.annonc.2020.04.010<br>Fig 1C	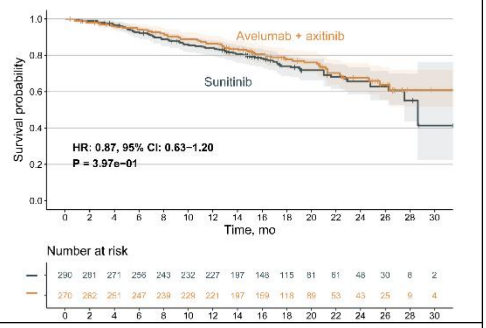
JAVELIN Renal 101, progression-free survival, Overall	The original figure may be found in the primary trial manuscript<br>DOI: 10.1016/j.annonc.2020.04.010<br>Fig 1B	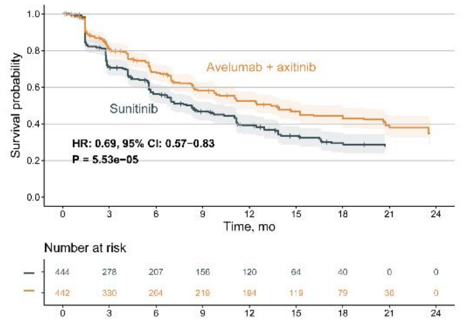
JAVELIN Renal 101, progression-free survival, IPS ≥ 1%	The original figure may be found in the primary trial manuscript<br>DOI: 10.1016/j.annonc.2020.04.010<br>Fig 1A	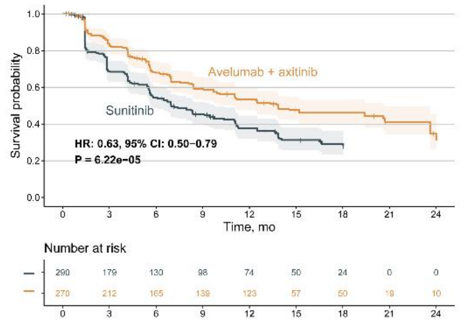
IMpassion130, overall survival, IPS < 1%	The original figure may be found in the primary trial manuscript<br>DOI: 10.1016/j.annonc.2021.05.355<br>Fig S2	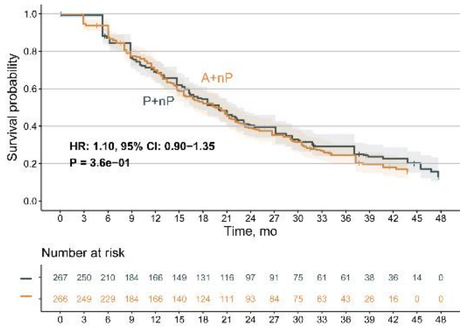

IMpassion130, progression-free survival, IPS < 1%	The original figure may be found in the primary trial manuscript<br>DOI:<br>10.1016/s1470-2045(19)30689-8<br>Fig 4B	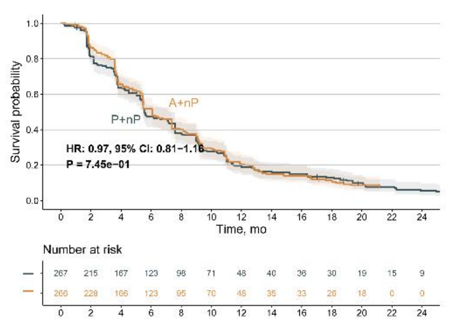
CheckMate 649, overall survival, Overall	The original figure may be found in the primary trial manuscript<br>DOI:<br>10.1016/S0140-6736(21)00797-2<br>Fig 2C	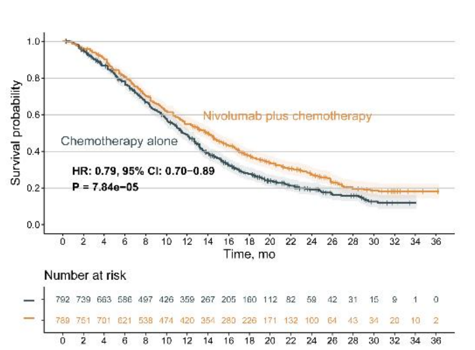
CheckMate 649, overall survival, CPS > 1	The original figure may be found in the primary trial manuscript<br>DOI:<br>10.1016/S0140-6736(21)00797-2<br>Fig 2B	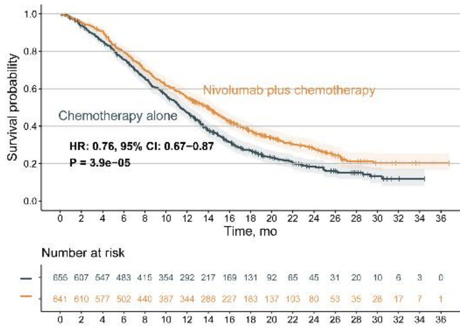
CheckMate 649, progression-free survival, Overall	The original figure may be found in the primary trial manuscript<br>DOI:<br>10.1016/S0140-6736(21)00797-2<br>Fig 3C	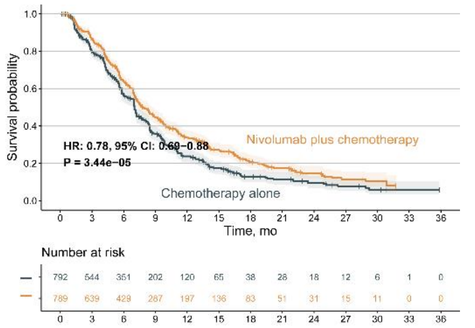
CheckMate 649, progression-free survival, CPS > 1	The original figure may be found in the primary trial manuscript<br>DOI:<br>10.1016/S0140-6736(21)00797-2<br>Fig 3B	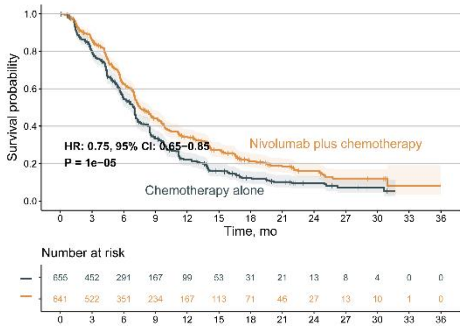
IMpower133, overall survival, TPS&IPS < 1%	The original figure may be found in the primary trial manuscript<br>DOI: 10.1200/JCO.20.01055<br>Fig 3A	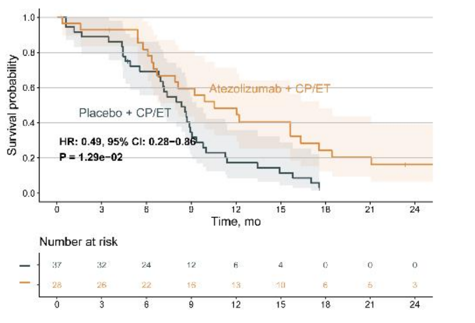

IMpower133, progression-free survival, TPS&IPS < 1%	The original figure may be found in the primary trial manuscript<br>DOI: 10.1200/JCO.20.01055<br>Fig 3B	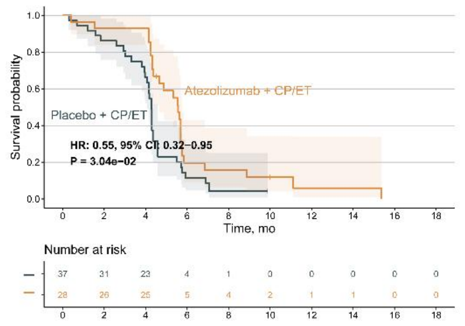
ESCORT-1st, overall survival, TPS < 1%	The original figure may be found in the primary trial manuscript<br>DOI: 10.1001/jama.2021.12836<br>Fig S5B	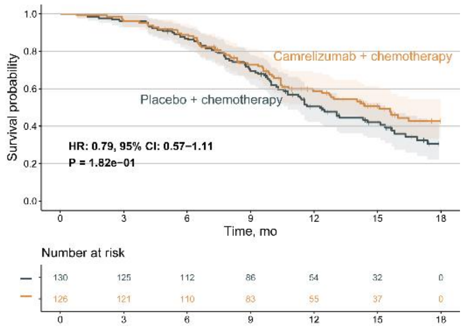
ESCORT-1st, progression-free survival, TPS < 1%	The original figure may be found in the primary trial manuscript<br>DOI: 10.1001/jama.2021.12836<br>Fig S7A	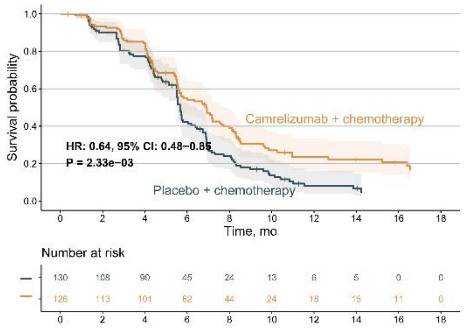
NCT03581786, progression-free survival, TPS&IPS < 1%	The original figure may be found in the primary trial manuscript<br>DOI: 10.1038/s41591-021-01444-0<br>Fig S2	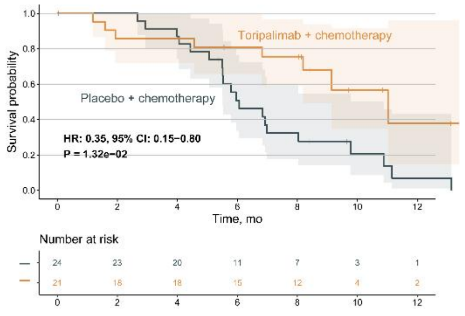
IMpassion131, overall survival, Overall	The original figure may be found in the primary trial manuscript<br>DOI: 10.1016/j.annonc.2021.05.801<br>Fig 3B	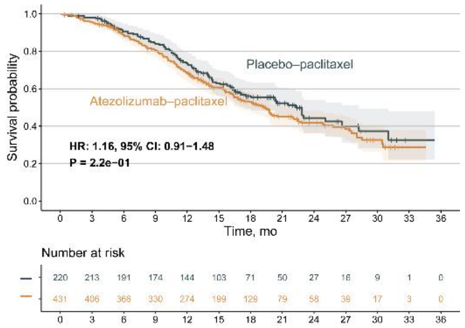

IMpassion131, overall survival, IPS ≥ 1%	The original figure may be found in the primary trial manuscript<br>DOI: 10.1016/j.annonc.2021.05.801<br>Fig 3A	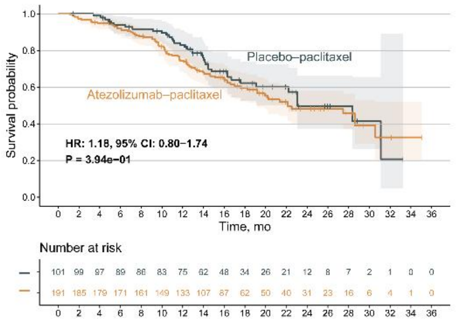
IMpassion131, progression-free survival, Overall	The original figure may be found in the primary trial manuscript<br>DOI: 10.1016/j.annonc.2021.05.801<br>Fig 2B	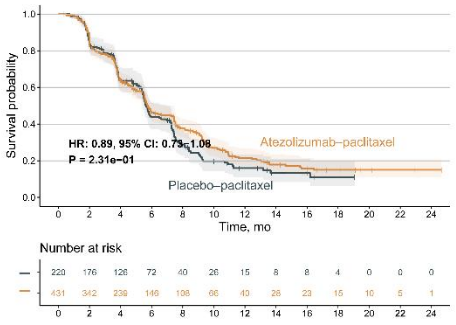
IMpassion131, progression-free survival, IPS ≥ 1%	The original figure may be found in the primary trial manuscript<br>DOI: 10.1016/j.annonc.2021.05.801<br>Fig 2A	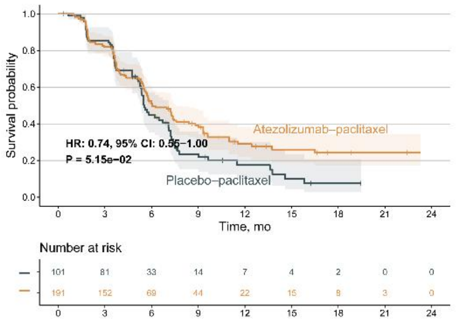
JAVELIN Gastric 100, overall survival, Overall	The original figure may be found in the primary trial manuscript<br>DOI: 10.1200/JCO.20.00892<br>Fig 2A	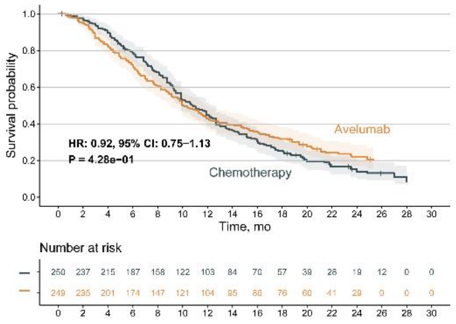
JAVELIN Gastric 100, overall survival, CPS ≥1	The original figure may be found in the primary trial manuscript<br>DOI: 10.1200/JCO.20.00892<br>Fig 2C	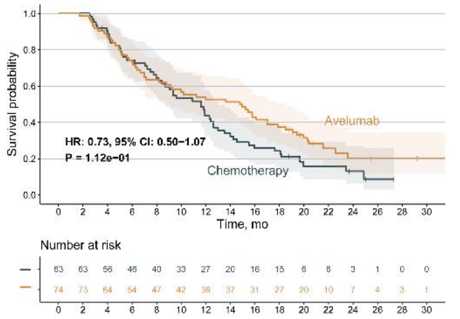

JAVELIN Gastric 100, progression-free survival, Overall	The original figure may be found in the primary trial manuscript<br>DOI: 10.1200/JCO.20.00892<br>Fig S2A	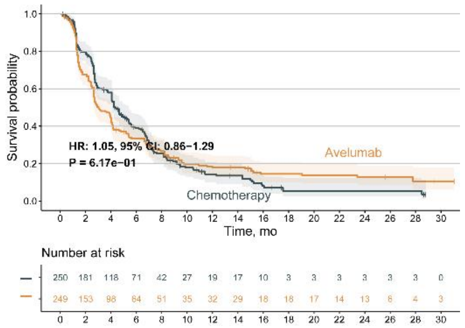
JAVELIN Gastric 100, progression-free survival, CPS ≥1	The original figure may be found in the primary trial manuscript<br>DOI: 10.1200/JCO.20.00892<br>Fig S2C	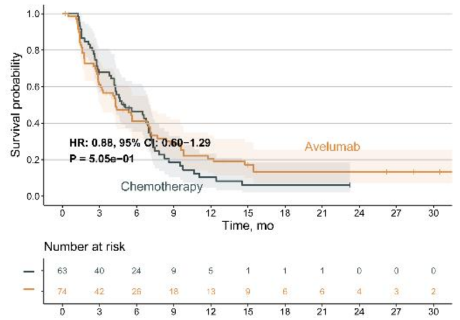
JAVELIN Ovarian 100, progression-free survival, TPS&IPS < 1%	The original figure may be found in the primary trial manuscript<br>DOI:<br>10.1016/S1470-2045(21)00342-9<br>Fig S2B	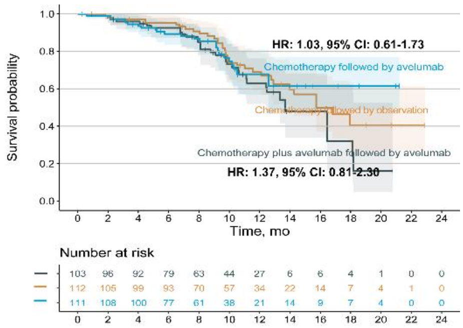
IMagyn050/GOG 3015/ENGOT-OV39, overall survival, Overall	The original figure may be found in the primary trial manuscript<br>DOI: 10.1200/JCO.21.00306<br>Fig 2C	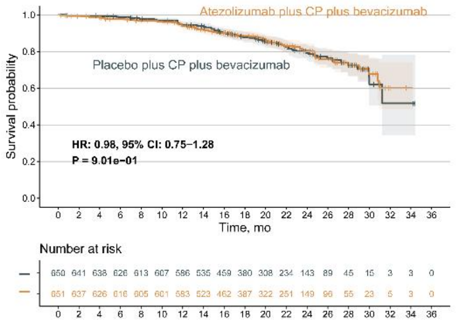
IMagyn050/GOG 3015/ENGOT-OV39, overall survival, IPS ≥ 1%	The original figure may be found in the primary trial manuscript<br>DOI: 10.1200/JCO.21.00306<br>Fig 2D	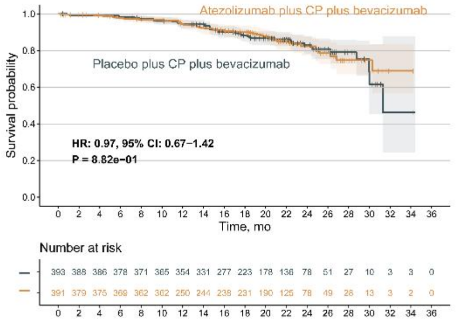

IMagyn050/GOG 3015/ENGOT-OV39, progression-free survival, Overall	The original figure may be found in the primary trial manuscript<br>DOI: 10.1200/JCO.21.00306<br>Fig 2B	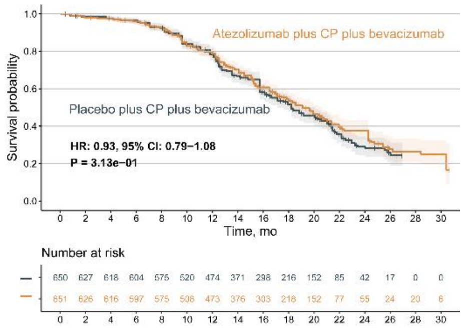
IMagyn050/GOG 3015/ENGOT-OV39, progression-free survival, IPS ≥ 1%	The original figure may be found in the primary trial manuscript<br>DOI: 10.1200/JCO.21.00306<br>Fig 2A	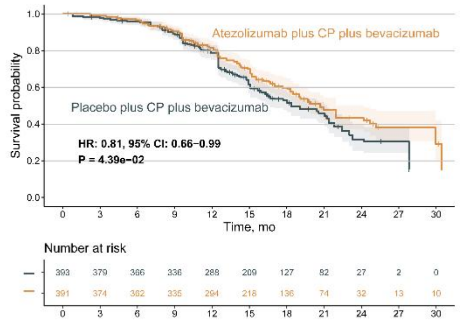
IMpower132, overall survival, TPS&IPS < 1%	The original figure may be found in the primary trial manuscript<br>DOI: 10.1016/j.jtho.2020.11.025<br>Fig 3B	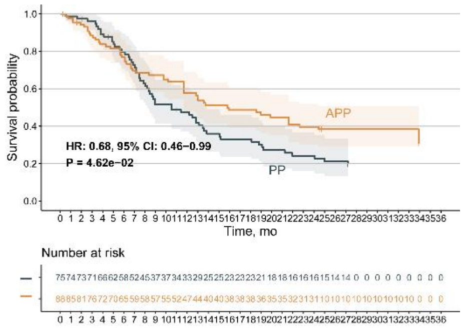
IMpower132, progression-free survival, TPS&IPS < 1%	The original figure may be found in the primary trial manuscript<br>DOI: 10.1016/j.jtho.2020.11.025<br>Fig 2B	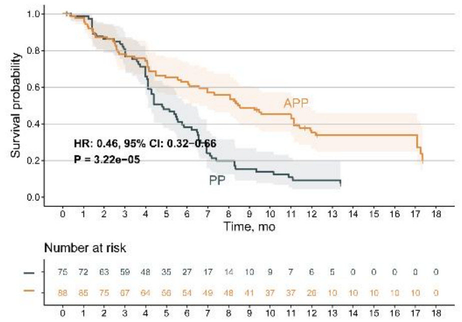
CheckMate 451, overall survival, CPS < 1	The original figure may be found in the primary trial manuscript<br>DOI: 10.1200/JCO.20.02212<br>Fig 3D	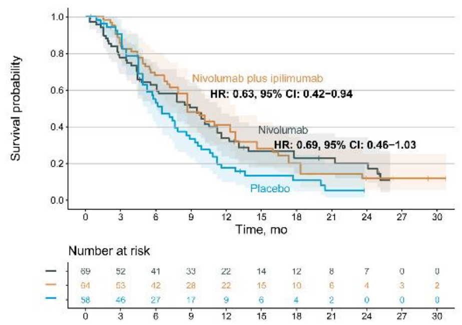

CheckMate 9LA, overall survival, TPS < 1%	The original figure may be found in the primary trial manuscript<br>DOI: 10.1016/j.esmoop.2021.100273<br>Fig 1B	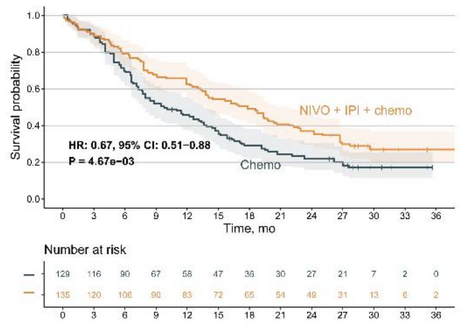
CheckMate 9LA, progression-free survival, TPS < 1%	The original figure may be found in the primary trial manuscript<br>DOI: 10.1016/j.esmoop.2021.100273<br>Fig S5C	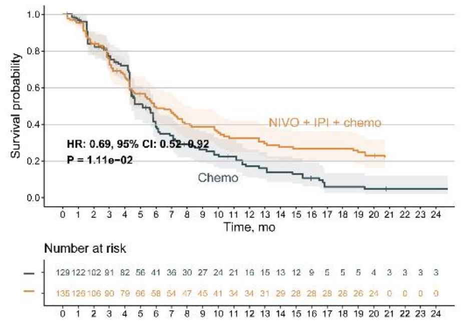
KEYNOTE-189, overall survival, TPS < 1%	The original figure may be found in the primary trial manuscript<br>DOI: 10.1016/j.annonc.2021.04.008<br>Fig 1D	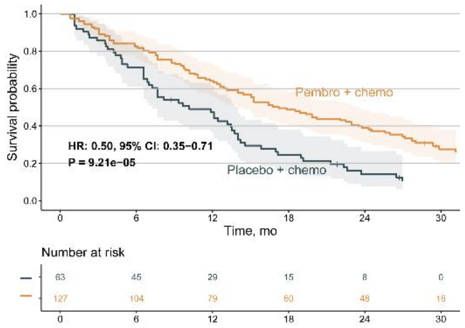
KEYNOTE-189, progression-free survival, TPS < 1%	The original figure may be found in the primary trial manuscript<br>DOI: 10.1016/j.annonc.2021.04.008<br>Fig 2D	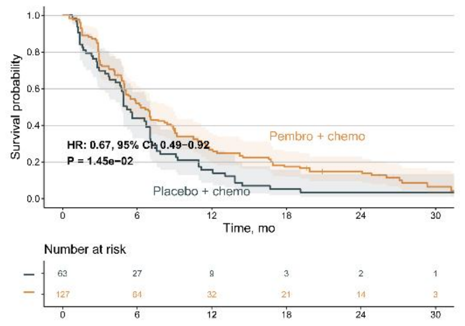
TASUKI-52, progression-free survival, TPS < 1%	The original figure may be found in the primary trial manuscript<br>DOI: 10.1016/j.annonc.2021.06.004<br>Fig 3A	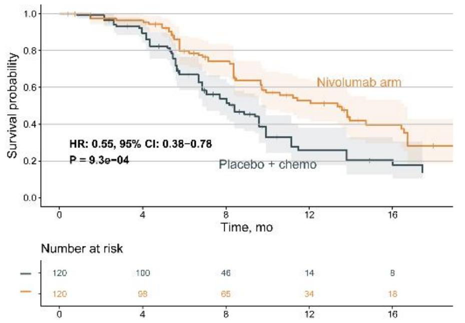

CameL, progression-free survival, Overall	The original figure may be found in the primary trial manuscript DOI: 10.1016/S2213-2600(20)30365-9 Fig 2A	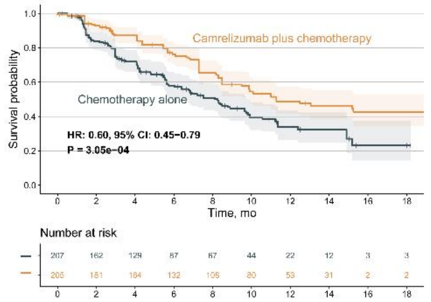
CameL, progression-free survival, TPS ≥ 1%	The original figure may be found in the primary trial manuscript DOI: 10.1016/S2213-2600(20)30365-9 Fig S1	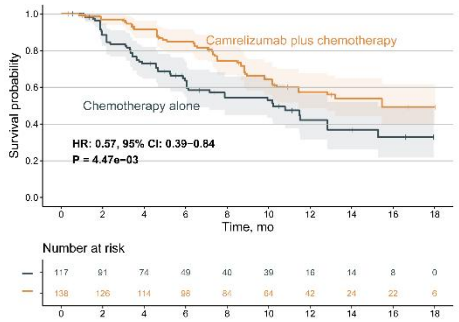
KEYNOTE-361, overall survival, Overall	The original figure may be found in the primary trial manuscript DOI: 10.1016/s1470-2045(21)00152-2 Fig 3B	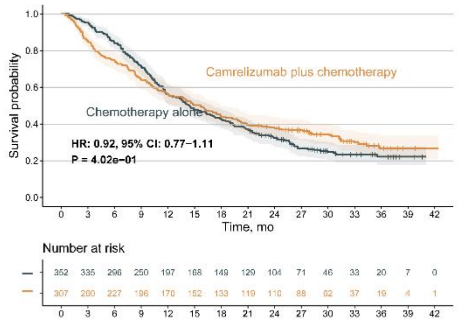
KEYNOTE-361, overall survival, TPS ≥ 1%	The original figure may be found in the primary trial manuscript DOI: 10.1016/s1470-2045(21)00152-2 Fig 3A	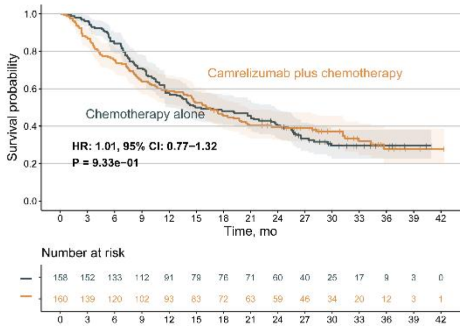
KEYNOTE-048 (Pembrolizumab), overall survival, CPS < 1	The original figure may be found in the primary trial manuscript DOI: 10.1200/JCO.21.02198 Fig 2A	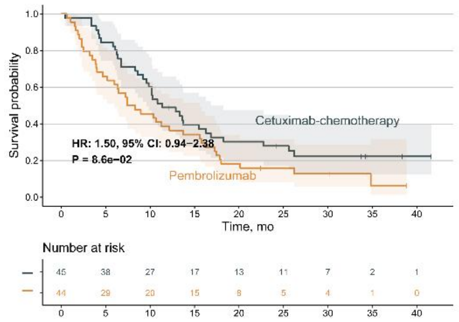

KEYNOTE-048 (Pembrolizumab), progression-free survival, CPS < 1	The original figure may be found in the primary trial manuscript DOI: 10.1200/JCO.21.02198 Fig 3A	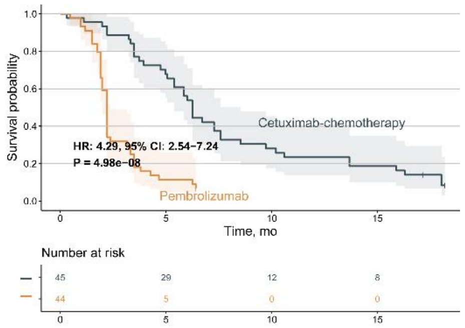
KEYNOTE-048 (Pembrolizumab-chemotherapy), overall survival, CPS < 1	The original figure may be found in the primary trial manuscript DOI: 10.1200/JCO.21.02198 Fig 2C	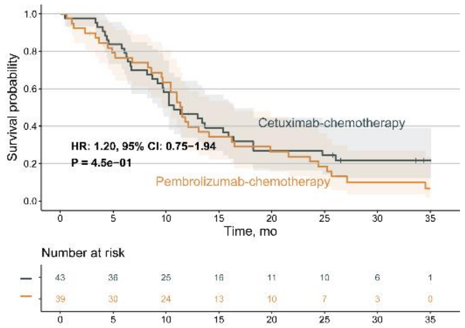
KEYNOTE-048 (Pembrolizumab-chemotherapy), progression-free survival, CPS < 1	The original figure may be found in the primary trial manuscript DOI: 10.1200/JCO.21.02198 Fig 3D	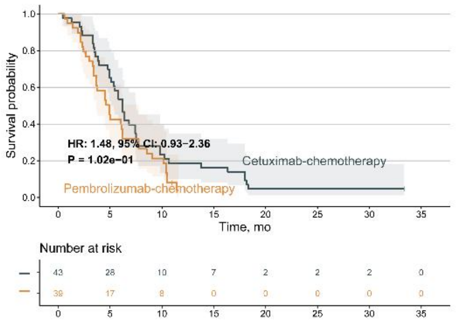
KEYNOTE-590, overall survival, TPS < 1	The original figure may be found in the primary trial manuscript DOI: 10.1200/JCO.21.02198 Fig 2C	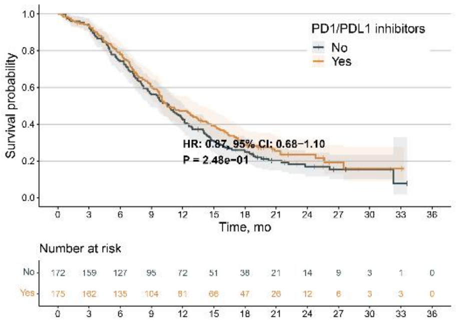
KEYNOTE-590, progression-free survival, TPS < 1	The original figure may be found in the primary trial manuscript DOI:10.1016/S0140-6736(21)01234-4 Fig 2E	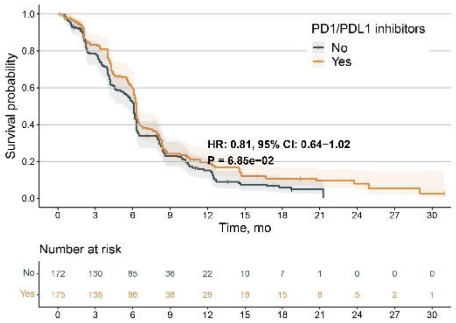

<table>
<tr><td>KEYNOTE-355, overall survival, Overall</td><td>The original figure may be found in the primary trial manuscript
DOI: 0.1016/S0140-6736(21)01234-4
Fig 4D</td><td>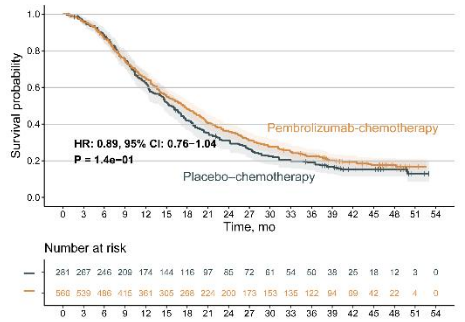
</td></tr>
<tr><td>KEYNOTE-355, overall survival, CPS ≥ 1</td><td>The original figure may be found in the primary trial manuscript
DOI: 10.1056/NEJMoa2202809
Fig 1B</td><td>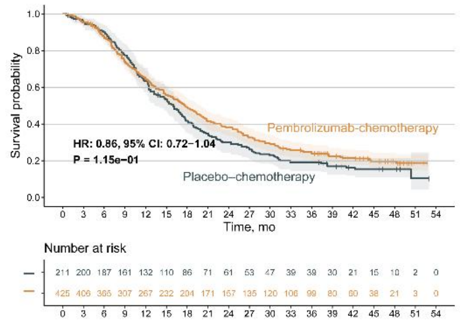
</td></tr>
<tr><td>KEYNOTE-355, progression-free survival, Overall</td><td>The original figure may be found in the primary trial manuscript
DOI: 10.1056/NEJMoa2202809
Fig S2C</td><td>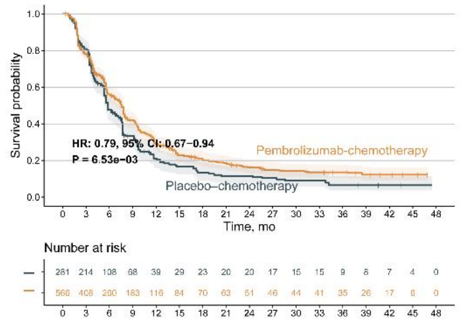
</td></tr>
<tr><td>KEYNOTE-355, progression-free survival, CPS ≥ 1</td><td>The original figure may be found in the primary trial manuscript
DOI: 10.1056/NEJMoa2202809
Fig S2B</td><td>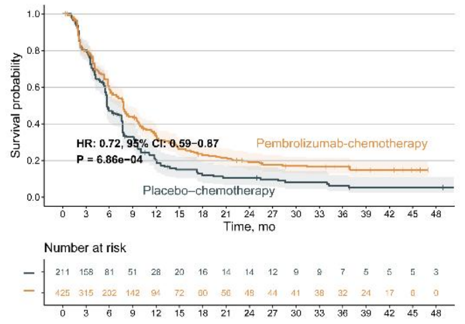
</td></tr>
<tr><td>CheckMate 648, overall survival (Nivolumab+chemotherapy), Overall</td><td>The original figure may be found in the primary trial manuscript
DOI: 10.1056/NEJMoa2111380
Fig 1B</td><td>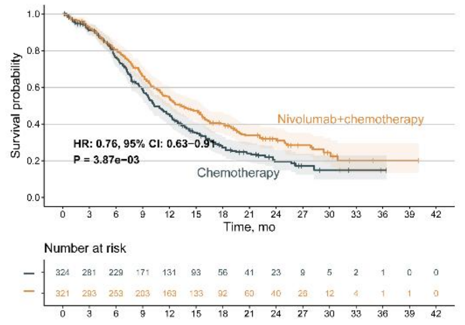
</td></tr>
</table>

CheckMate 648 (Nivolumab+chemotherapy), overall survival, TPS ≥ 1%	The original figure may be found in the primary trial manuscript DOI: 10.1056/NEJMoa2111380 Fig 1A	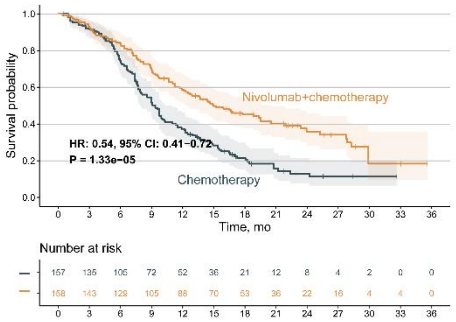
CheckMate 648 (Nivolumab+chemotherapy), progression-free survival, Overall	The original figure may be found in the primary trial manuscript DOI: 10.1056/NEJMoa2111380 Fig 1D	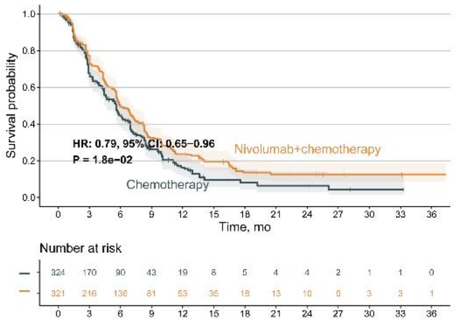
CheckMate 648 (Nivolumab+chemotherapy), progression-free survival, TPS ≥ 1%	The original figure may be found in the primary trial manuscript DOI: 10.1056/NEJMoa2111380 Fig 1C	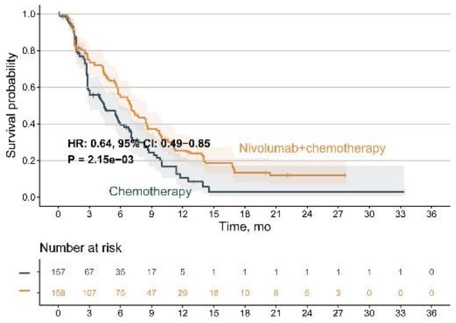
CheckMate 648, overall survival (Nivolumab+ipilimumab), Overall	The original figure may be found in the primary trial manuscript DOI: 10.1056/NEJMoa2111380 Fig 2B	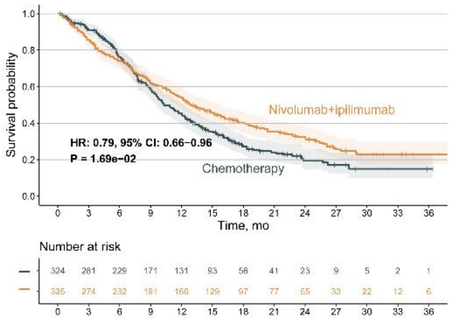
CheckMate 648 (Nivolumab+ipilimumab), overall survival, TPS ≥ 1%	The original figure may be found in the primary trial manuscript DOI: 10.1056/NEJMoa2111380 Fig 2A	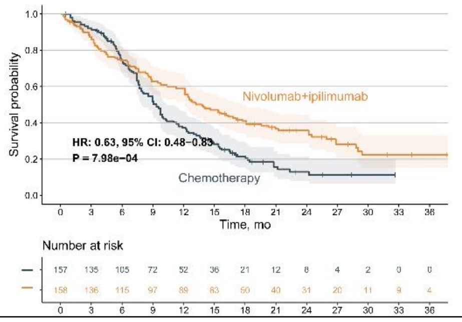

CheckMate 648 (Nivolumab+ipilimumab), progression-free survival, Overall	The original figure may be found in the primary trial manuscript DOI: 10.1056/NEJMoa2111380 Fig 2D	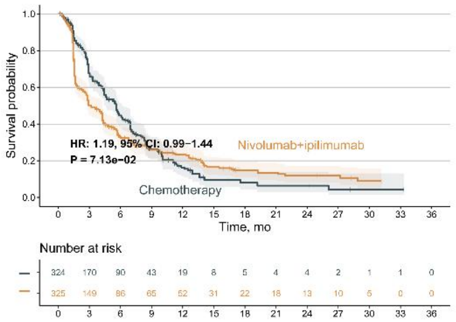
CheckMate 648 (Nivolumab+ipilimumab), progression-free survival, TPS ≥ 1%	The original figure may be found in the primary trial manuscript DOI: 10.1056/NEJMoa2111380 Fig 2C	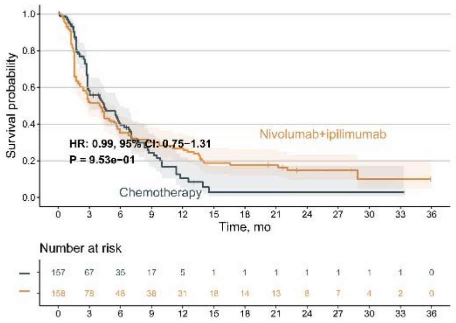
CheckMate 227, overall survival, TPS < 1%	The original figure may be found in the primary trial manuscript DOI: 10.1016/j.jtho.2021.09.010 Fig 1C	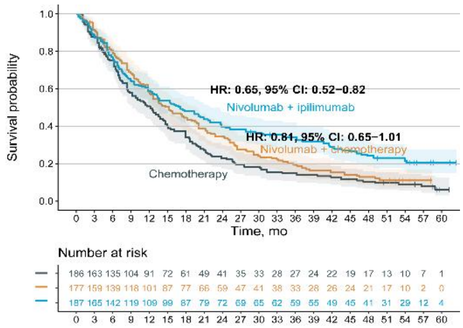
CheckMate 227, progression-free survival, TPS < 1%	The original figure may be found in the primary trial manuscript DOI: 10.1016/j.jtho.2021.09.010 Fig 4A	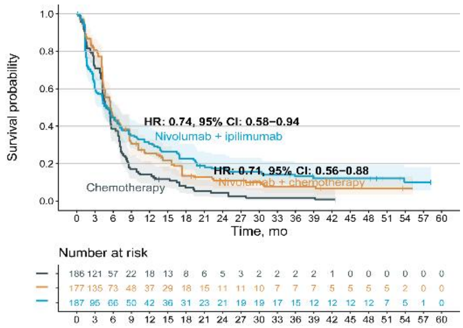
CheckMate 743, overall survival, TPS < 1%	The original figure may be found in the primary trial manuscript DOI: j.annonc.2022.01.074 Fig S3B	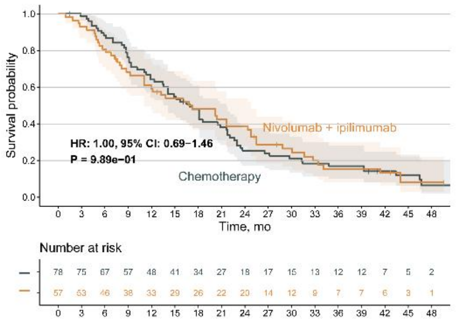

PACIFIC, overall survival, TPS < 1%	The original figure may be found in the primary trial manuscript<br>DOI: 10.1200/JCO.21.01308<br>Fig S2D	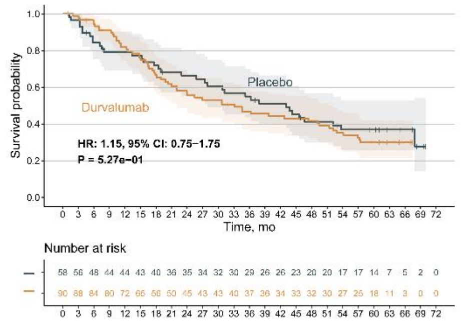
PACIFIC, progression-free survival, TPS < 1%	The original figure may be found in the primary trial manuscript<br>DOI: 10.1200/JCO.21.01308<br>Fig S3D	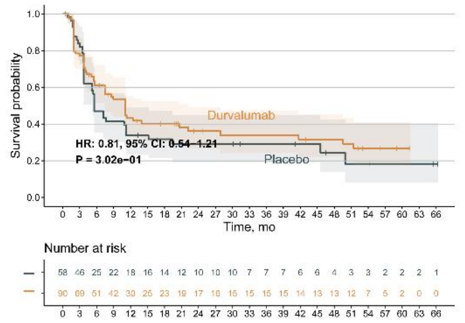
CheckMate 067, overall survival, TPS < 1%	The original figure may be found in the primary trial manuscript<br>DOI: 10.1200/JCO.21.02229<br>Fig S1A	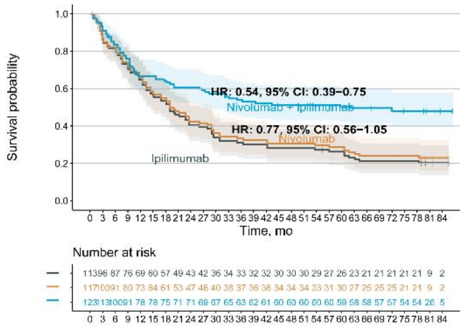
CheckMate 459, overall survival, TPS < 1%	The original figure may be found in the primary trial manuscript<br>DOI:<br>10.1016/S1470-2045(21)00604-5<br>Fig S1B	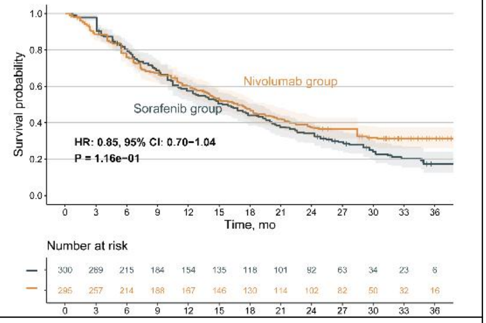
CheckMate 459, progression-free survival, TPS < 1%	The original figure may be found in the primary trial manuscript<br>DOI:<br>10.1016/S1470-2045(21)00604-5<br>Fig S3B	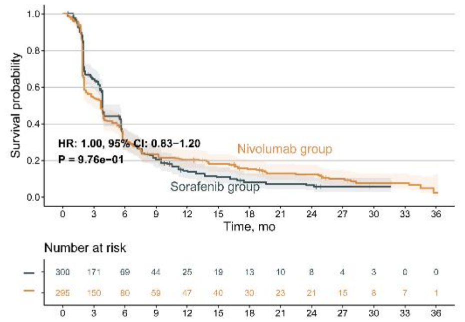

GEMSTONE-302, progression-free survival, TPS < 1%	The original figure may be found in the primary trial manuscript<br>DOI: 10.1016/S1470-2045(21)00650-1<br>Fig S4C	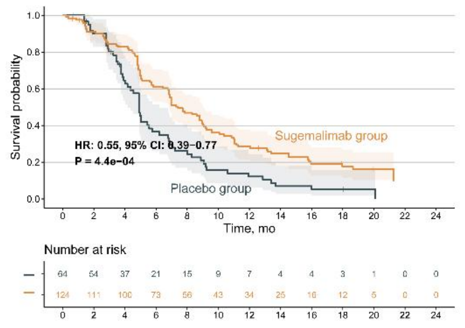
COMBI-i, progression-free survival, TPS < 1%	The original figure may be found in the primary trial manuscript<br>DOI: 10.1200/JCO.21.01601<br>Fig 3A	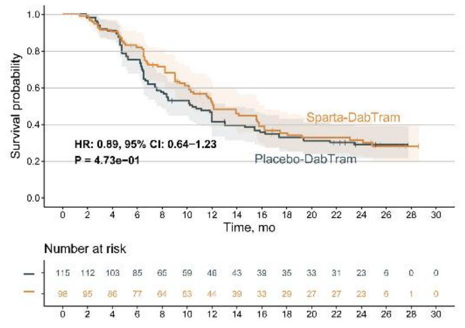
JUPITER-06, overall survival, TPS < 1%	The original figure may be found in the primary trial manuscript<br>DOI: 10.1200/JCO.22.01490<br>Fig 1D	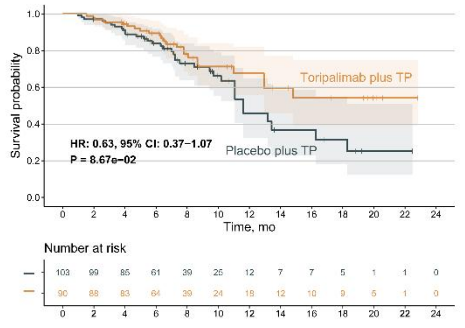
JUPITER-06, progression-free survival, TPS < 1%	The original figure may be found in the primary trial manuscript<br>DOI: 10.1200/JCO.22.01490<br>Fig 1B	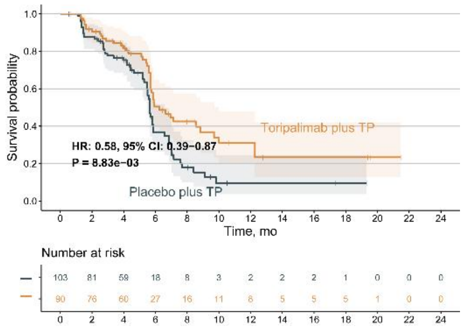
ORIENT-15, overall survival, Overall	The original figure may be found in the primary trial manuscript<br>DOI: 10.1136/bmj-2021-068714<br>Fig 2A	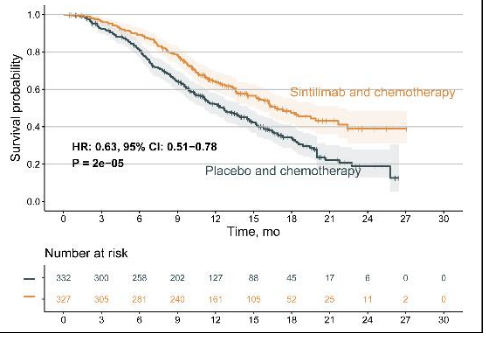

ORIENT-15, overall survival, Overall, TPS ≥ 1%	The original figure may be found in the primary trial manuscript<br>DOI: 10.1136/bmj-2021-068714<br>Fig 2B	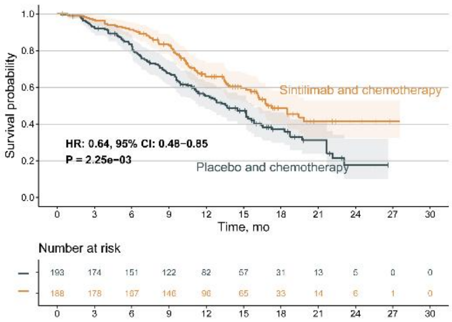
ORIENT-15, progression-free survival, Overall	The original figure may be found in the primary trial manuscript<br>DOI: 10.1136/bmj-2021-068714<br>Fig 4A	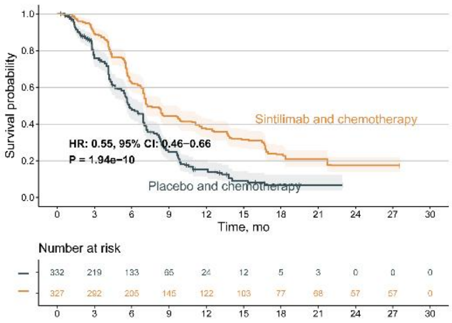
ORIENT-15, progression-free survival, TPS ≥ 1%	The original figure may be found in the primary trial manuscript<br>DOI: 10.1136/bmj-2021-068714<br>Fig 4B	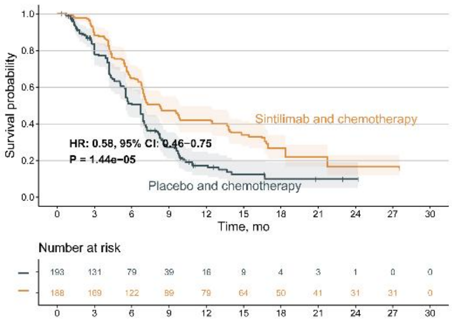
NEPTUNE, overall survival, IPS < 1%	The original figure may be found in the primary trial manuscript<br>DOI: 10.1016/j.jtho.2022.09.223<br>Fig S3C	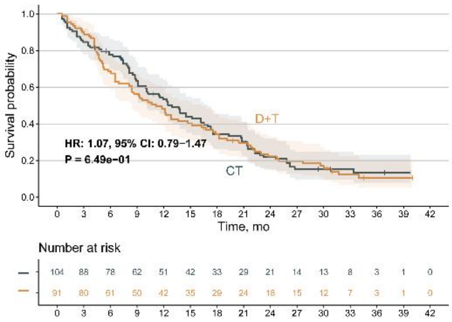

Table S5. Summary of HR of overall, PD-L1 ≥ 1%, and PD-L1 < 1% populations.

Studies	Study number	HR of OS (95% CI)			HR of PFS (95% CI)		
		Total	PD-L1 ≥ 1%	PD-L1 < 1%	Total	PD-L1 ≥ 1%	PD-L1 < 1%
Paz-Ares et al. (2018) [3]	KEYNOTE-407	0.64 (0.49-0.85)	0.65 (0.45-0.92)	0.61 (0.38-0.98)	0.56 (0.45-0.70)	0.49 (0.38-0.65)	0.68 (0.47-0.98)
Socinski et al. (2018, 2021) [4,5]	IMpower150	0.80 (0.68-0.95)	0.73 (0.57-0.94)	0.90 (0.71-1.14)	0.61 (0.52-0.72)	0.50 (0.39-0.64)	0.77 (0.61-0.99)
Motzer et al. (2018) [6]	CheckMate 214	0.66 (0.53-0.82)	0.45 (0.29-0.71)	0.73 (0.56-0.96)			
Rini et al. (2019) [7]	IMmotion151	0.91	0.85	1.01	0.84	0.73	0.93

		(0.76-1.08)	(0.64-1.13)	(0.81-1.24)	(0.72-0.99)	(0.57-0.94)	(0.77-1.15)
Choueiri, et al. (2020) [9]	JAVELIN Renal 101	0.80	0.86	0.72	0.69	0.64	0.84
		(0.62-1.03)	(0.62-1.19)	(0.45-1.17)	(0.57-0.83)	(0.51-0.81)	(0.60-1.17)
Emens et al. (2021) [16,17]	IMpassion130	0.88	0.69	1.05	0.86	0.71	0.99
		(0.76-1.03)	(0.54-0.88)	(0.87-1.28)	(0.73-1.02)	(0.54-0.94)	(0.80-1.23)
Janjigian et al. (2021,	CheckMate 649	0.79	0.76	0.92	0.77	0.75	0.93
2022) [18,19]		(0.70-0.89)	(0.67-0.87)	(0.70-1.23)	(0.68-0.87)	(0.65-0.85)	(0.69-1.26)
		0.90	0.86	1.05			
		(0.77-1.04)	(0.72-1.01)	(0.75-1.46)			
Liu et al. (2021) [20]	IMpower133	0.76	0.87	0.51	0.77	0.86	0.52
		(0.60-0.95)	(0.51-1.50)	(0.30-0.89)	(0.63-0.95)	(0.51-1.46)	(0.31-0.88)
Luo et al. (2021) [21]	ESCORT-1st	0.70	0.59	0.79	0.56	0.51	0.62
		(0.56-0.88)	(0.43-0.80)	(0.57-1.11)	(0.46-0.68)	(0.39-0.67)	(0.46-0.83)
Mai et al. (2021) [22]	NCT03581786				0.52	0.59	0.35
					(0.36-0.74)	(0.39-0.89)	(0.15-0.81)
Miles et al. (2021) [23]	IMpassion131	1.12	1.11	1.14	0.86	0.82	1.02
		(0.88-1.43)	(0.76-1.64)	(0.84-1.55)	(0.70-1.05)	(0.60-1.12)	(0.78-1.33)
Moehler et al. (2021)[24]	JAVELIN Gastric 100	0.91	0.72	1.00	1.04	0.87	1.14
		(0.74-1.11)	(0.49-1.05)	(0.79-1.27)	(0.85-1.28)	(0.60-1.27)	(0.89-1.45)
Monk et al. (2021) [25]	JAVELIN Ovarian 100				1.43	1.04	0.95
					(1.05-1.95)	(0.70-1.56)	(0.63-1.43)
					1.14	0.92	0.86
					(0.83-1.56)	(0.61-1.37)	(0.55-1.33)
Moore et al. (2021)[26]	IMagyn050/GOG	0.96	0.98	1.03	0.92	0.80	1.08
	3015/ENGOT -OV39	(0.74-1.26)	(0.68-1.41)	(0.70-1.50)	(0.79-1.07)	(0.65-0.99)	(0.86-1.35)
Nishio et al. (2021) [27]	IMpower132	0.86	0.97	0.67	0.60	0.68	0.45
		(0.71-1.06)	(0.68-1.39)	(0.46-0.96)	(0.49-0.72)	(0.49-0.95)	(0.31-0.64)
Owonikoko et al. (2021)	CheckMate 451	0.92	0.95	0.65	0.72	0.65	0.72
[28]		(0.75-1.12)	(0.60-1.53)	(0.44-0.96)	(0.60-0.87)	(0.43-0.99)	(0.50-1.05)
		0.84	0.84	0.67	0.67	0.67	0.63
		(0.69-1.02)	(0.54-1.32)	(0.46-0.99)	(0.56-0.81)	(0.45-1.01)	(0.44-0.91)
Reck et al. (2021)[29]	CheckMate 9LA	0.72	0.70	0.67	0.67	0.67	0.68
		(0.61-0.86)	(0.56-0.89)	(0.51-0.88)	(0.56-0.79)	(0.53-0.84)	(0.51-0.89)
Rodriguez-Abreu et al.	KEYNOTE-189	0.56	0.63	0.51	0.49	0.42	0.67
(2021)[30]		(0.46-0.69)	(0.48-0.73)	(0.36-0.71)	(0.41-0.59)	(0.33-0.53)	(0.49-0.93)
Sugawara et al.	TASUKI-52				0.57	0.58	0.55
(2021)[31]					(0.46-0.72)	(0.43-0.78)	(0.38-0.78)
Zhou et al. (2021)[32]	CameL				0.60	0.56	0.76
					(0.45-0.79)	(0.39-0.82)	(0.45-1.26)
Sun et al. (2021) [35]	KEYNOTE-590	0.73	0.62	0.86	0.65	0.51	0.80
		(0.62-0.86)	(0.49-0.78)	(0.68-1.10)	(0.55-0.76)	(0.41-0.65)	(0.64-1.10)
Burtness et al. (2022)[36]	KEYNOTE-048	0.83	0.78	1.51	1.34	1.16	4.32
		(0.70-0.99)	(0.64-0.96)	(0.96-2.37)	(1.13-1.59)	(0.96-1.39)	(2.63-7.08)
		0.77	0.65	1.21	0.92	0.82	1.46
		(0.63-0.93)	(0.53-0.80)	(0.76-1.94)	(0.77-1.10)	(0.67-1.00)	(0.93-2.30)
Cortes et al. (2022) [37]	KEYNOTE-355	0.89	0.86	0.97	0.82	0.75	1.09
		(0.76-1.05)	(0.72-1.04)	(0.72-1.32)	(0.70-0.98)	(0.62-0.91)	(0.78-1.51)
Doki et al. (2022)[38]	CheckMate 648	0.74	0.54	0.98	0.81	0.65	0.95
		(0.58-0.96)	(0.37-0.80)	(0.76-1.28)	(0.64-1.04)	(0.46-0.92)	(0.73-1.24)

		0.78 (0.62-0.98)	0.64 (0.46-0.90)	0.96 (0.74-1.25)	1.26 (1.04-1.52)	1.02 (0.73-1.43)	1.45 (1.13-1.88)
Paz-Ares et al. (2022) [39]	CheckMate 227	0.72 (0.63-0.82)	0.76 (0.65-0.90)	0.64 (0.51-0.81)	0.79 (0.60-0.90)	0.81 (0.68-0.96)	0.74 (0.58-0.94)
			0.91 (0.78-1.06)	0.82 (0.65-1.02)		0.98 (0.83-1.15)	0.72 (0.57-0.91)
Peters et al. (2022) [40]	CheckMate 743	0.75 (0.63-0.90)	0.71 (0.57-0.88)	0.99 (0.69-1.43)			
Spigel et al. (2022) [41]	PACIFIC	0.72 (0.59-0.89)	0.61 (0.44-0.85)	1.15 (0.75-1.75)	0.55 (0.45-0.68)	0.47 (0.35-0.64)	0.80 (0.53-1.20)
Wolchok et al. (2022)[42]	CheckMate 067	0.52 (0.43-0.64)	0.52 (0.39-0.69)	0.55 (0.39-0.76)			
		0.63 (0.52-0.76)	0.52 (0.39-0.68)	0.78 (0.57-1.06)			
Yau et al. (2022)[43]	CheckMate 459	0.85 (0.72-1.02)	0.80 (0.54-1.19)	0.84 (0.69-1.02)	0.93 (0.79-1.10)	0.71 (0.48-1.03)	0.98 (0.81-1.17)
Zhou et al. (2022)[44]	GEMSTONE-302				0.48 (0.39-0.60)	0.46 (0.35-0.62)	0.56 (0.40-0.77)
Lu et al. (2022) [48]	ORIENT-15	0.63 (0.51-0.78)	0.71 (0.53-0.95)	0.55 (0.40-0.75)	0.56 (0.46-0.68)	0.59 (0.46-0.77)	0.52 (0.39-0.68)
Dummer et al. (2022) [53]	COMBI-i	0.79 (0.59-1.05)	1.00 (0.63-1.59)	0.57 (0.37-0.88)	0.82 (0.66-1.03)	0.84 (0.60-1.18)	0.76 (0.54-1.07)
Wu et al. (2022) [54,55]	JUPITER-06	0.58 (0.43-0.78)	0.61 (0.42-0.90)	0.63 (0.37-1.08)	0.58 (0.46-0.74)	0.59 (0.44-0.79)	0.59 (0.40-0.88)
de Castro et al. (2023) [56]	NEPTUNE	1.02 (0.87-1.19)	1.01 (0.84-1.20)	1.06 (0.78-1.45)			

OS, overall survival; PFS, progression-free survival; PDL1, programmed death-ligand 1

Figure S1. PRISMA flowchart of the study selection process.

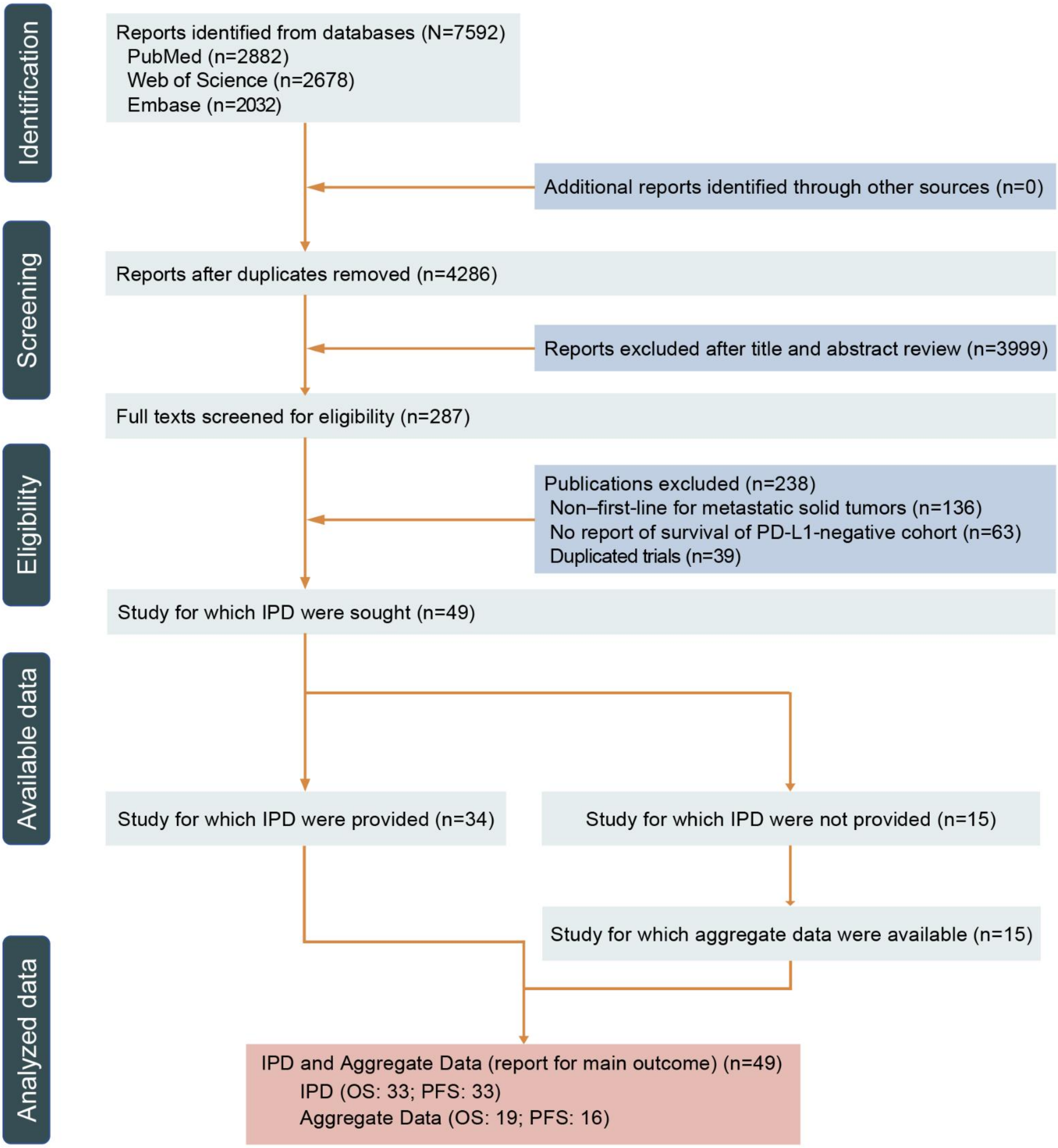

I

PD, individual patient data; KM, Kaplan–Meier; PD-L1, programmed death-ligand 1; OS, overall survival; PFS, progression-free survival.

Figure S2. Funnel plots of publication bias in trials with IPD and aggregate data.

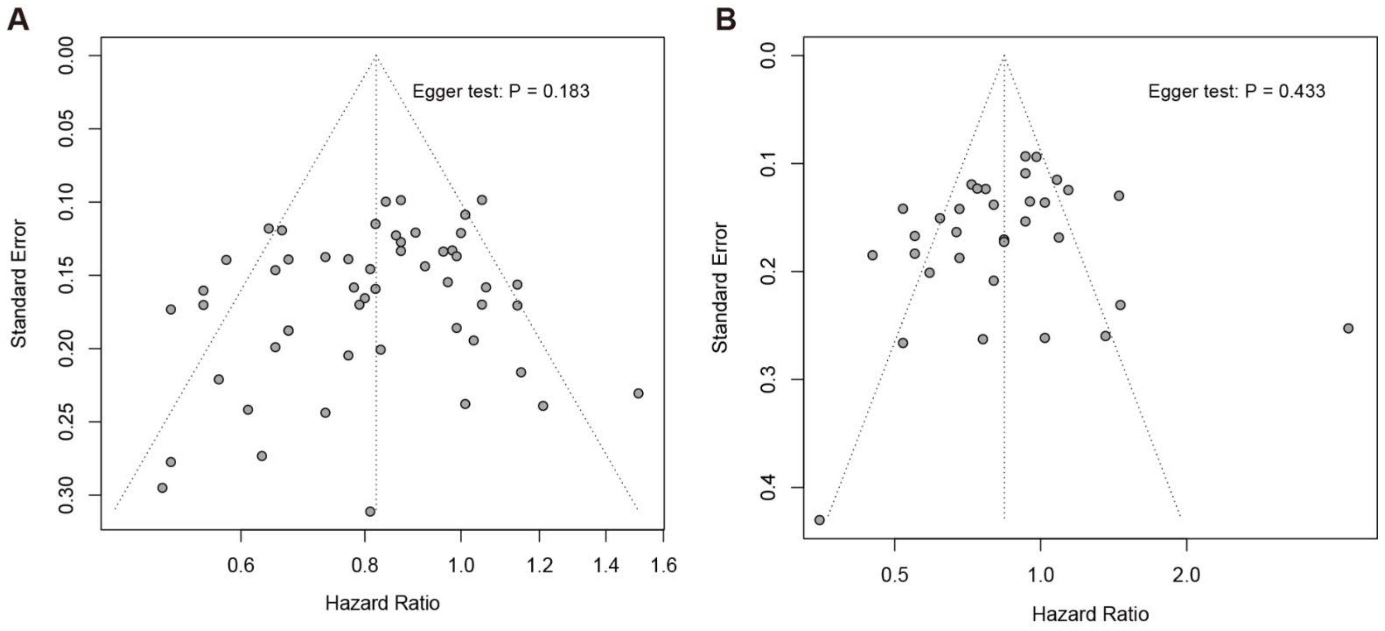

A, overall survival; B, progression-free survival.

Figure S3. Forest plots of hazard ratio of overall survival in the total populations.

Trial	Population	Number of Patient	HR	95%−CI	Weight
**Single agent**					
IMvigor130 (Atezoli)	UC	228	1.14	[0.82; 1.60]	1.8%
JAVELIN Gastric 100	GC	364	1.00	[0.79; 1.27]	2.5%
CheckMate 451 (Nivo)	SCLC	122	0.67	[0.46; 0.96]	1.6%
KEYNOTE−361	UC	341	0.87	[0.68; 1.12]	2.4%
KEYNOTE−048 (Pembro)	HNSCC	89	1.51	[0.96; 2.37]	1.3%
PACIFIC	NSCLC	148	1.15	[0.75; 1.75]	1.4%
CheckMate 067 (Nivo)	Melanoma	230	0.78	[0.57; 1.06]	2.0%
CheckMate 459	HCC	595	0.84	[0.69; 1.02]	2.9%
Random effects model			**0.93**	**[0.81; 1.07]**	**15.9%**
Heterogeneity: $I^2 = 45\%$, $\tau^2 = 0.0141$, $p = 0.08$					
**Combination agents**					
KEYNOTE−407	NSCLC	194	0.61	[0.38; 0.98]	1.2%
IMpower150	NSCLC	340	0.90	[0.71; 1.14]	2.5%
CheckMate 214	RCC	562	0.73	[0.56; 0.96]	2.3%
IMmotion151	RCC	553	1.01	[0.81; 1.24]	2.7%
IMpower130	NSCLC	356	0.81	[0.61; 1.08]	2.1%
JAVELIN Renal 101	RCC	252	0.73	[0.45; 1.17]	1.2%
IMvigor130 (Atezoli + chemo)	UC	278	0.82	[0.60; 1.12]	2.0%
IMspire150	Melanoma	171	0.83	[0.56; 1.23]	1.5%
IMpower131	NSCLC	331	0.87	[0.67; 1.13]	2.3%
KEYNOTE−426	RCC	321	0.77	[0.52; 1.16]	1.5%
KEYNOTE−604	SCLC	174	0.80	[0.58; 1.11]	1.9%
IMpassion130	TNBC	266	1.05	[0.87; 1.28]	2.9%
CheckMate 649 (Nivo + chemo)	GC	266	0.92	[0.70; 1.23]	2.2%
CheckMate 649 (Nivo + ipi)	GC	157	1.05	[0.75; 1.46]	1.8%
IMpower133	SCLC	65	0.51	[0.30; 0.89]	1.0%
ESCORT−1st	ESCC	256	0.79	[0.57; 1.11]	1.8%
IMpassion131	TNBC	359	1.14	[0.84; 1.55]	2.0%
IMagyn050/GOG 3015/ENGOT −OV39	OC	517	1.03	[0.70; 1.50]	1.6%
IMpower132	NSCLC	163	0.67	[0.46; 0.96]	1.6%
CheckMate 451 (Nivo + ipi)	SCLC	127	0.65	[0.44; 0.96]	1.5%
CheckMate 9LA	NSCLC	264	0.67	[0.51; 0.88]	2.2%
KEYNOTE−189	NSCLC	190	0.51	[0.36; 0.71]	1.8%
CLEAR	RCC	215	0.50	[0.28; 0.89]	0.9%
KEYNOTE−590	ESCC	349	0.86	[0.68; 1.10]	2.5%
KEYNOTE−048 (Pembro + chemo)	HNSCC	82	1.21	[0.76; 1.94]	1.2%
KEYNOTE−355	TNBC	212	0.97	[0.72; 1.32]	2.0%
CheckMate 648 (Nivo + chemo)	ESCC	329	0.98	[0.76; 1.28]	2.3%
CheckMate 648 (Nivo + ipi)	ESCC	330	0.96	[0.74; 1.25]	2.3%
CheckMate 227 (Nivo + chemo)	NSCLC	363	0.82	[0.65; 1.02]	2.6%
CheckMate 227 (Nivo + ipi)	NSCLC	373	0.64	[0.51; 0.81]	2.6%
CheckMate 743	MPM	135	0.99	[0.69; 1.43]	1.7%
CheckMate 067 (Nivo + ipi)	Melanoma	236	0.55	[0.39; 0.76]	1.8%
IMbrave150	HCC	77	0.81	[0.44; 1.49]	0.8%
EMPOWER−Lung 3	NSCLC	139	1.01	[0.63; 1.60]	1.2%
ATTRACTION−4	GC	610	0.87	[0.72; 1.06]	2.9%
ORIENT−15	ESCC	297	0.55	[0.40; 0.75]	2.0%
CheckMate 9ER	RCC	472	0.65	[0.49; 0.87]	2.1%
CAPSTONE−1	SCLC	396	0.66	[0.52; 0.83]	2.5%
ASTRUM−005	SCLC	469	0.58	[0.44; 0.76]	2.2%
POSEIDON (Durva + treme + chemo)	NSCLC	255	0.77	[0.58; 1.00]	2.2%
POSEIDON (Durva + chemo)	NSCLC	243	0.99	[0.76; 1.30]	2.3%
COMBI−i	Melanoma	213	0.57	[0.37; 0.88]	1.3%
JUPITER−06	ESCC	193	0.63	[0.37; 1.08]	1.0%
NEPTUNE	NSCLC	195	1.06	[0.78; 1.45]	2.0%
Random effects model			**0.80**	**[0.75; 0.86]**	**84.1%**
Heterogeneity: $I^2 = 50\%$, $\tau^2 = 0.0233$, $p < 0.001$					
**Random effects model**			**0.82**	**[0.77; 0.87]**	**100.0%**
Heterogeneity: $I^2 = 50\%$, $\tau^2 = 0.0233$, $p < 0.001$					
Test for subgroup differences: $\chi^2_1 = 3.85$, df = 1 ($p = 0.05$)					

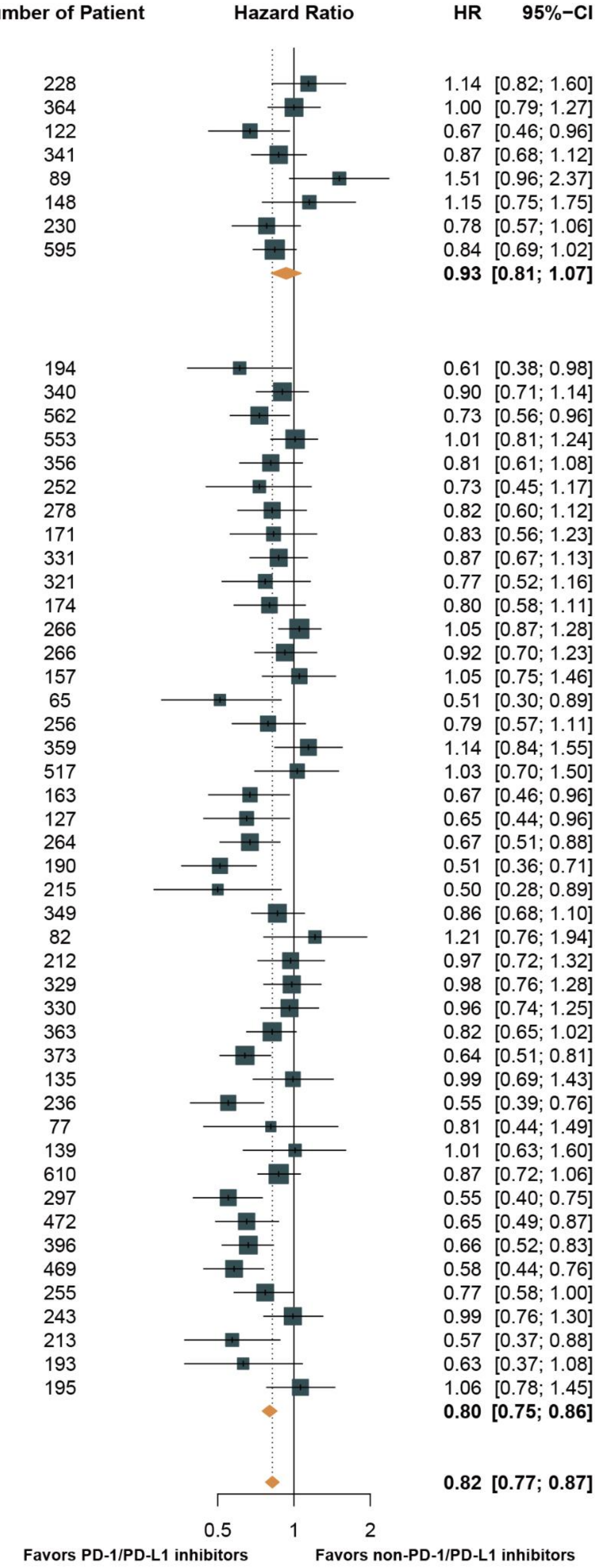

The therapy effects are calculated with random effects models. PD-1, programmed death-1; PD-L1, programmed death-ligand 1; HR, hazard ratio; CI, confidence interval.

Figure S4. Forest plots of hazard ratio of progression-free survival in the total population.

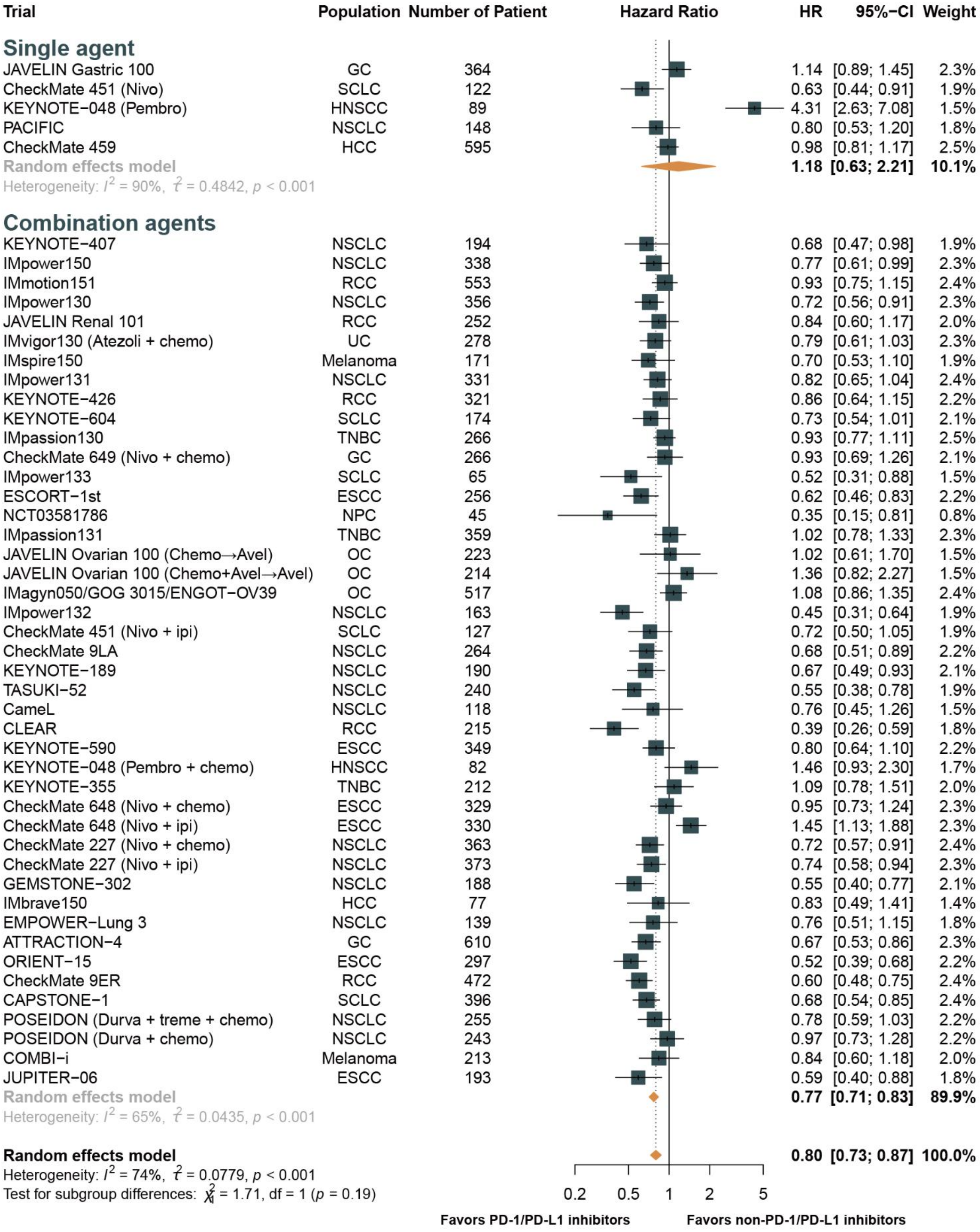

The therapy effects are calculated with random effects models. PD-1, programmed death-1; PD-L1, programmed death-ligand 1; HR, hazard ratio; CI, confidence interval.

Figure S5. Comparisons of hazard ratio between trials with IPD and trials of IPD and aggregate data

Subgoup	Number of Cohort	Number of Patients	HR
**OS: single agent**			
Trial with IPD	7	1889	0.91 (0.79 to 1.04)
Trial with IPD and aggregate data	8	2177	0.93 (0.81 to 1.07)
Subgroup difference: P = 0.828			
**OS: combination agents**			
Trial with IPD	26	6913	0.80 (0.73 to 0.88)
Trial with IPD and aggregate data	44	12343	0.80 (0.75 to 0.86)
Subgroup difference: P = 1.000			
**PFS: single agent**			
Trial with IPD	4	1196	1.38 (0.67 to 2.82)
Trial with IPD and aggregate data	5	1318	1.18 (0.63 to 2.21)
Subgroup difference: P = 0.726			
**PFS: combination agents**			
Trial with IPD	29	7450	0.79 (0.71 to 0.89)
Trial with IPD and aggregate data	44	11615	0.77 (0.71 to 0.83)
Subgroup difference: P = 0.714			

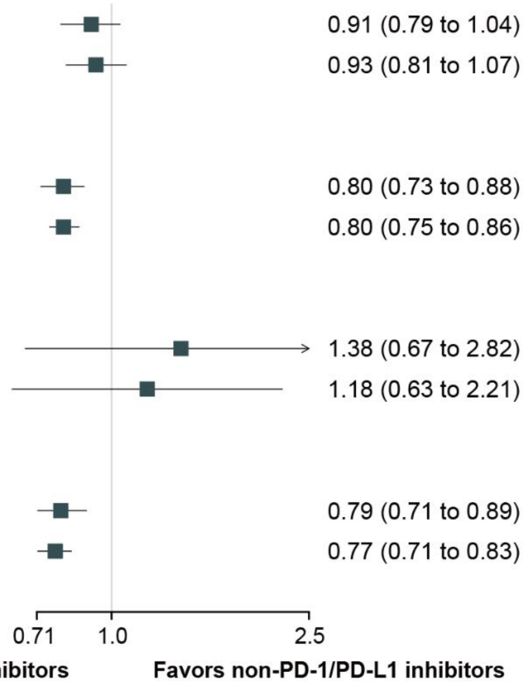

IPD, individual patient data; OS, overall survival; PFS, progression-free survival; PD-1, rogrammed death-1; PD-L1, programmed death-ligand 1; HR, hazard ratio.

Figure S6. Summary of the limits of error of each trial for KMSubtraction process.

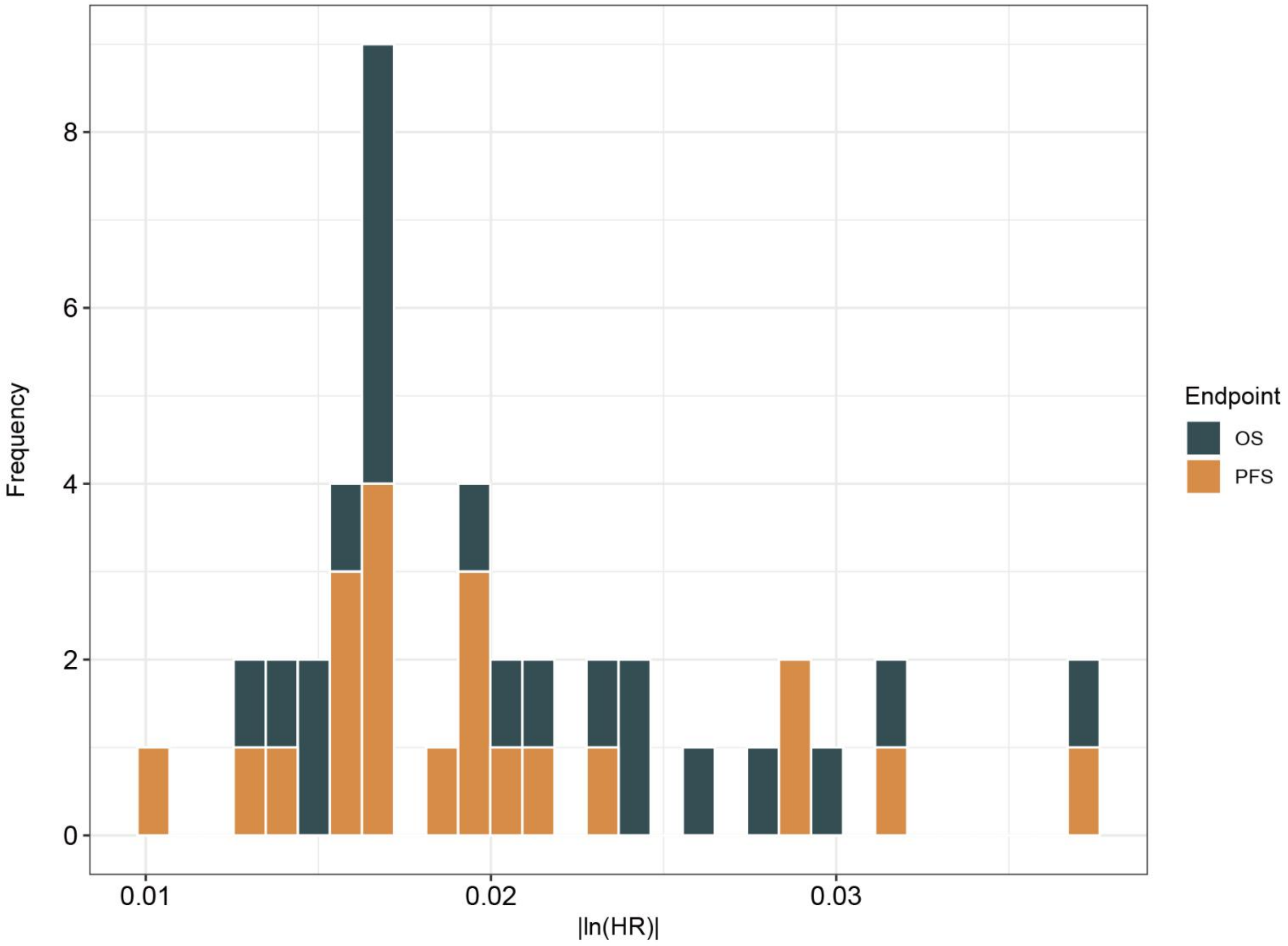

Figure S7. Correlation of reported and reconstructed survival outcomes.

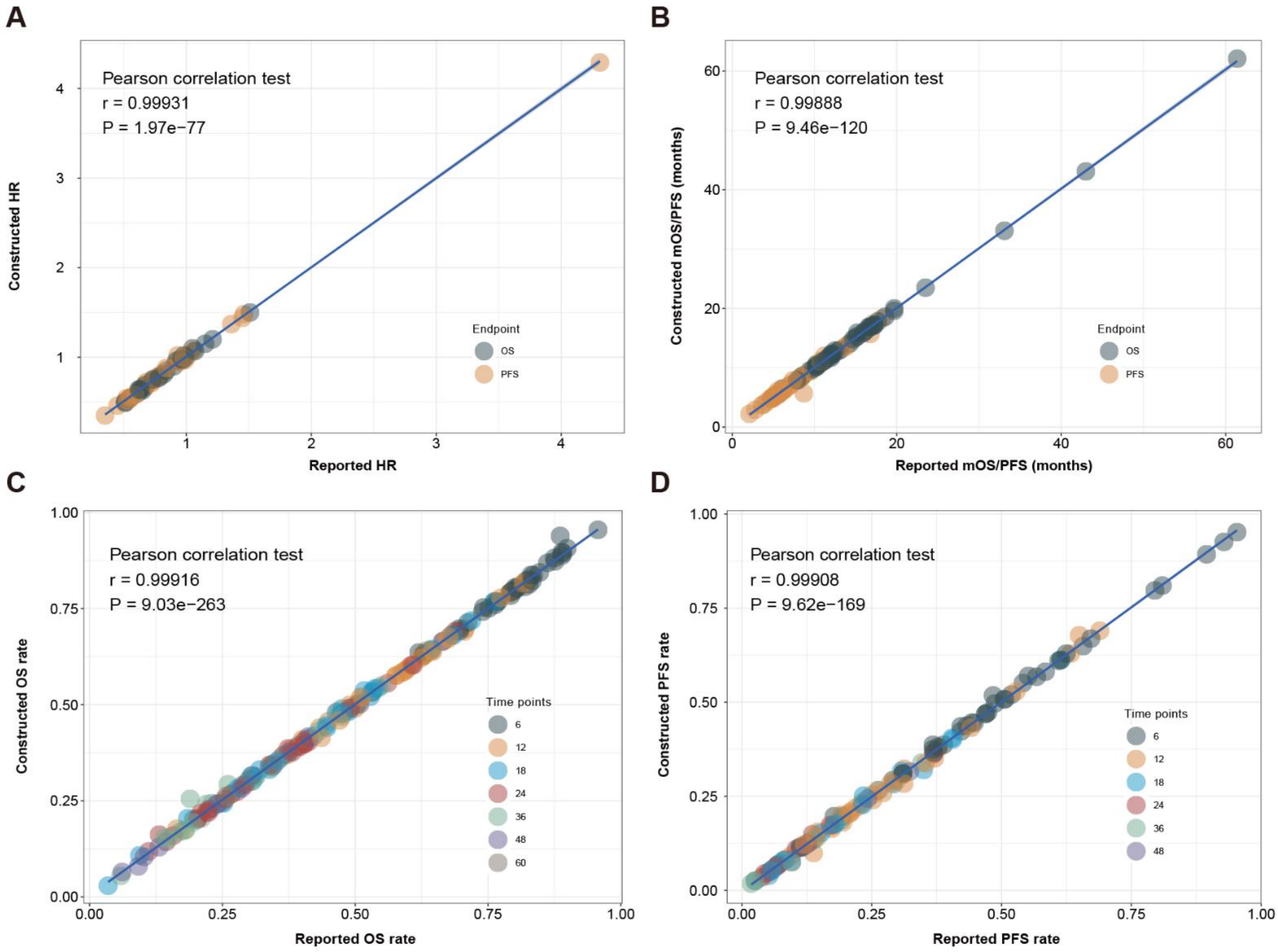

(A), Hazard ratio; (B), median survival time, (C), overall survival (OS) rate and (D), progression-free survival (PFS) rate at different time points.

Figure S8. Funnel plots of publication bias in trials with IPD.

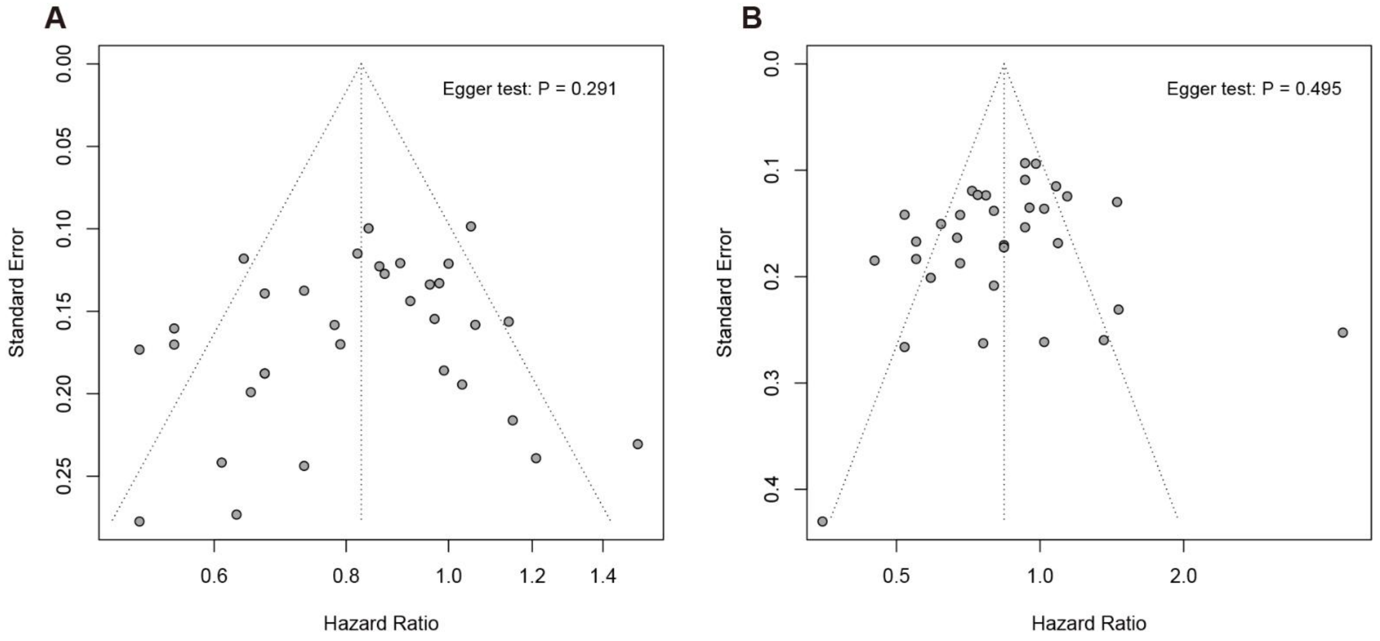

Figure S9. Kaplan–Meier estimates of overall survival treated with single agents, stratified by cancer type.

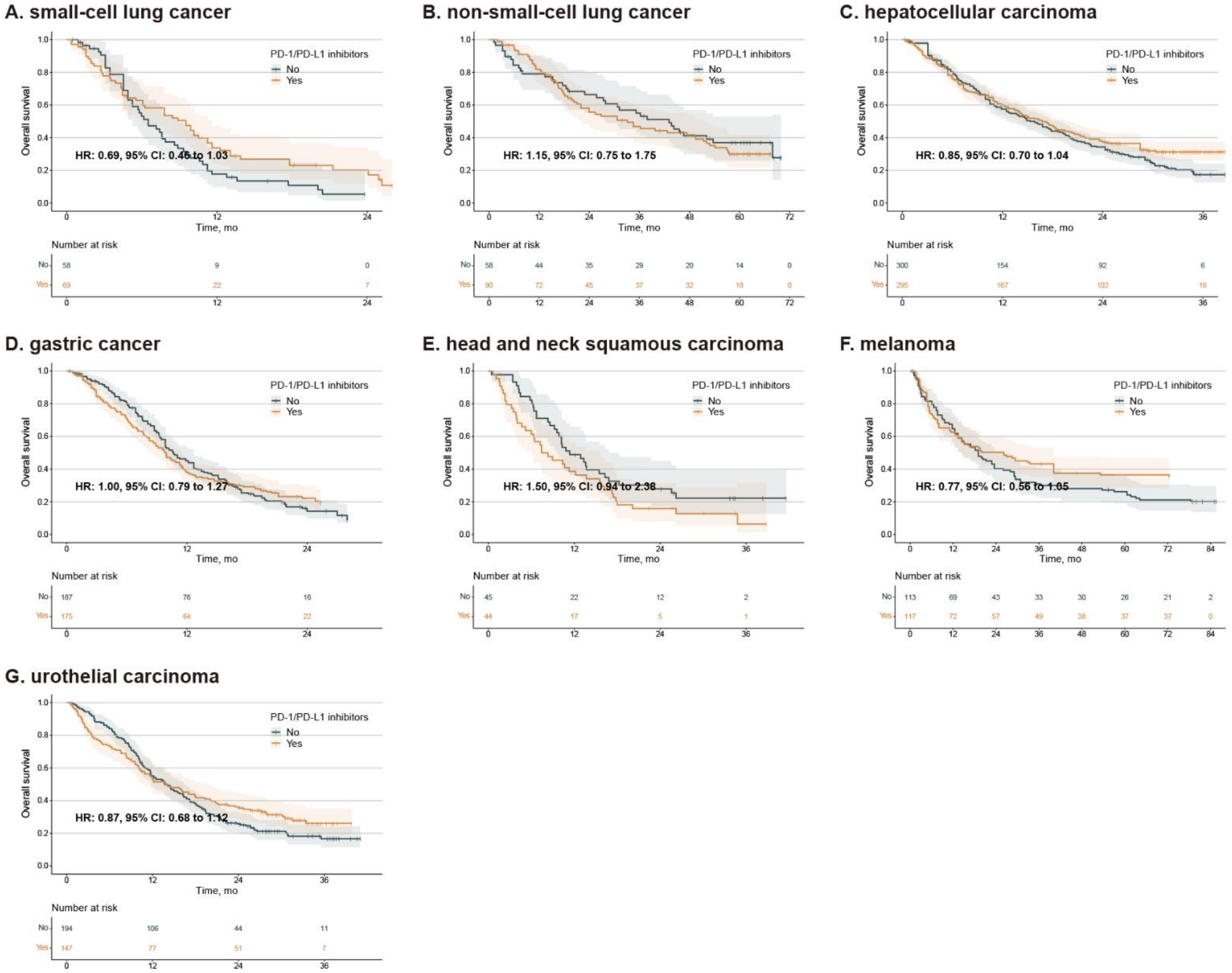

Overall survival in the PD-L1 < 1% population with small-cell lung cancer (A), non-small-cell lung cancer (B), hepatocellular carcinoma (C), gastric cancer (D), head and neck squamous cell carcinoma (E), melanoma (F), and urothelial carcinoma (G). PD-1, programmed death-1; PD-L1, programmed death-ligand 1; HR, hazard ratio; CI, confidence interval.

Figure S10. Forest plots of hazard ratio of overall survival treated with combination agents.

Subgoup	Number of Cohort	Number of Patients (%)	Median OS (mo),exp	Median OS (mo),ctr	HR
**Population**					
NSCLC	7	1896 (24.3)	16.1	12.4	0.74 (0.67 to 0.82)
ESCC	5	1571 (20.2)	12.9	11.6	0.83 (0.73 to 0.95)
TNBC	3	1103 (14.2)	17.6	18.6	1.10 (0.95 to 1.28)
RCC	3	1441 (18.5)	33.9	31.6	0.86 (0.73 to 1.01)
SCLC	2	187 (2.4)	9.2	7.1	0.59 (0.43 to 0.81)
MPM	1	135 (1.7)	17.2	16.9	1.00 (0.69 to 1.46)
OC	1	517 (6.6)	30.9	NA	1.03 (0.70 to 1.50)
GC	1	285 (3.7)	13.8	12.8	0.96 (0.72 to 1.27)
HNSCC	1	82 (1.1)	11.5	10.7	1.20 (0.75 to 1.94)
Melanoma	1	236 (3.0)	62.2	19.1	0.53 (0.38 to 0.74)
UC	1	341 (4.4)	14.0	13.7	0.87 (0.68 to 1.12)
**Drug**					
Nivolumab	8	2651 (34.0)	18.2	14.6	0.83 (0.76 to 0.91)
Atezolizumab	7	2530 (32.5)	23.3	22.5	1.00 (0.91 to 1.11)
Pembrolizumab	6	1365 (17.5)	14.1	11.8	0.82 (0.73 to 0.93)
Camrelizumab	1	256 (3.3)	15.1	12.3	0.79 (0.57 to 1.11)
Avelumab	1	326 (4.2)	NA	NA	0.64 (0.41 to 0.99)
Toripalimab	1	193 (2.5)	NA	11.6	0.63 (0.37 to 1.07)
Durvalumab	1	195 (2.5)	11.1	12.9	1.07 (0.79 to 1.47)
Sintilimab	1	278 (3.6)	15.3	11.2	0.61 (0.45 to 0.85)
**PD-1/PD-L1 inhibitor**					
PD−1	17	4743 (60.9)	16.3	13.2	0.80 (0.75 to 0.86)
PD−L1	9	3051 (39.1)	23.3	22.2	0.98 (0.89 to 1.08)
**Control**					
Chemotherapy	21	5995 (76.9)	16.7	14.5	0.87 (0.82 to 0.93)
Targeted therapy	3	1441 (18.5)	33.9	31.6	0.86 (0.73 to 1.01)
Placebo	1	122 (1.6)	8.6	6.3	0.63 (0.43 to 0.94)
Ipilimumab	1	236 (3.0)	62.2	19.1	0.53 (0.38 to 0.74)
**PD-L1 clone**					
28-8	9	2907 (37.3)	17.7	14.4	0.82 (0.75 to 0.90)
22C3	7	1643 (21.1)	14.1	11.8	0.79 (0.70 to 0.89)
SP142	6	2465 (31.6)	24.0	23.4	1.02 (0.92 to 1.14)
SP263	3	586 (7.5)	21.8	17.3	0.81 (0.64 to 1.01)
JS311	1	193 (2.5)	NA	11.6	0.63 (0.37 to 1.07)
**IHC scoring method**					
TPS	13	4043 (51.9)	17.4	13.7	0.78 (0.72 to 0.85)
IPS	6	2483 (31.9)	25.1	26.2	1.04 (0.93 to 1.16)
CPS	4	700 (9.0)	13.6	11.3	0.89 (0.75 to 1.06)
TPS&IPS	3	568 (7.3)	15.7	12.5	0.79 (0.65 to 0.96)

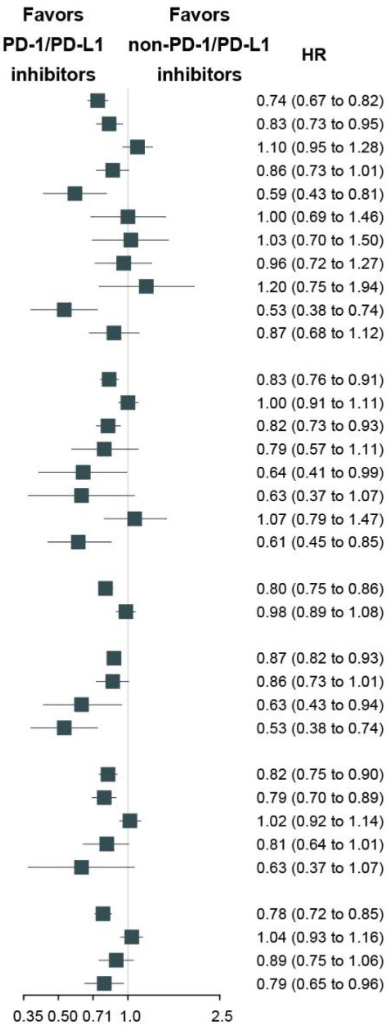

PD-1, programmed death-1; PD-L1, programmed death-ligand 1; NSCLC, non-small-cell lung cancer; ESCC, esophageal squamous cell carcinoma; TNBC, triple-negative breast cancer; RCC, renal cell carcinoma; SCLC, small-cell lung cancer; MPM, malignant pleural mesothelioma; OC, ovarian cancer; GC, gastric cancer; HNSCC, head and neck squamous cell carcinoma;UC, urothelial carcinoma; TPS, tumor cell proportion score; IPS, immune cell proportion score; CPS, combined positive score.

Figure S11. Kaplan–Meier estimates of overall survival and progression-free survival treated with combination agents, stratified by PD-1/PD-L1 agents.

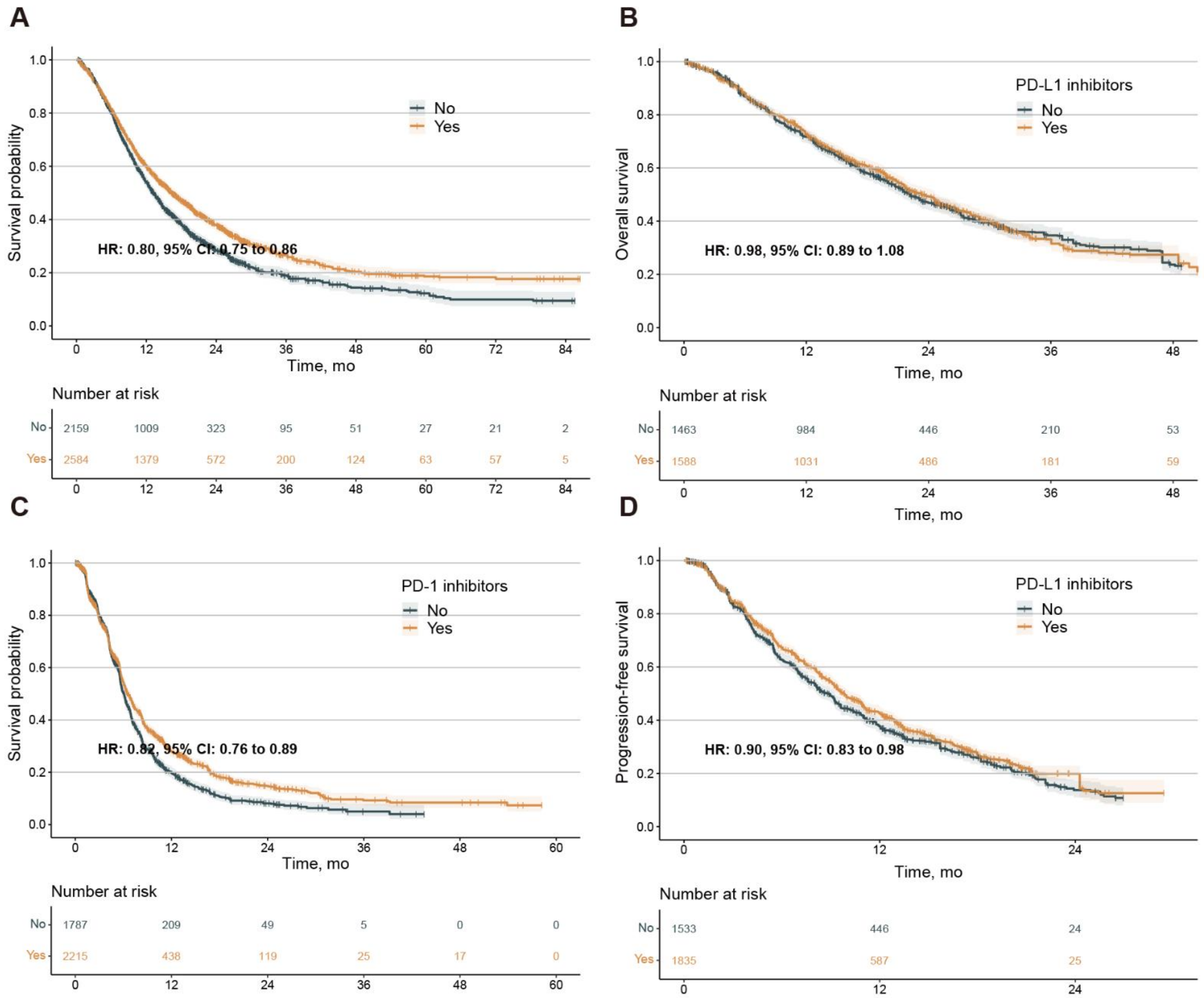

A-B, overall survival in the PD-L1 < 1% population treated with PD-1 inhibitors (A) and PD-L1 inhibitors (B). C-D, progression-free survival In the PD-L1 < 1% population treated with PD-1 inhibitors (C) and PD-L1 inhibitors (D). PD-1, programmed death-1; PD-L1, programmed death-ligand 1; HR, hazard ratio; CI, confidence interval.

Figure S12. Kaplan–Meier estimates of progression-free survival treated with single agents, stratified by cancer type.

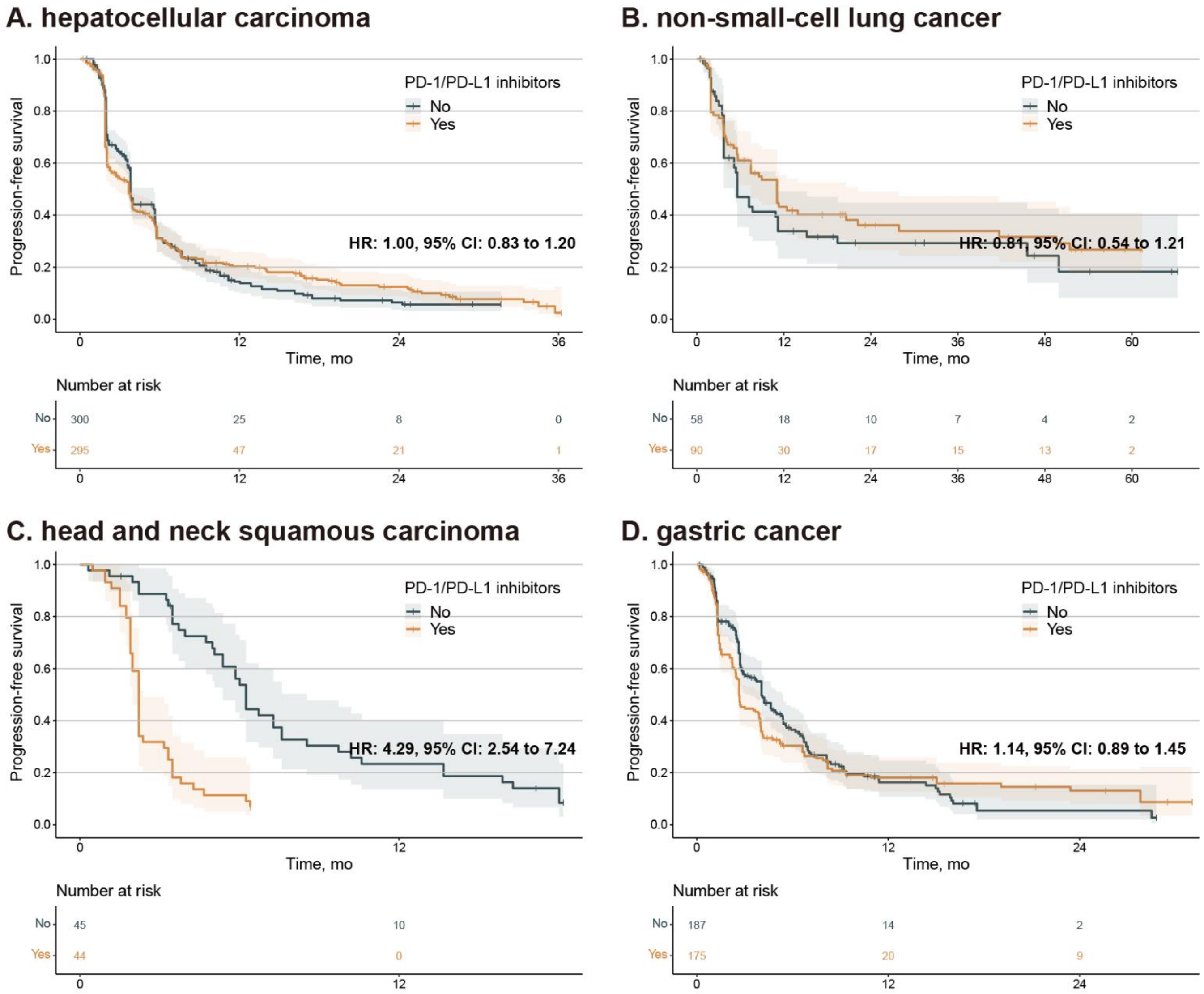

Progression-free survival in the PD-L1 < 1% population with hepatocellular carcinoma (A), non-small-cell lung cancer (B), head and neck squamous cell carcinoma (C), and gastric cancer (D). PD-1, programmed death-1; PD-L1, programmed death-ligand 1; HR, hazard ratio; CI, confidence interval.

Figure S13. Forest plots of hazard ratio of progression-free survival treated with combination agents.

Subgoup	Number of Cohort	Number of Patients (%)	Median PFS (mo),exp	Median PFS (mo),ctr	HR
**Population**					
NSCLC	9	2284 (31.0)	7.0	5.7	0.70 (0.63 to 0.77)
ESCC	5	1571 (21.3)	6.0	5.7	0.79 (0.71 to 0.89)
TNBC	3	1103 (15.0)	5.7	5.6	1.00 (0.88 to 1.14)
RCC	2	879 (11.9)	12.1	9.4	0.89 (0.75 to 1.06)
OC	2	843 (11.4)	16.8	18.2	1.13 (0.92 to 1.38)
NPC	1	45 (0.6)	11.0	6.1	0.35 (0.15 to 0.80)
HNSCC	1	82 (1.1)	4.9	6.2	1.48 (0.93 to 2.36)
GC	1	285 (3.9)	8.8	8.1	1.02 (0.75 to 1.38)
SCLC	1	65 (0.9)	5.6	4.3	0.55 (0.32 to 0.95)
Melanoma	1	213 (2.9)	12.1	10.4	0.89 (0.64 to 1.23)
**Drug**					
Atezolizumab	7	2528 (34.3)	9.2	8.3	0.95 (0.87 to 1.04)
Nivolumab	5	1836 (24.9)	6.0	6.1	0.89 (0.79 to 0.99)
Pembrolizumab	5	1024 (13.9)	6.2	5.8	0.85 (0.74 to 0.97)
Toripalimab	2	238 (3.2)	8.2	5.7	0.52 (0.37 to 0.75)
Camrelizumab	2	413 (5.6)	7.2	5.7	0.68 (0.54 to 0.86)
Avelumab	2	652 (8.8)	15.7	13.0	0.85 (0.66 to 1.08)
Sugemalimab	1	188 (2.6)	7.4	4.9	0.55 (0.39 to 0.77)
Spartalizumab	1	213 (2.9)	12.1	10.4	0.89 (0.64 to 1.23)
Sintilimab	1	278 (3.8)	7.1	5.8	0.52 (0.40 to 0.69)
**PD-1/PD-L1 inhibitor**					
PD−1	16	4002 (54.3)	6.6	6.1	0.82 (0.76 to 0.89)
PD−L1	10	3368 (45.7)	9.8	8.8	0.90 (0.83 to 0.98)
**Control**					
Chemotherapy	23	6278 (85.2)	7.5	6.7	0.84 (0.79 to 0.89)
Targeted therapy	3	1092 (14.8)	12.1	9.4	0.89 (0.76 to 1.04)
**PD-L1 clone**					
28-8	7	2305 (31.3)	6.7	6.5	0.90 (0.81 to 0.99)
22C3	7	1459 (19.8)	6.3	5.8	0.76 (0.68 to 0.86)
SP142	6	2463 (33.4)	9.3	8.8	0.96 (0.88 to 1.06)
SP263	4	905 (12.3)	11.2	9.1	0.77 (0.64 to 0.92)
JS311	2	238 (3.2)	8.2	5.7	0.52 (0.37 to 0.75)
**IHC scoring method**					
TPS	13	3567 (48.4)	6.5	5.8	0.78 (0.72 to 0.84)
TPS&IPS	5	937 (12.7)	10.4	7.0	0.61 (0.51 to 0.72)
IPS	5	2288 (31.0)	10.0	9.6	1.01 (0.91 to 1.11)
CPS	3	578 (7.8)	7.4	7.2	1.11 (0.91 to 1.35)

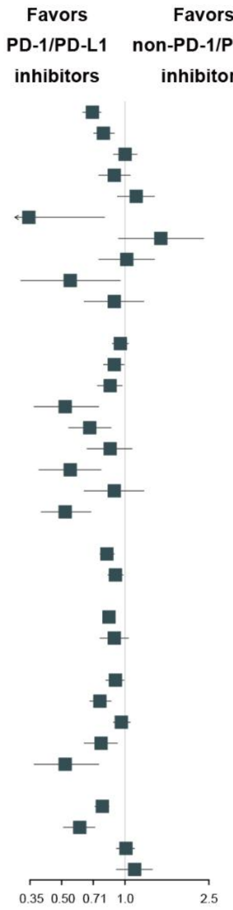

PD-1, programmed death-1; PD-L1, programmed death-ligand 1; NSCLC, non-small-cell lung cancer; ESCC, esophageal squamous cell carcinoma; TNBC, triple-negative breast cancer; RCC, renal cell carcinoma; OC, ovarian cancer; NPC, nasopharyngeal carcinoma; HNSCC, head and neck squamous cell carcinoma; GC, gastric cancer; SCLC, small-cell lung cancer; TPS, tumor cell proportion score; IPS, immune cell proportion score; CPS, combined positive score; HR, hazard ratio.

Figure S14. Survival curves estimated by Bayesian hierarchical analysis.

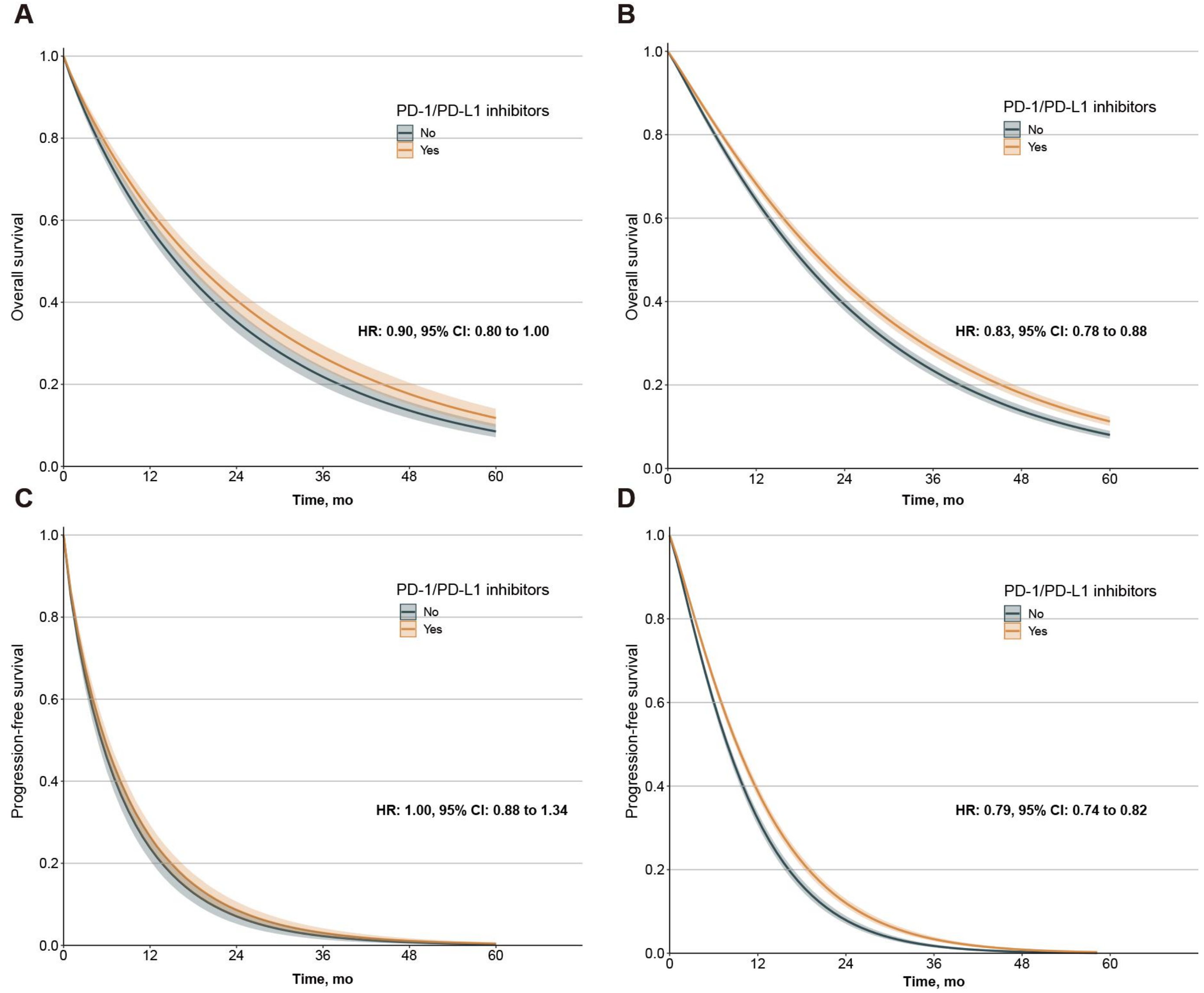

A-B, overall survival (OS) in the PD-L1 < 1% population treated with PD-1/PD-L1 single agents (A) and combination agents (B). C-D, progression-free survival (PFS) In the PD-L1 < 1% population treated with PD-1/PD-L1 single agents (C) and combination agents (D). The shadings of each curve indicate the 95% Bayesian confidence intervals for the survival proportion at the corresponding point in time of follow-up. The 2-year OS was 40.5% (37.4%-43.3%) vs. 35.3% (32.7%-38.5%) for patients treated PD-1/PD-L1 single agents or not, 43.2% (41.5%-44.9%) vs. 45.3% (43.8%-46.9%) or patients treated PD-1/PD-L1 combination agents or not. The average adjusted hazard ratio (HR) for OS was 0.90 (0.80-1.00) and 0.83 (0.78-0.88), respectively. The 1-year PFS was 26.5% (23.6%-29.4%) vs. 23.7% (20.7%-26.7%) for patients treated PD-1/PD-L1 single agents or not, 37.2% (36.0%-38.5%) vs. 30.9% (29.5%-32.5%) or patients treated PD-1/PD-L1 combination agents or not. The average adjusted HR for PFS was 1.00 (0.88-1.34) and 0.75 (0.66-0.84), respectively. PD-1, programmed death-1; PD-L1, programmed death-ligand 1.

Figure S15. Overall survival curves estimated by bayesian hierarchical analysis treated with single agents, stratified by cancer type.

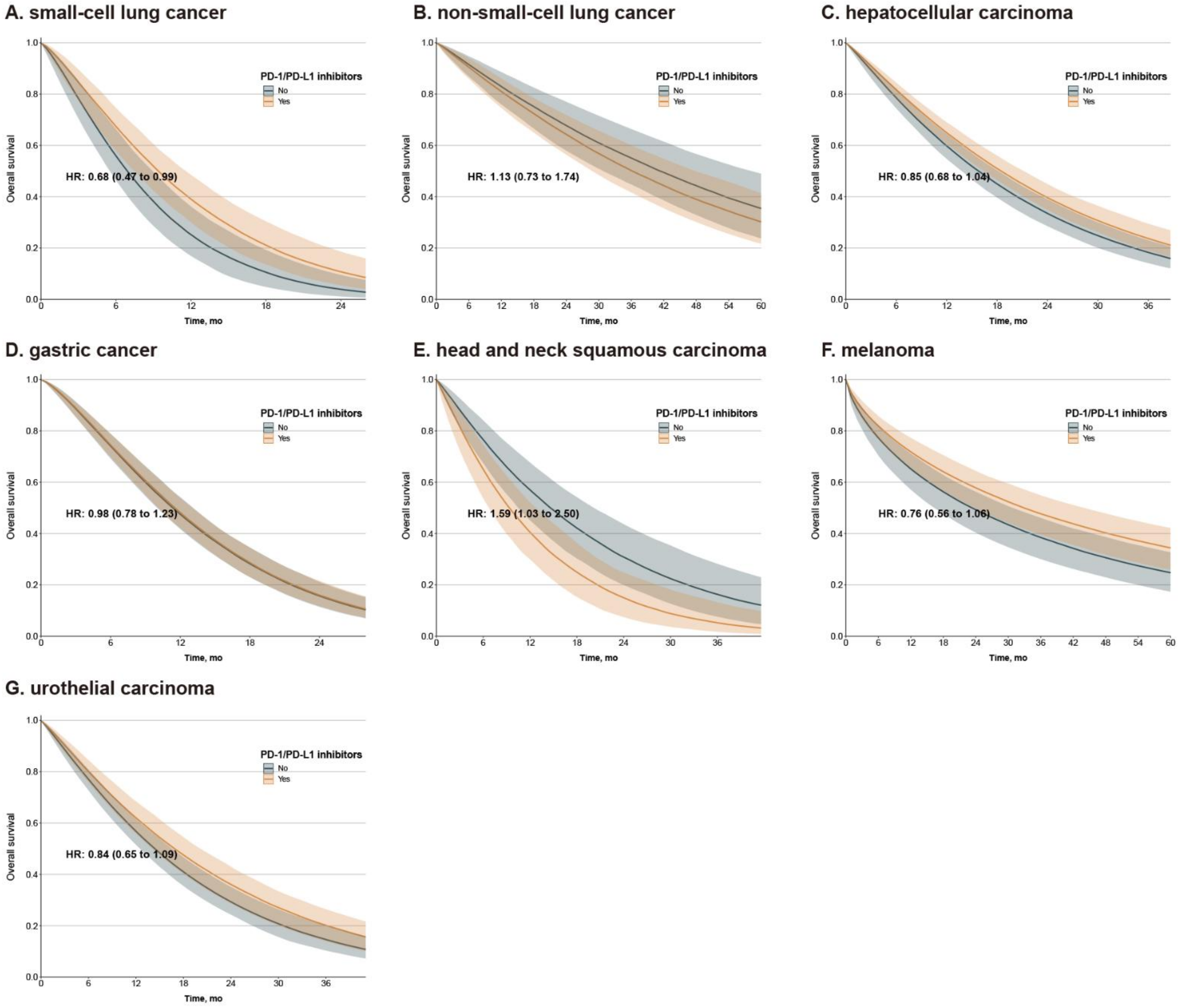

Overall survival in the PD-L1 < 1% population with small-cell lung cancer (A), non-small-cell lung cancer (B), hepatocellular carcinoma (C), gastric cancer (D), head and neck squamous cell carcinoma (E), melanoma (F), and urothelial carcinoma (G). PD-1, programmed death-1; PD-L1, programmed death-ligand 1; HR, hazard ratio; CI, confidence interval.

Figure S16. Progression-free survival curves estimated by bayesian hierarchical analysis treated with single agents, stratified by cancer type.

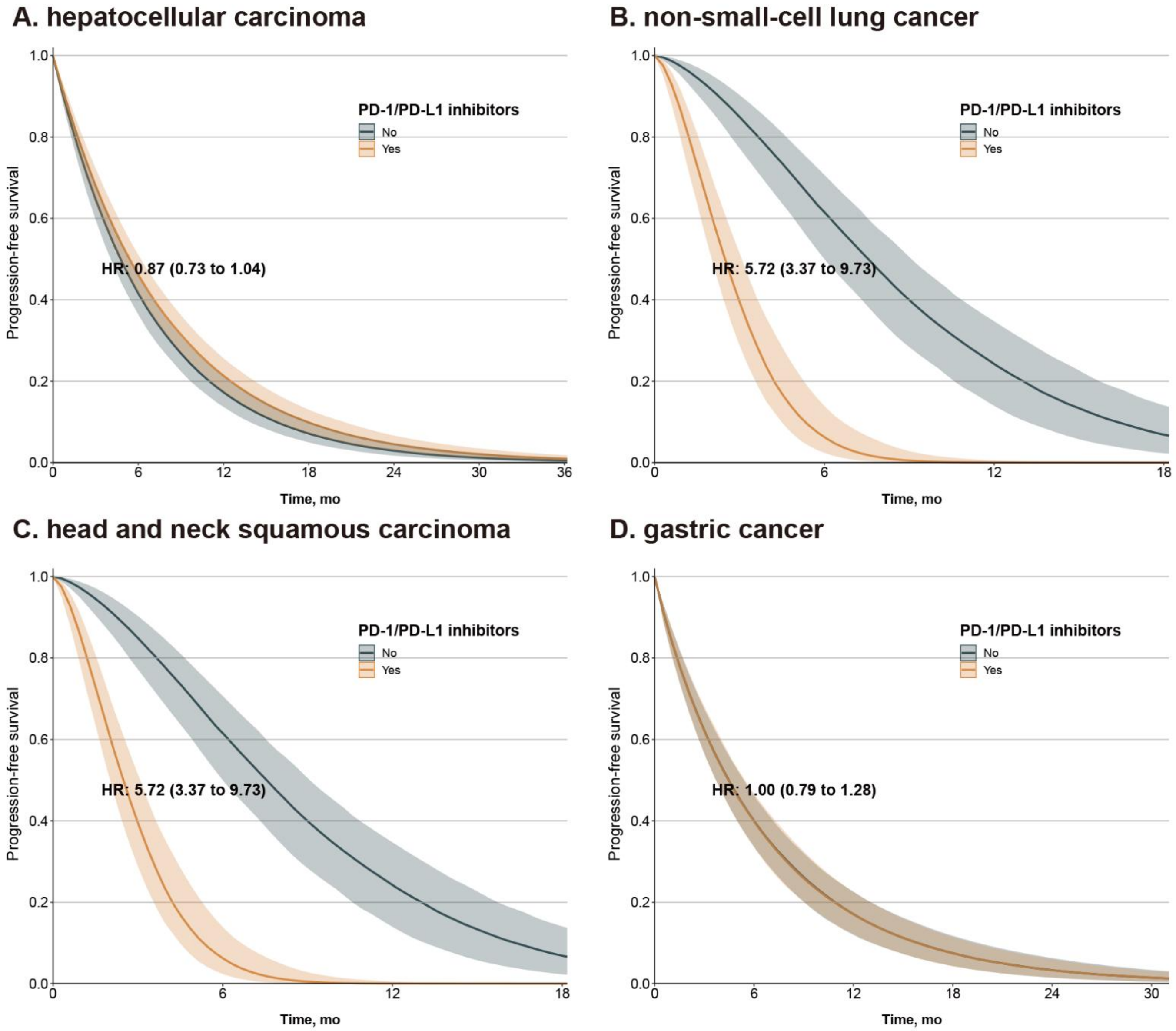

Progression-free survival in the PD-L1 < 1% population with hepatocellular carcinoma (A), non-small-cell lung cancer (B), head and neck squamous cell carcinoma (C), and gastric cancer (D). PD-1, programmed death-1; PD-L1, programmed death-ligand 1; HR, hazard ratio; CI, confidence interval.

Figure S17. Overall survival curves estimated by bayesian hierarchical analysis treated with combination agents, stratified by cancer type.

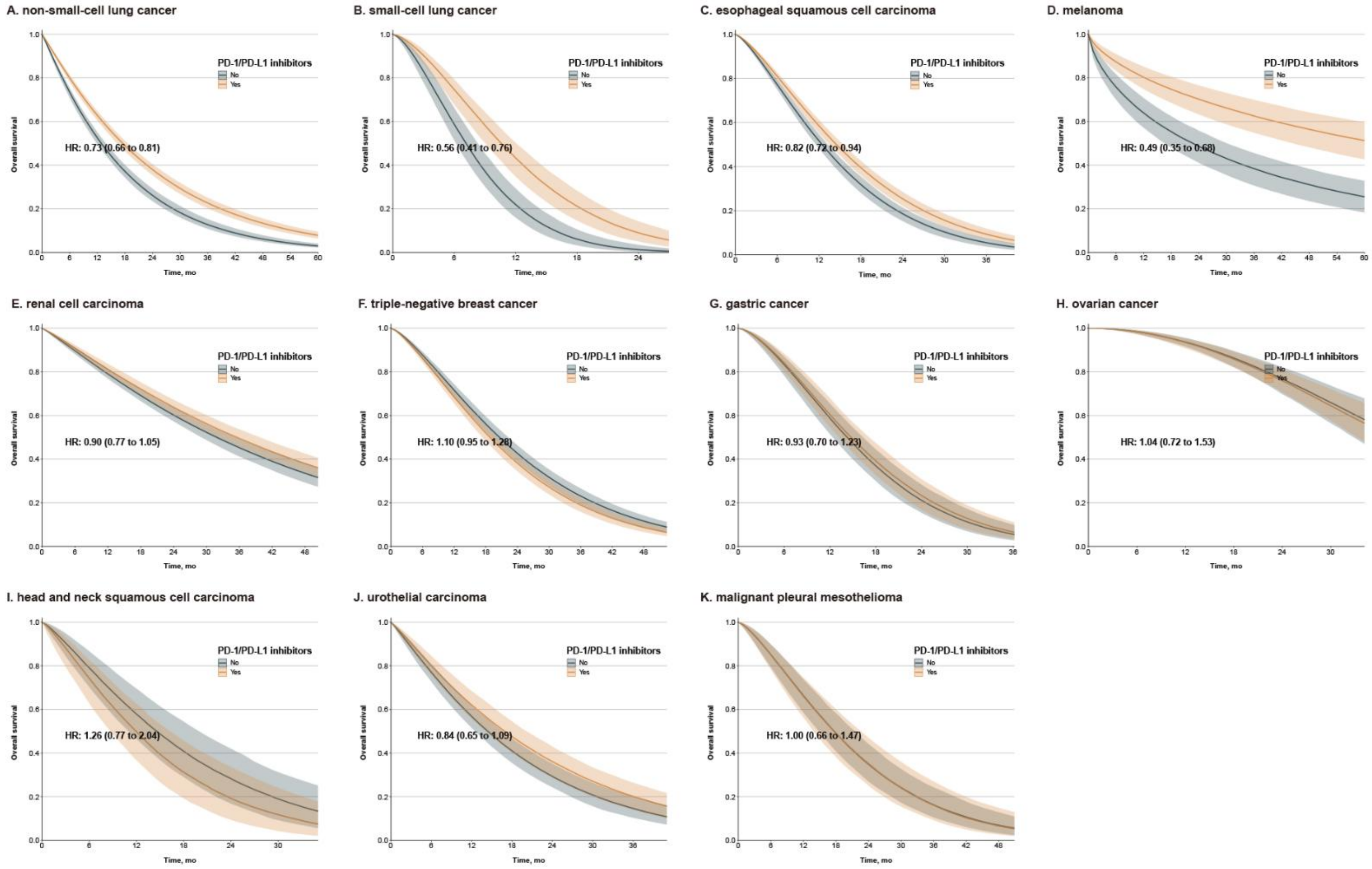

Overall survival in the PD-L1 < 1% population with non-small-cell lung cancer (A), small-cell lung cancer (B), esophageal squamous cell carcinoma (C), melanoma (D), renal cell carcinoma (E), triple-negative breast cancer (F), gastric cancer (G), ovarian cancer (H), head and neck squamous cell carcinoma (I), urothelial carcinoma (J), and malignant pleural mesothelioma (K). PD-1, programmed death-1; PD-L1, programmed death-ligand 1; HR, hazard ratio; CI, confidence interval.

Figure S18. Progression-free survival curves estimated by bayesian hierarchical analysis treated with combination agents, stratified by cancer type.

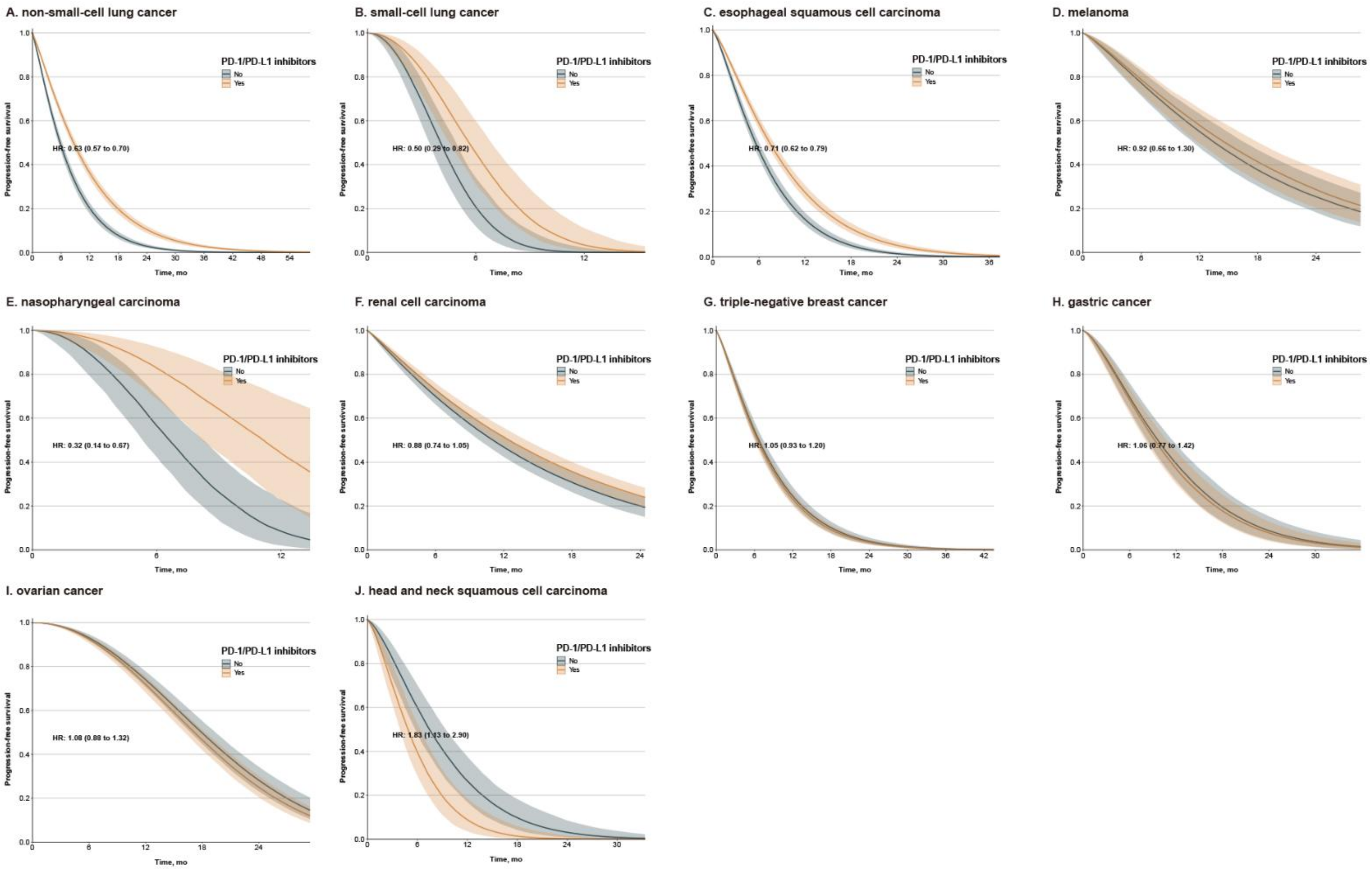

Progression-free survival in the PD-L1 < 1% population with non-small-cell lung cancer (A), small-cell lung cancer (B), esophageal squamous cell carcinoma (C), melanoma (D), nasopharyngeal carcinoma (E), renal cell carcinoma (F), triple-negative breast cancer (G), gastric cancer (H), ovarian cancer (I), and head and neck squamous cell carcinoma (J). PD-1, programmed death-1; PD-L1, programmed death-ligand 1; HR, hazard ratio; CI, confidence interval.

Figure S19. Restricted mean survival time (RMST) difference.

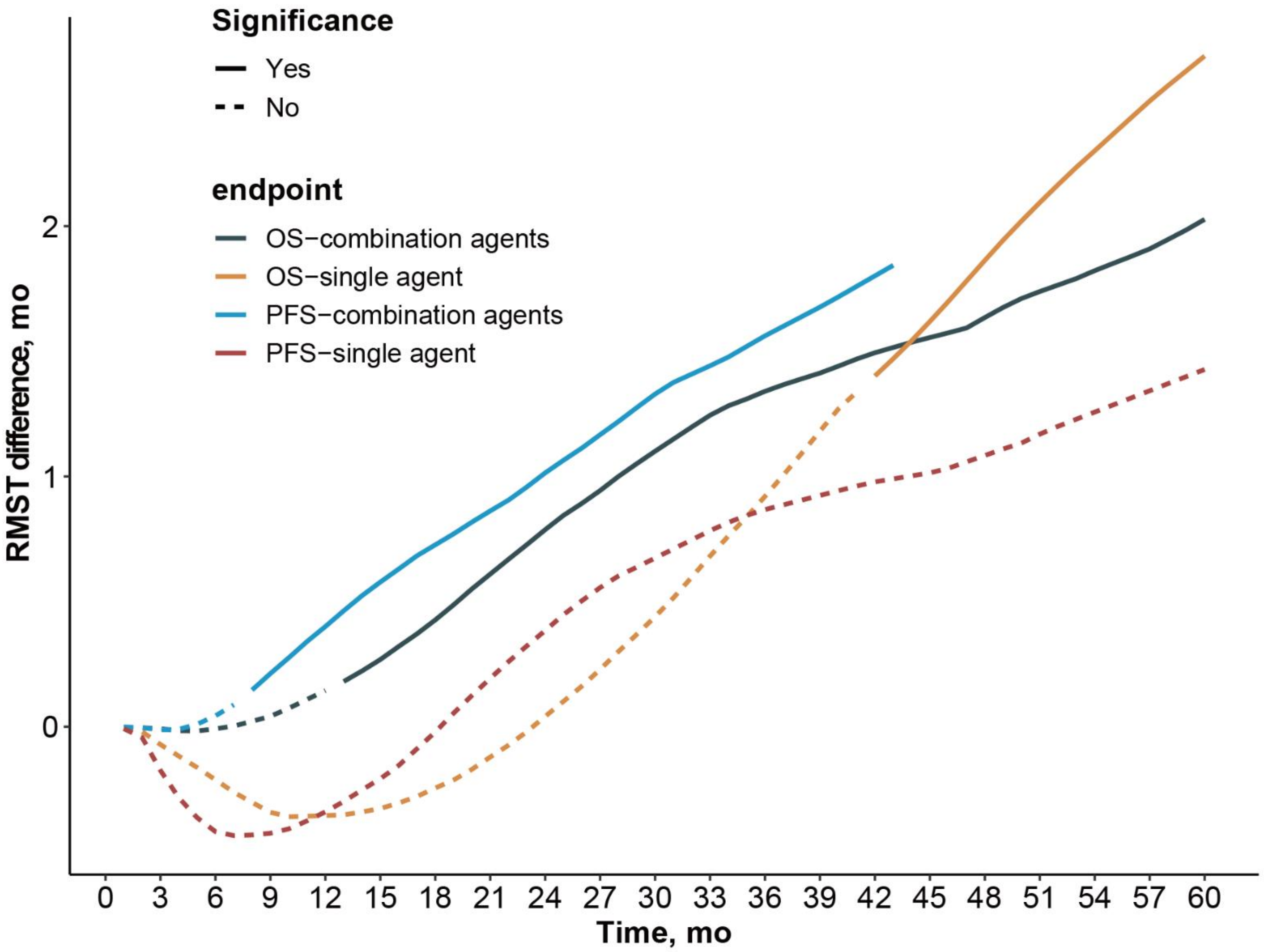

Overall survival (OS) and progression-free survival (PFS) in the PDL1 < 1% population, stratified by treatment strategy. The RMST difference for each trial was calculated by subtracting the RMST for the control arm from the experimental arm at the different truncation time points. The full parts of the lines indicate the favor for PD-1/PD-L1 inhibitor treatment with significant differences.

Figure S20. Restricted mean survival time (RMST) difference in each trial.

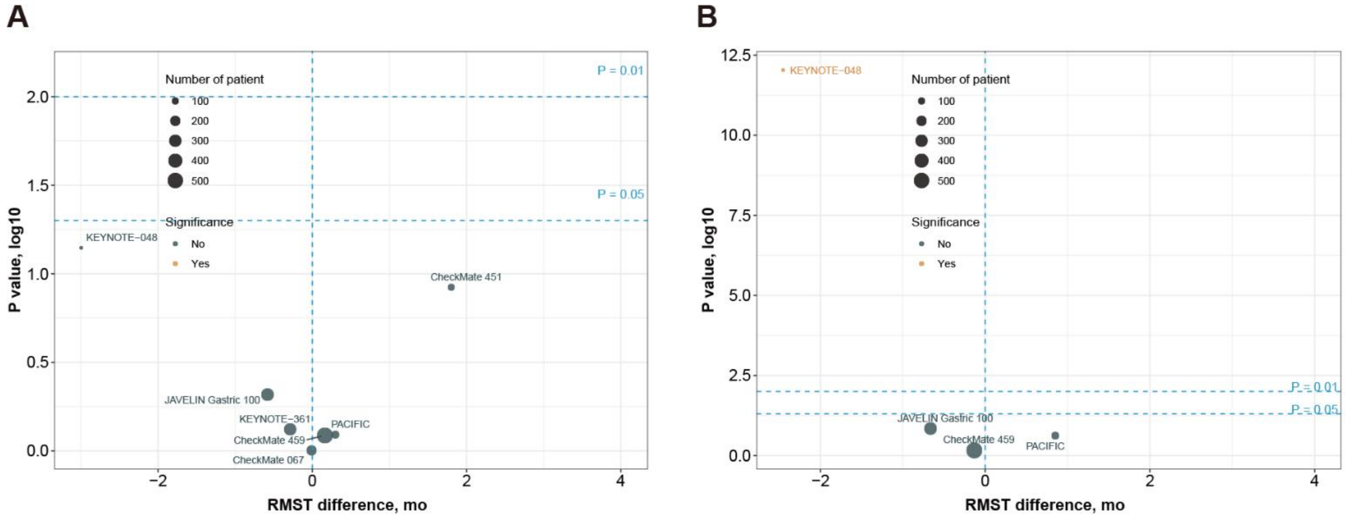

Overall survival (OS) and progression-free survival (PFS) (B) in the PD-L1 < 1% population trial treated with single agents. The RMST difference for each trial was calculated by subtracting the RMST for the control arm from the experimental arm at truncation times of 2 years for OS and 1 year for PFS. A RMST difference > 0 favors PD-1/PD-L1 inhibitor treatment.

Figure S21. Restricted mean survival time (RMST) difference in each trial.

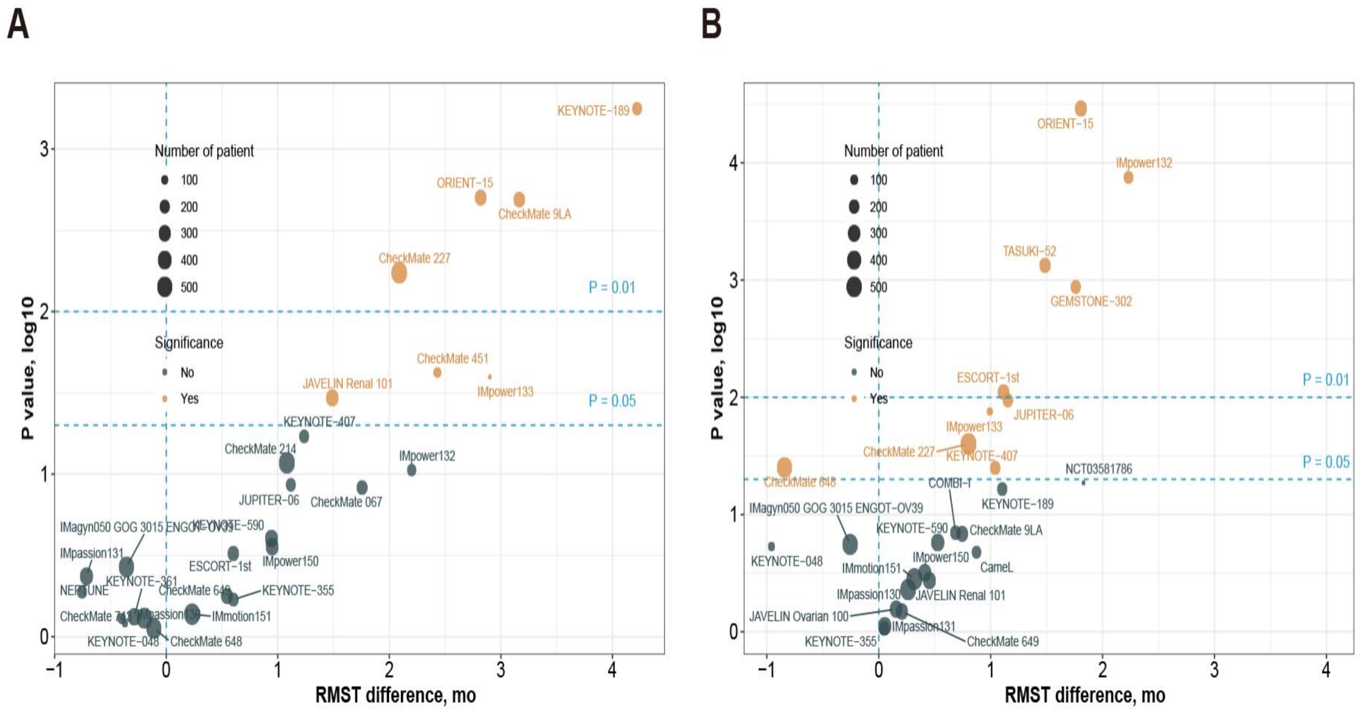

Overall survival (OS) and progression-free survival (PFS) (B) in the PD-L1 < 1% population trial treated with combination agents. The RMST difference for each trial was calculated by subtracting the RMST for the control arm from the experimental arm at truncation times of 2 years for OS and 1 year for PFS. A RMST difference > 0 favors PD-1/PD-L1 inhibitor treatment.

Figure S22. Predictive value of PD-L1 expression in each trial.

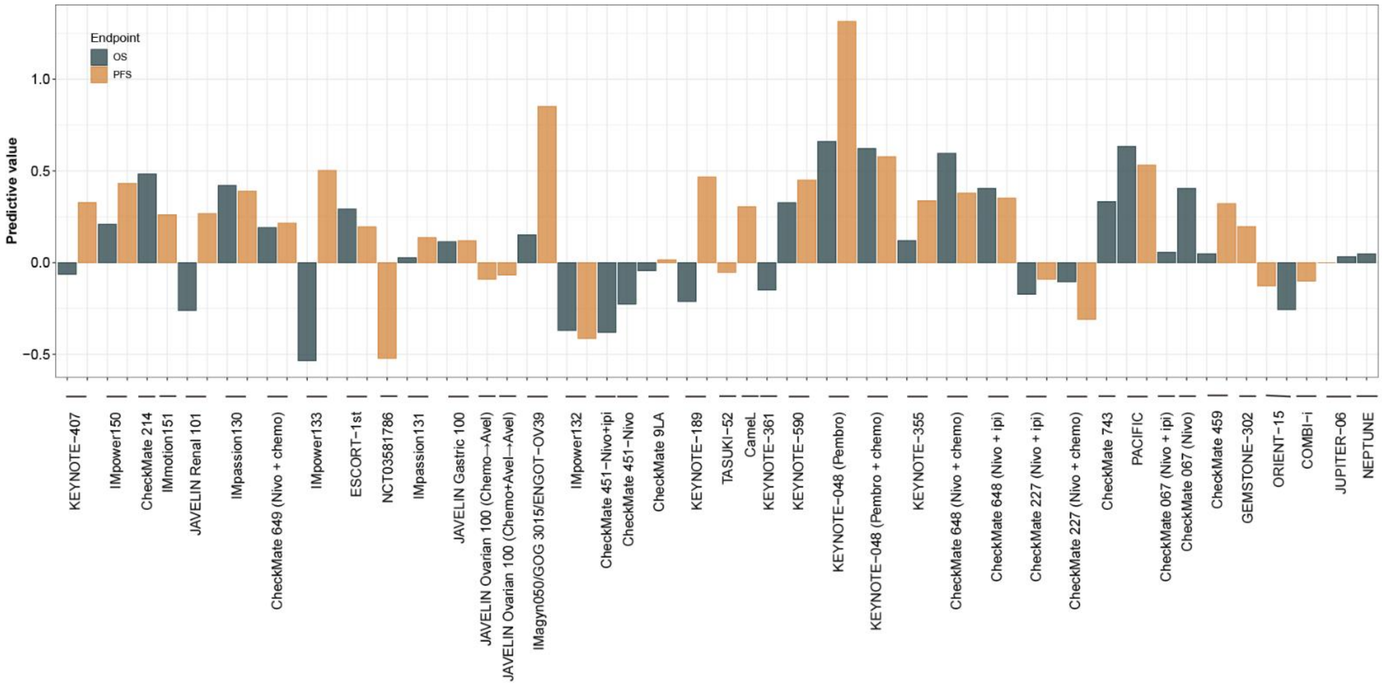

The predictive value of PD-L1 expression, defined as log transformation of the ratio of HR of PDL1 < 1% versus ≥ 1% population.

Figure S23. Predictive value of PD-L1 expression for subgroups.

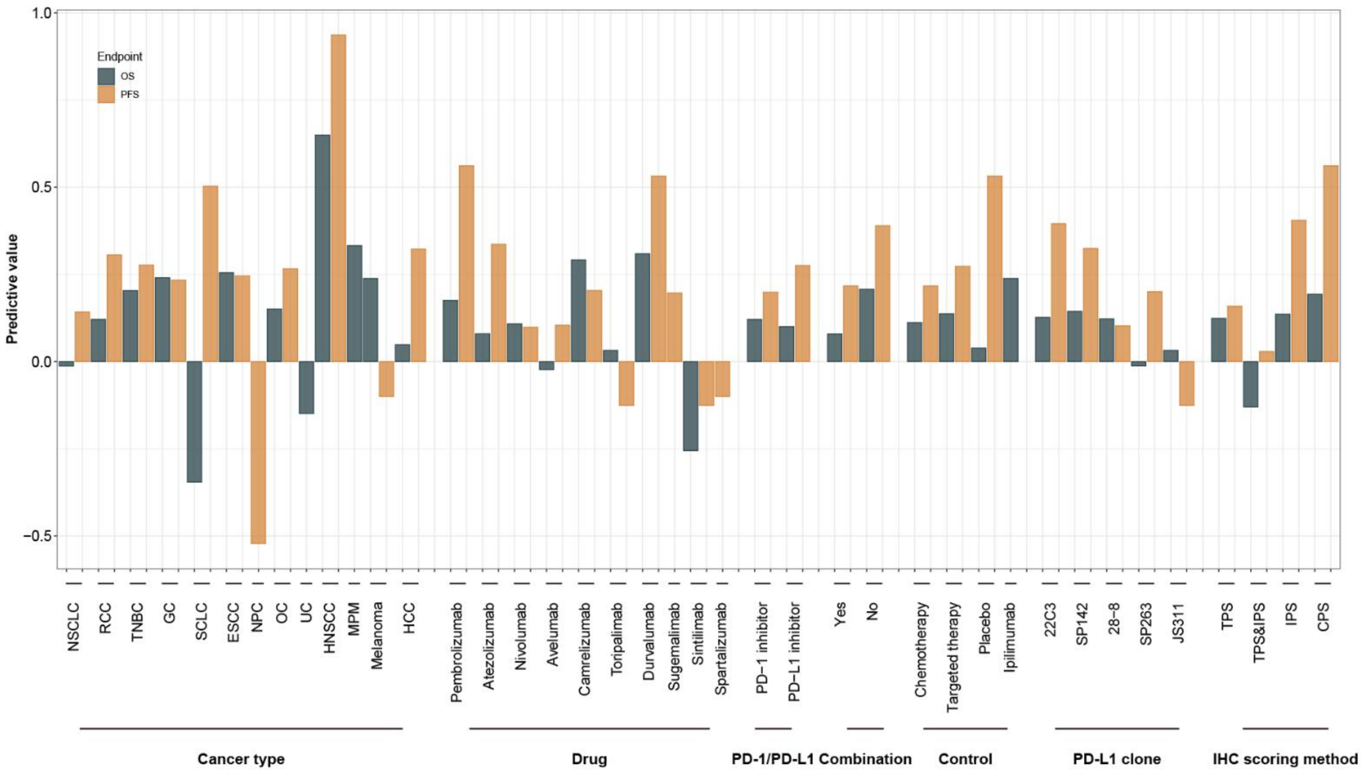

The predictive value of PD-L1 expression, defined as log transformation of the ratio of HR of PD-L1 < 1% versus ≥ 1% population. NSCLC, non-small cell lung cancer; RCC, renal cell carcinoma; UC, urothelial cancer; SCLC, small-cell lung cancer; TNBC, triple-negative breast cancer; GC, gastric cancer; ESCC, esophageal squamous cell carcinoma; NPC, nasopharyngeal carcinoma; OC, ovarian cancer; HNSCC, head and neck squamous cell carcinoma; MPM, malignant pleural mesothelioma; HCC, hepatocellular carcinoma; IHC, immunohistochemical; TPS, tumor cell proportion score; CPS, combined positive score; IPS, immune cell proportion score; PD-L1, programmed death-ligand 1

REFERENCES

1. Cherny NI, Dafni U, Bogaerts J, et al. ESMO-Magnitude of Clinical Benefit Scale version 1.1. *Ann Oncol.* 2017;28(10):2340-2366.
2. Korn EL, Allegra CJ, Freidlin B. Clinical Benefit Scales and Trial Design: Some Statistical Issues. *J Natl Cancer Inst.* 2022;114(9):1222-1227.
3. Paz-Ares L, Luft A, Vicente D, et al. Pembrolizumab plus Chemotherapy for Squamous Non-Small-Cell Lung Cancer. *The New England journal of medicine.* 2018;379(21):2040-2051.
4. Socinski MA, Jotte RM, Cappuzzo F, et al. Atezolizumab for First-Line Treatment of Metastatic Nonsquamous NSCLC. *The New England journal of medicine.* 2018;378(24):2288-2301.
5. Socinski MA, Nishio M, Jotte RM, et al. IMpower150 Final Overall Survival Analyses for Atezolizumab Plus Bevacizumab and Chemotherapy in First-Line Metastatic Nonsquamous NSCLC. *J Thorac Oncol.* 2021;16(11):1909-1924.
6. Motzer RJ, Tannir NM, McDermott DF, et al. Nivolumab plus Ipilimumab versus Sunitinib in Advanced Renal-Cell Carcinoma. *The New England journal of medicine.* 2018;378(14):1277-1290.
7. Rini BI, Powles T, Atkins MB, et al. Atezolizumab plus bevacizumab versus sunitinib in patients with previously untreated metastatic renal cell carcinoma (IMmotion151): a multicentre, open-label, phase 3, randomised controlled trial. *Lancet.* 2019;393(10189):2404-2415.
8. West H, McCleod M, Hussein M, et al. Atezolizumab in combination with carboplatin plus nab-paclitaxel chemotherapy compared with chemotherapy alone as first-line treatment for metastatic non-squamous non-small-cell lung cancer (IMpower130): a multicentre, randomised, open-label, phase 3 trial. *The Lancet Oncology.* 2019;20(7):924-937.
9. Choueiri TK, Motzer RJ, Rini BI, et al. Updated efficacy results from the JAVELIN Renal 101 trial: first-line avelumab plus axitinib versus sunitinib in patients with advanced renal cell carcinoma. *Ann Oncol.* 2020;31(8):1030-1039.
10. Galsky MD, Arija JAA, Bamias A, et al. Atezolizumab with or without chemotherapy in metastatic urothelial cancer (IMvigor130): a multicentre, randomised, placebo-controlled phase 3 trial. *Lancet.* 2020;395(10236):1547-1557.
11. Gutzmer R, Stroyakovskiy D, Gogas H, et al. Atezolizumab, vemurafenib, and cobimetinib as first-line treatment for unresectable advanced BRAF(V600) mutation-positive melanoma (IMspire150): primary analysis of the randomised, double-blind, placebo-controlled, phase 3 trial. *Lancet.* 2020;395(10240):1835-1844.
12. Ascierto PA, Stroyakovskiy D, Gogas H, et al. Overall survival with first-line atezolizumab in combination with vemurafenib and cobimetinib in BRAF(V600) mutation-positive advanced melanoma (IMspire150): second interim analysis of a multicentre, randomised, phase 3 study. *The Lancet Oncology.* 2023;24(1):33-44.
13. Jotte R, Cappuzzo F, Vynnychenko I, et al. Atezolizumab in Combination With Carboplatin and Nab-Paclitaxel in Advanced Squamous NSCLC (IMpower131): Results From a Randomized Phase III Trial. *J Thorac Oncol.* 2020;15(8):1351-1360.
14. Powles T, Plimack ER, Soulieres D, et al. Pembrolizumab plus axitinib versus sunitinib monotherapy as first-line treatment of advanced renal cell carcinoma (KEYNOTE-426): extended follow-up from a randomised, open-label, phase 3 trial. *The Lancet Oncology.* 2020;21(12):1563-1573.
15. Rudin CM, Awad MM, Navarro A, et al. Pembrolizumab or Placebo Plus Etoposide and Platinum as First-Line Therapy for Extensive-Stage Small-Cell Lung Cancer: Randomized, Double-Blind, Phase III KEYNOTE-604 Study. *Journal of clinical oncology : official journal of the American Society of Clinical Oncology.* 2020;38(21):2369-2379.
16. Emens LA, Adams S, Barrios CH, et al. First-line atezolizumab plus nab-paclitaxel for unresectable, locally advanced, or metastatic triple-negative breast cancer: IMpassion130 final overall survival analysis. *Ann Oncol.* 2021;32(8):983-993.
17. Schmid P, Rugo HS, Adams S, et al. Atezolizumab plus nab-paclitaxel as first-line treatment for unresectable, locally advanced or metastatic triple-negative breast cancer (IMpassion130): updated efficacy results from a randomised, double-blind, placebo-controlled, phase 3 trial. *The Lancet Oncology.* 2020;21(1):44-59.
18. Shitara K, Ajani JA, Moehler M, et al. Nivolumab plus chemotherapy or ipilimumab in gastro-oesophageal cancer. *Nature.* 2022;603(7903):942-948.
19. Janjigian YY, Shitara K, Moehler M, et al. First-line nivolumab plus chemotherapy versus chemotherapy alone for advanced gastric, gastro-oesophageal junction, and oesophageal adenocarcinoma (CheckMate 649): a randomised, open-label, phase 3 trial. *Lancet.* 2021;398(10294):27-40.
20. Liu SV, Reck M, Mansfield AS, et al. Updated Overall Survival and PD-L1 Subgroup Analysis of Patients With Extensive-Stage Small-Cell Lung Cancer Treated With Atezolizumab, Carboplatin, and Etoposide (IMpower133). *Journal*

of clinical oncology : official journal of the American Society of Clinical Oncology. 2021;39(6):619-630.

21. Luo H, Lu J, Bai Y, et al. Effect of Camrelizumab vs Placebo Added to Chemotherapy on Survival and Progression-Free Survival in Patients With Advanced or Metastatic Esophageal Squamous Cell Carcinoma: The ESCORT-1st Randomized Clinical Trial. *JAMA.* 2021;326(10):916-925.
22. Mai HQ, Chen QY, Chen D, et al. Toripalimab or placebo plus chemotherapy as first-line treatment in advanced nasopharyngeal carcinoma: a multicenter randomized phase 3 trial. *Nat Med.* 2021;27(9):1536-1543.
23. Miles D, Gligorov J, Andre F, et al. Primary results from IMpassion131, a double-blind, placebo-controlled, randomised phase III trial of first-line paclitaxel with or without atezolizumab for unresectable locally advanced/metastatic triple-negative breast cancer. *Ann Oncol.* 2021;32(8):994-1004.
24. Moehler M, Dvorkin M, Boku N, et al. Phase III Trial of Avelumab Maintenance After First-Line Induction Chemotherapy Versus Continuation of Chemotherapy in Patients With Gastric Cancers: Results From JAVELIN Gastric 100. *Journal of clinical oncology : official journal of the American Society of Clinical Oncology.* 2021;39(9):966-977.
25. Monk BJ, Colombo N, Oza AM, et al. Chemotherapy with or without avelumab followed by avelumab maintenance versus chemotherapy alone in patients with previously untreated epithelial ovarian cancer (JAVELIN Ovarian 100): an open-label, randomised, phase 3 trial. *The Lancet Oncology.* 2021;22(9):1275-1289.
26. Moore KN, Bookman M, Sehouli J, et al. Atezolizumab, Bevacizumab, and Chemotherapy for Newly Diagnosed Stage III or IV Ovarian Cancer: Placebo-Controlled Randomized Phase III Trial (IMagyn050/GOG 3015/ENGOT-OV39). *Journal of clinical oncology : official journal of the American Society of Clinical Oncology.* 2021;39(17):1842-1855.
27. Nishio M, Barlesi F, West H, et al. Atezolizumab Plus Chemotherapy for First-Line Treatment of Nonsquamous NSCLC: Results From the Randomized Phase 3 IMpower132 Trial. *J Thorac Oncol.* 2021;16(4):653-664.
28. Owonikoko TK, Park K, Govindan R, et al. Nivolumab and Ipilimumab as Maintenance Therapy in Extensive-Disease Small-Cell Lung Cancer: CheckMate 451. *Journal of clinical oncology : official journal of the American Society of Clinical Oncology.* 2021;39(12):1349-1359.
29. Reck M, Ciuleanu TE, Cobo M, et al. First-line nivolumab plus ipilimumab with two cycles of chemotherapy versus chemotherapy alone (four cycles) in advanced non-small-cell lung cancer: CheckMate 9LA 2-year update. *ESMO Open.* 2021;6(5):100273.
30. Rodriguez-Abreu D, Powell SF, Hochmair MJ, et al. Pemetrexed plus platinum with or without pembrolizumab in patients with previously untreated metastatic nonsquamous NSCLC: protocol-specified final analysis from KEYNOTE-189. *Ann Oncol.* 2021;32(7):881-895.
31. Sugawara S, Lee JS, Kang JH, et al. Nivolumab with carboplatin, paclitaxel, and bevacizumab for first-line treatment of advanced nonsquamous non-small-cell lung cancer. *Ann Oncol.* 2021;32(9):1137-1147.
32. Zhou C, Chen G, Huang Y, et al. Camrelizumab plus carboplatin and pemetrexed versus chemotherapy alone in chemotherapy-naive patients with advanced non-squamous non-small-cell lung cancer (CameL): a randomised, open-label, multicentre, phase 3 trial. *Lancet Respir Med.* 2021;9(3):305-314.
33. Motzer R, Alekseev B, Rha SY, et al. Lenvatinib plus Pembrolizumab or Everolimus for Advanced Renal Cell Carcinoma. *The New England journal of medicine.* 2021;384(14):1289-1300.
34. Powles T, Csoszi T, Ozguroglu M, et al. Pembrolizumab alone or combined with chemotherapy versus chemotherapy as first-line therapy for advanced urothelial carcinoma (KEYNOTE-361): a randomised, open-label, phase 3 trial. *The Lancet Oncology.* 2021;22(7):931-945.
35. Sun JM, Shen L, Shah MA, et al. Pembrolizumab plus chemotherapy versus chemotherapy alone for first-line treatment of advanced oesophageal cancer (KEYNOTE-590): a randomised, placebo-controlled, phase 3 study. *Lancet.* 2021;398(10302):759-771.
36. Burtness B, Rischin D, Greil R, et al. Pembrolizumab Alone or With Chemotherapy for Recurrent/Metastatic Head and Neck Squamous Cell Carcinoma in KEYNOTE-048: Subgroup Analysis by Programmed Death Ligand-1 Combined Positive Score. *Journal of clinical oncology : official journal of the American Society of Clinical Oncology.* 2022;40(21):2321-2332.
37. Cortes J, Rugo HS, Cescon DW, et al. Pembrolizumab plus Chemotherapy in Advanced Triple-Negative Breast Cancer. *The New England journal of medicine.* 2022;387(3):217-226.
38. Doki Y, Ajani JA, Kato K, et al. Nivolumab Combination Therapy in Advanced Esophageal Squamous-Cell Carcinoma. *The New England journal of medicine.* 2022;386(5):449-462.

39. Paz-Ares LG, Ramalingam SS, Ciuleanu TE, et al. First-Line Nivolumab Plus Ipilimumab in Advanced NSCLC: 4-Year Outcomes From the Randomized, Open-Label, Phase 3 CheckMate 227 Part 1 Trial. *J Thorac Oncol.* 2022;17(2):289-308.
40. Baas P, Scherpereel A, Nowak AK, et al. First-line nivolumab plus ipilimumab in unresectable malignant pleural mesothelioma (CheckMate 743): a multicentre, randomised, open-label, phase 3 trial. *Lancet.* 2021;397(10272):375-386.
41. Spigel DR, Faivre-Finn C, Gray JE, et al. Five-Year Survival Outcomes From the PACIFIC Trial: Durvalumab After Chemoradiotherapy in Stage III Non-Small-Cell Lung Cancer. *Journal of clinical oncology : official journal of the American Society of Clinical Oncology.* 2022;40(12):1301-1311.
42. Wolchok JD, Chiarion-Sileni V, Gonzalez R, et al. Long-Term Outcomes With Nivolumab Plus Ipilimumab or Nivolumab Alone Versus Ipilimumab in Patients With Advanced Melanoma. *Journal of clinical oncology : official journal of the American Society of Clinical Oncology.* 2022;40(2):127-137.
43. Yau T, Park JW, Finn RS, et al. Nivolumab versus sorafenib in advanced hepatocellular carcinoma (CheckMate 459): a randomised, multicentre, open-label, phase 3 trial. *The Lancet Oncology.* 2022;23(1):77-90.
44. Zhou C, Wang Z, Sun Y, et al. Sugemalimab versus placebo, in combination with platinum-based chemotherapy, as first-line treatment of metastatic non-small-cell lung cancer (GEMSTONE-302): interim and final analyses of a double-blind, randomised, phase 3 clinical trial. *The Lancet Oncology.* 2022;23(2):220-233.
45. Cheng AL, Qin S, Ikeda M, et al. Updated efficacy and safety data from IMbrave150: Atezolizumab plus bevacizumab vs. sorafenib for unresectable hepatocellular carcinoma. *J Hepatol.* 2022;76(4):862-873.
46. Gogishvili M, Melkadze T, Makharadze T, et al. Cemiplimab plus chemotherapy versus chemotherapy alone in non-small cell lung cancer: a randomized, controlled, double-blind phase 3 trial. *Nat Med.* 2022;28(11):2374-2380.
47. Kang YK, Chen LT, Ryu MH, et al. Nivolumab plus chemotherapy versus placebo plus chemotherapy in patients with HER2-negative, untreated, unresectable advanced or recurrent gastric or gastro-oesophageal junction cancer (ATTRACTION-4): a randomised, multicentre, double-blind, placebo-controlled, phase 3 trial. *The Lancet Oncology.* 2022;23(2):234-247.
48. Lu Z, Wang J, Shu Y, et al. Sintilimab versus placebo in combination with chemotherapy as first line treatment for locally advanced or metastatic oesophageal squamous cell carcinoma (ORIENT-15): multicentre, randomised, double blind, phase 3 trial. *BMJ.* 2022;377:e068714.
49. Motzer RJ, Powles T, Burotto M, et al. Nivolumab plus cabozantinib versus sunitinib in first-line treatment for advanced renal cell carcinoma (CheckMate 9ER): long-term follow-up results from an open-label, randomised, phase 3 trial. *The Lancet Oncology.* 2022;23(7):888-898.
50. Wang J, Zhou C, Yao W, et al. Adebrelimab or placebo plus carboplatin and etoposide as first-line treatment for extensive-stage small-cell lung cancer (CAPSTONE-1): a multicentre, randomised, double-blind, placebo-controlled, phase 3 trial. *The Lancet Oncology.* 2022;23(6):739-747.
51. Cheng Y, Han L, Wu L, et al. Effect of First-Line Serplulimab vs Placebo Added to Chemotherapy on Survival in Patients With Extensive-Stage Small Cell Lung Cancer: The ASTRUM-005 Randomized Clinical Trial. *JAMA.* 2022;328(12):1223-1232.
52. Johnson ML, Cho BC, Luft A, et al. Durvalumab With or Without Tremelimumab in Combination With Chemotherapy as First-Line Therapy for Metastatic Non-Small-Cell Lung Cancer: The Phase III POSEIDON Study. *Journal of clinical oncology : official journal of the American Society of Clinical Oncology.* 2023;41(6):1213-1227.
53. Dummer R, Long GV, Robert C, et al. Randomized Phase III Trial Evaluating Spartalizumab Plus Dabrafenib and Trametinib for BRAF V600-Mutant Unresectable or Metastatic Melanoma. *Journal of clinical oncology : official journal of the American Society of Clinical Oncology.* 2022;40(13):1428-1438.
54. Wu HX, Pan YQ, He Y, et al. Clinical Benefit of First-Line Programmed Death-1 Antibody Plus Chemotherapy in Low Programmed Cell Death Ligand 1-Expressing Esophageal Squamous Cell Carcinoma: A Post Hoc Analysis of JUPITER-06 and Meta-Analysis. *Journal of clinical oncology : official journal of the American Society of Clinical Oncology.* 2022:JCO2201490.
55. Wang ZX, Cui C, Yao J, et al. Toripalimab plus chemotherapy in treatment-naive, advanced esophageal squamous cell carcinoma (JUPITER-06): A multi-center phase 3 trial. *Cancer Cell.* 2022;40(3):277-288 e273.
56. de Castro G, Jr., Rizvi NA, Schmid P, et al. NEPTUNE: Phase 3 Study of First-Line Durvalumab Plus Tremelimumab in

Patients With Metastatic NSCLC. *J Thorac Oncol.* 2023;18(1):106-119.
